# Wave morphing towards the flat-top envelope in photonics systems driven by artificial gauge fields

**DOI:** 10.1038/s41467-026-76042-0

**Published:** 2026-07-29

**Authors:** Peishen Li, Xiaoyu Zhang, Feifan Wang, Ye Chen, Xuefan Yin, Chao Peng

**Affiliations:** 1 State Key Laboratory of Photonics and Communications, School of Electronics & Frontiers Science Center for Nano-optoelectronics, Peking University, Beijing, China; 2 Peng Cheng Laboratory, Shenzhen, China

**Keywords:** Nanophotonics and plasmonics, Photonic crystals

## Abstract

In quantum physics, classical optics, and many other wave systems, wave confinement in a finite domain leads to discrete eigenstates that typically exhibit spatially nonuniform, oscillatory profiles. A long-standing question is whether a confined system can counterintuitively support an eigenstate with a uniform, nonzero envelope, offering new opportunities for quantum emitters, optical antennas, and lasers. Here, we show that such a state can be realized through spatial phase engineering that acts as an artificial gauge potential. By continuously tuning the accumulated phase, the eigenvalue spectra undergo a spectral flow that reshapes the profiles of the eigenstates, enabling the formation of a flat-top state with a uniform yet nontrivial envelope. We implement this concept in a photonic crystal slab, where a central bulk region is surrounded by heterogeneous band gaps that tailor reflection phases to serve as an artificial local gauge field. By inducing single-mode lasing, we probe the morphing of mode envelope profiles, demonstrating a continuous transition from conventional oscillatory states to a flat-top state via near- and far-field measurements.

## Abstract (web summary)

Whether a confined wave system can support a uniform, nonzero eigenstate has long been a counterintuitive question. Here, authors answer it by using artificial gauge fields from boundary phases to morph conventional modes into a flat-top state, and demonstrate the morphing via single-mode lasing in photonic crystals.

## Introduction

In a homogeneous, lossless medium, a wave propagates indefinitely without distortion, preserving constant amplitude and a unidirectional flow of energy. When confined within a finite domain, however, boundary-induced reflections lead to counter-propagation and the formation of standing waves, which typically exhibit nonuniform spatial field distributions with distinct nodes and antinodes. Such confinement gives rise to discrete, quantized eigenstates $\left\vert n\right\rangle$, a phenomenon common to quantum^1–5^, optical^6–10^, and acoustic^11–13^ resonant systems. For eigenstates with higher energy, their spatially varying envelope becomes more pronounced, with increasing numbers of nodes and antinodes. Even the fundamental state that is free of nodes still exhibits a spatially varying profile rather than a uniform distribution. Consequently, realizing a state that maintains a nonzero, spatially uniform envelope is nontrivial under conventional confinement. In the literature, flat-top or plateau-like wave distributions have been observed in sophisticated systems, such as photon Bose-Einstein condensates enabled by light-matter interactions^14–17^, Wannier-Stark localization with engineered phase gradients^18–23^, and photonic crystals (PhCs) engineered to exhibit zero group index^24,25^. Similar phenomena have also been reported in topological systems with non-Hermitian skin effect^26–29^ as well as gap solitons in nonlinear lattices^30–33^. These breakthroughs highlight remarkable opportunities for both fundamental science and emerging technologies, including quantum states with uniform probability distributions over finite regions, self-focusing beams, and spatially uniform light emission for scalable lasers.

We notice that a particularly straightforward way to realize a state with a uniform, nonzero envelope is to employ a ring geometry with periodic boundary conditions (PBCs). In such a system, the $\left\vert 0\right\rangle$ state with zero wavevector represents a constant-phase solution with a nonzero uniform field distribution. According to the Aharonov-Bohm effect^34–38^, a magnetic flux threading the ring introduces a gauge potential and thus imparts an additional geometric phase, modifying the phase accumulation of the wavefunction along the closed path. This results in a shift of the effective momentum, thereby continuously and periodically tuning the eigenstates and the corresponding energy levels. This simple yet robust mechanism provides an elegant means of manipulating the properties of eigenstates and is closely related to the Byers-Yang theorem^39^ which establishes that physical observables in a ring geometry vary periodically with the enclosed magnetic flux^40–47^. Importantly, because the accumulated phase originates from a gauge potential, this concept is not restricted to electronic systems or real magnetic fields. Through artificial gauge potentials, similar phase engineering can be implemented in a broad class of wave systems^48–55^, including photonic platforms.

Here, we propose a general confined photonic wave system, in which the reflection phases at the boundaries act as an artificial gauge potential that shifts the energy levels, eventually morphing the energy state to an unusual fundamental state that maintains a flat-top envelope. Specifically, we encircle a bulk PhC slab with a boundary region, in which counter-propagating waves are connected in an Ouroboros-like configuration that is topologically equivalent to a ring structure, allowing the eigenstates to evolve continuously and periodically with respect to boundary reflection phases. We design and fabricate samples on a III-V/Si hybrid-bonded wafer and realize single-mode lasing action to probe the underlying eigenstates. The trajectory of energy-level transitions is tracked, and their asymptotic morphing toward a flat-top state is evidenced by observing their distinct lasing patterns.

## Results

## Principle and design

**Fig. 1:**
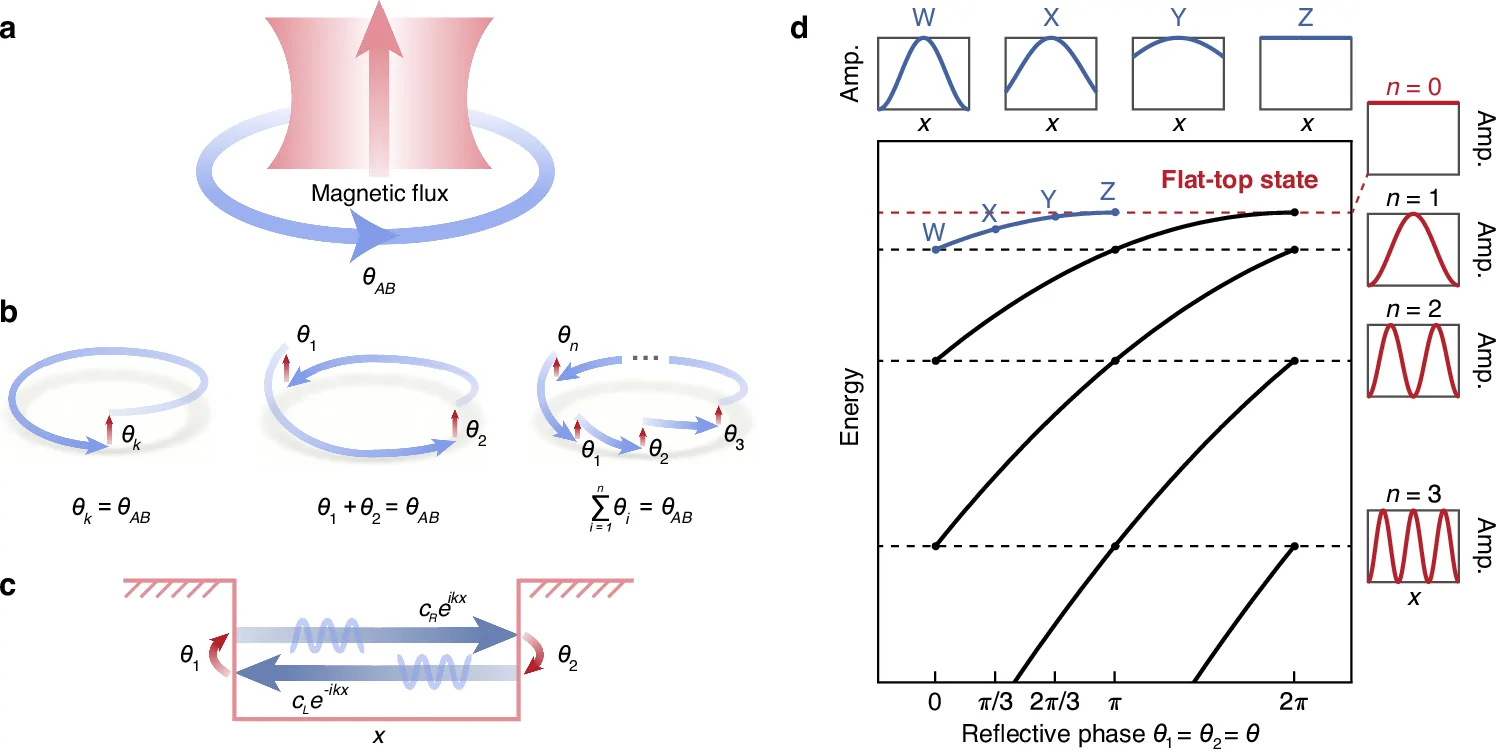
Principle of spectral flow and wave morphing enabled by artificial gauge potential. **a** Schematic of a ring structure threaded by a magnetic flux, which creates a geometric phase *θ*_*AB*_ upon wavefunctions according to the Aharonov-Bohm effect. **b** An effective ring model with different phase jump configurations acting as artificial local gauge fields, including one phase jump *θ*_*k*_ (left); two phase jumps *θ*_*k*1_ and *θ*_*k*2_ (middle); multiple phase jumps *θ*_*k*,*i*_ (right). The cumulative phase shift ${\theta }_{{{\rm{tot}}}}=\mathop{\sum }_{i=1}^{N}{\theta }_{k,i}={\theta }_{AB}$ represents an effective magnetic flux. **c** Schematic of a 1D finite cavity, equivalent to the ring model with two phase jumps (middle panel, b). Two counter-propagating waves (*c*_*R*_*e*^*ikx*^ and *c*_*L*_*e*^−*ikx*^) are reflected by the boundaries with additional phases *θ*_1,2_, forming a double Ouroboros-like configuration. **d** Evolutions of energy levels ($\left\vert n=1,2,3\right\rangle$) with respect to reflection phase *θ* = *θ*_1_ = *θ*_2_ (left panel). When *θ* increases from 0 to 2*π*, the effective magnetic flux *θ*_tot_ = 2*θ* winds by 4*π*, making the entire set of energy levels shift by two levels, while the eigenstates evolve for a complete cycle. Especially, $\left\vert 1\right\rangle$ shifts to the fundamental state $\left\vert 0\right\rangle$ with a flat-top envelope when *θ* = *π*. The evolution from $\left\vert 1\right\rangle$ into $\left\vert 0\right\rangle$ is highlighted in blue. The envelopes of four levels ($\left\vert n=0,1,2,3\right\rangle$) at the initial phase *θ* = 0 (right panel), together with the envelopes of eigenstates at *θ* = 0, *π*/3, 2*π*/3, and *π* on blue trajectory (top panel, labeled W-Z), are plotted.

We start by considering an artificial ring model (Fig. 1a), where a magnetic flux threads a ring of circumference 2*L*. Under periodic boundary conditions *ψ*(*x* + 2*L*) = *ψ*(*x*), the energy spectrum shows as ${E}_{n}\propto {(n-{\theta }_{AB}/2\pi )}^{2}/{L}^{2}$, where *θ*_*AB*_ is the Aharonov-Bohm phase and *n* labels the eigenstate $\left\vert n\right\rangle$ with wavefunction *ψ*_*n*_. The spectrum is periodic in *θ*_*AB*_, as a direct manifestation of the Byers-Yang theorem, and exhibits a continuous spectral flow as the flux is varied. Furthermore, the Aharonov-Bohm phase can be incorporated into the wavefunctions, acting as a continuously accumulated phase along the ring. With an appropriate gauge choice, this accumulated phase can be equivalently represented by a kink of phase jump *θ*_*k*_ = *θ*_*AB*_ which modifies the boundary condition to $\psi (x+2L)=\psi (x){e}^{-i{\theta }_{k}}$ (left panel, Fig. 1b), without altering the energy spectrum. In general, multiple phase jumps can be introduced in the wavefunction along the ring (middle and right panels, Fig. 1b), with their total phase accumulation *θ*_tot_ = *θ*_*AB*_ playing the role of an effective flux. In this picture, only the total phase is physically relevant, while the rearrangement of spatial phase jumps corresponds to a gauge transformation. Thus, the spatial variation of the phase effectively acts as an artificial gauge potential (see Supplementary Section 1 for details).

Without loss of generality, we consider a ring model with two phase jumps *θ*_1_ and *θ*_2_ (middle panel, Fig. 1b), which is equivalent to a finite one-dimensional (1D) cavity of length *L*, bounded by two perfectly reflective boundaries with nontrivial reflection phases (Fig. 1c). In such a system, the wavefunction takes the form *ψ*_1*D*_ = *c*_*R*_*e*^*ikx*^ + *c*_*L*_*e*^−*ikx*^ as a superposition of left- and right-propagating waves. This yields the boundary conditions ${c}_{R}=-{c}_{L}{e}^{i{\theta }_{1}}$ and ${c}_{L}=-{c}_{R}{e}^{i{\theta }_{2}}{e}^{2ikL}$ and the quantization condition *θ*_tot_ + 2*kL* = 2*nπ*, where *θ*_*tot*_ = *θ*_1_ + *θ*_2_ = *θ*_*AB*_ acts as the effective flux. In this way, the gauge-induced phase accumulation in the wavefunction manifests as a relative phase shift between counter-propagating waves. The energy spectra periodically depend upon the total phase *θ*_tot_, exhibiting a continuous spectral flow as *θ*_tot_ is varied (left panel, Fig. 1d). For simplicity, we set *θ*_1_ = *θ*_2_ = *θ* and examine several low-order states (i.e. $\left\vert n=1,2,3\right\rangle$). Their spatial profiles at *θ* = 0 are plotted in the right panel of Fig. 1d, showing the characteristic standing-wave patterns with nodes and antinodes. As *θ* increases from 0 to *π*, corresponding to a 2*π* winding of *θ*_tot_, all energy levels continuously evolve and connect to adjacent levels. Further increasing *θ* from *π* to 2*π* induces another full cycle, restoring the whole spectrum to the initial configuration.

Interestingly, in the aforementioned gauge configuration of *θ* = *π*, the fundamental state $\left\vert 0\right\rangle$ possesses a spatially uniform wavefunction *ψ*_0_ = const ≠ 0 across the finite-sized cavity. To illustrate this, we track the evolution trajectory connecting eigenstate $\left\vert 1\right\rangle$ and $\left\vert 0\right\rangle$ as *θ* is varied from 0 to *π* (blue line, Fig. 1d), and present the corresponding spatial profiles at *θ* = 0, *π*/3, 2*π*/3, and *π*, respectively (labeled as W-Z in the top panel of Fig. 1d). As *θ* increases, the effective wavevector *k* is continuously decreased, leading to a gradual reduction of spatial oscillations in the wavefunction profile. At *θ* = *π*, the wavevector reaches zero, and the profile becomes spatially uniform with a constant phase distribution inside the cavity, corresponding to a vanishing net energy flow. From a mathematical perspective, such an unusual phenomenon can be understood in terms of an effective homogeneous Neumann condition $(\psi ^{\prime}=0)$ at the boundaries of a finite 1D cavity, which naturally supports a fundamental state $\left\vert 0\right\rangle$ with a constant, nonzero spatial profile. Different from Dirichlet conditions, which force the wavefunctions to vanish at the boundaries, Neumann conditions impose zero gradient, corresponding to vanishing net flux across the boundaries. This permits the wavefunction to remain finite at the boundaries and extend uniformly across the entire domain. Further details are provided in Supplementary Section 2.

**Fig. 2:**
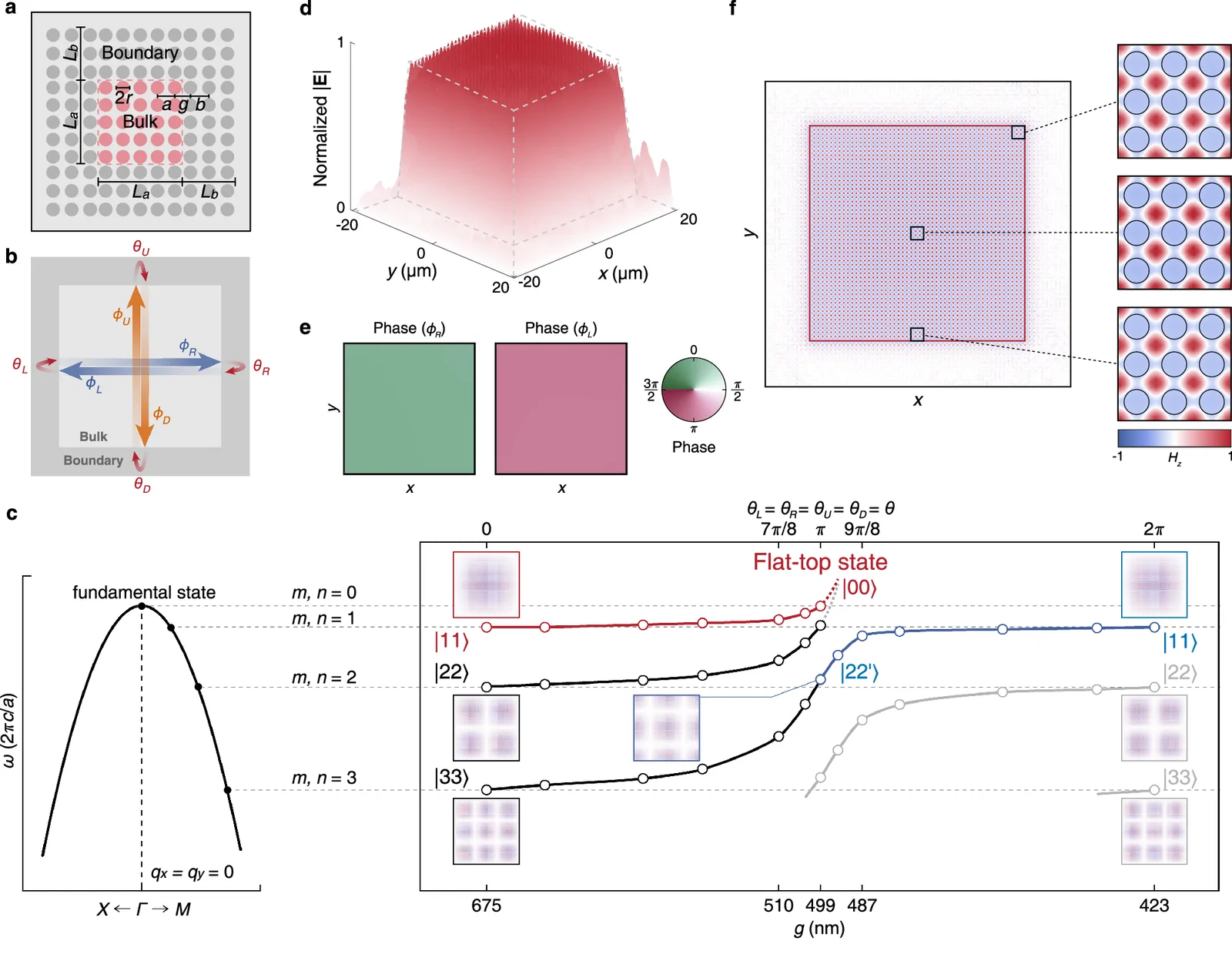
A 2D heterogeneous PhC realization. **a** Schematic of 2D heterogeneous PhC utilized to simulate the artificial local gauge fields, consisting of a bulk region and a boundary region. **b** Schematic of the partial-wave propagation. Four partial waves are grouped into two sets: *ϕ*_*R*,*L*_ (blue arrows) and *ϕ*_*U*,*D*_ (yellow arrows). The reflection phases are denoted as *θ*_*R*,*L*,*U*,*D*_. **c** Dispersion of a Bloch mode (left panel) with gray dashed lines, with four quantized energy levels (*m* = *n* = 0, 1, 2, 3) at *g* = 675 nm. The evolution of transverse cavity modes $\left\vert m,n=m\right\rangle$ (right panel) is calculated as *θ*_*R*,*L*,*U*,*D*_ = *θ* varies by reducing *g*. When *θ* increases from 0 (*g* = 675 nm) to 2*π* (*g* = 423 nm), a complete evolution cycle is accomplished, as every energy level blue-shifts by two levels. A flat-top mode $\left\vert 00\right\rangle$ is realized at *θ* = *π* (*g* = 499 nm). The field distributions of transverse modes are shown as insets. Evolutions from $\left\vert 11\right\rangle \to \left\vert 00\right\rangle$ (red dotted) and $\left\vert 22^{\prime} \right\rangle \to \left\vert 11\right\rangle$ (blue dotted) are plotted. **d** Envelope of the fundamental mode $\left\vert 00\right\rangle$, with the gray dashed lines marking the boxcar-like contour. **e** Phase distribution of partial waves *ϕ*_*R*,*L*_ for $\left\vert 00\right\rangle$. **f** Field distribution of $\left\vert 00\right\rangle$ in the entire PhC cavity (left panel), with zoomed-in field distributions at its center, edge, and corner (right panels). All results are calculated by using numerical simulation (COMSOL Multiphysics).

To realize an artificial gauge potential in a realistic setting, a general approach is to introduce a continuous distributed permittivity modulation, which creates a spatial phase gradient and hence a controlled phase accumulation. Here we adopt a more fabrication-friendly implementation, in which perfectly reflective boundaries with engineered reflection phase shifts effectively produce the required phase accumulation in a compact form. We hereby consider a two-dimensional (2D) heterogeneous PhC structure (Fig. 2a), consisting of a central bulk region enclosed by a surrounding boundary region. Both regions are patterned as square lattices of circular air holes whose sizes are given by *L*_*a*_ and *L*_*b*_, respectively. Their lattice constants *a* and *b* are deliberately detuned such that one bulk band near the second *Γ* point is embedded within the photonic bandgap (PBG) of the boundary region. The reflection phases can be readily tuned by varying the spacing *g* between the two regions. This configuration closely mimics a quantum dot, i.e., a finite-sized confined wave system, as we discussed previously^56–58^. We note that the continuous-modulation scheme without relying on boundary manipulation can also be realized in such a heterogeneous PhC structure. More details are provided in Supplementary Section 3.

Next, we show that such a heterogeneous PhC cavity is equivalent to the artificial ring model presented in Fig. 1. In such a confined cavity, a Bloch band becomes discretized into a set of energy levels, each corresponding to a transverse cavity mode. These cavity modes can be approximated as a superposition of two pairs of counter-propagating partial waves $[{\phi }_{R}{e}^{i{\beta }_{0}x},\,{\phi }_{L}{e}^{-i{\beta }_{0}x},\,{\phi }_{U}{e}^{i{\beta }_{0}y},\,{\phi }_{D}{e}^{-i{\beta }_{0}y}]$^59–61^, as illustrated in Fig. 2b, where *β*_0_ = 2*π*/*a* is a rapidly varying wavevector associated with the Bloch band. These waves are reflected at the boundaries due to the PBG effect, acquiring slowly varying envelopes^56,58,60^. These partial waves can be grouped into two sets: *ϕ*_*R*,*L*_ (blue arrows) and *ϕ*_*U*,*D*_ (yellow arrows), governed by the reflective boundaries in different directions. Moreover, the waves within each set do not scatter into the other at the boundaries, and thus this 2D system decomposes into two independent 1D subsystems. As an example, we focus on one subsystem of ${\phi }_{R,L}(x)=[{c}_{R,\,L}^{+}\exp (i{q}_{x}x)+{c}_{R,\,L}^{-}\exp (-i{q}_{x}x)]$, where ${c}_{R,\,L}^{+, -}$ are superposition coefficients and *q*_*x*_ is a slowly varying wavevector, following *q*_*x*_ ~ *π*/*L*_*a*_ ≪ *β*_0_. The reflection between the counter-propagating waves forms a closed circulation in which the reflection phases *θ*_*R*,*L*_ act as a local gauge field, topologically equivalent to the ring model in Fig. 1. A similar argument holds for *ϕ*_*U*,*D*_, leading to the following quantization conditions:

1 $$\begin{array}{r}2{q}_{x}{L}_{a}+{\Theta }_{x}({\theta }_{R}+{\theta }_{L})=2m\pi \quad 2{q}_{y}{L}_{a}+{\Theta }_{y}({\theta }_{U}+{\theta }_{D})=2n\pi \end{array}$$

in which Θ_*x*,*y*_ account for the effective phase accumulation along the *x* and *y* directions, respectively, playing a role analogous to an artificial magnetic flux in the ring model. Integers *m* and *n* are quantization indices along the *x* and *y* directions, respectively, and label the transverse cavity modes as $\left\vert mn\right\rangle$. Physically, larger *m* or *n* corresponds to stronger spatial oscillations of the envelope along the respective direction. Details are elaborated in Supplementary Section 4.

To quantitatively demonstrate the spectral flow, we numerically compute the transverse modes $\left\vert mn\right\rangle$ for different values of spacing *g* (Fig. 2c). For simplicity, we consider the symmetric case *m* = *n* and identical spacing gaps in all directions, such that *θ*_*R*,*L*,*U*,*D*_ = *θ* and *Θ*_*x*,*y*_ = *Θ*. As a reference, the dispersion of the relevant Bloch band and the corresponding discrete energy levels (*m* = *n* = 0, 1, 2, 3, gray dashed lines) at *Θ* = *θ* = 0 are plotted in the left panel of Fig. 2c. Here, the modal energy is defined relative to the band edge and is characterized by slowly varying wavevectors *q*_*x*,*y*_, following ${E}_{{{\rm{eff}}}}\propto {q}_{x}^{2}+{q}_{y}^{2}$. In this presentation, the $\left\vert 00\right\rangle$ mode represents the "zero-energy" fundamental mode, since its wavevectors vanish (*q*_*x*_ = *q*_*y*_ = 0), corresponding to the band edge.

The spectral-flow trajectories are shown in the right panel of Fig. 2c. At *θ* = 0 (*g* = 675 nm), three transverse modes $\left\vert 11\right\rangle$, $\left\vert 22\right\rangle$ and $\left\vert 33\right\rangle$ are identified, whose field envelopes possess distinct oscillatory patterns with characteristic antinodes that define the quantization indices *m* = *n* ∈ {1, 2, 3}. As *g* decreases to 499 nm, the reflection phase increases to *θ* = *π*, corresponding to an effective magnetic flux of *Θ* = 2*π*, which shifts the entire spectrum upward by one level. In particular, $\left\vert 11\right\rangle$ mode continuously evolves into the fundamental mode $\left\vert 00\right\rangle$. As shown in Fig. 2d, $\left\vert 00\right\rangle$ mode exhibits a flat-top-like envelope, remaining nearly uniform within the bulk region owing to *q*_*x*,*y*_ = 0, and rapidly decaying near the boundaries. Moreover, all partial waves belonging to $\left\vert 00\right\rangle$ mode display a uniform phase distribution within the bulk region (Fig. 2e), and the phase difference between two counter-propagating partial waves (i.e., *ϕ*_*R*,*L*_) remains approximately constant ($\arg {\phi }_{R}/{\phi }_{L}\approx \pi$) throughout the bulk. This phase difference matches the reflection phase required for $\left\vert 00\right\rangle$ (*θ* = *π*). Notably, $\left\vert 00\right\rangle$ mode possesses nearly identical field distributions at the center, edges, and corners of the bulk region (Fig. 2f), indicating an isotropically suppressed group velocity and vanishing net energy flow in all lateral directions. The detailed trajectories can be found in Supplementary Sections 5 and 6.

Meanwhile, we track the evolution of transverse modes, observing transitions from $\left\vert 22\right\rangle \to \left\vert 11^{\prime} \right\rangle$ and from $\left\vert 33\right\rangle \to \left\vert 22^{\prime} \right\rangle$. Notably, while $\left\vert 22\right\rangle$ at *θ* = 0 and $\left\vert 22^{\prime} \right\rangle$ at *θ* = *π* possess the same modal energy, their spatial field envelopes are different, indicating that tuning the reflection phase from *θ* = 0 → *π* (corresponding to *Θ* = 0 → 2*π*) does not yet complete a full evolution cycle. Namely, although the spectra return to the original set of energies, their spatial distributions are not restored. As the phase further increases from *θ* = *π* → 2*π* by reducing *g* from 499 nm to 423 nm, the effective flux *Θ* accumulates an additional 2*π*, causing a further spectral shift by one more level. Consequently, at *θ* = 2*π*, the system is restored to its initial configuration at *θ* = 0, both in energy levels and field patterns, thereby completing a full evolution cycle. In Fig. 2c, one complete spectral-flow cycle is highlighted, consisting of two branches: $\left\vert 22^{\prime} \right\rangle$ to $\left\vert 11\right\rangle$ (blue dotted line), and $\left\vert 11\right\rangle$ to $\left\vert 00\right\rangle$ (red dotted line), respectively. We also build a theoretical model describing the relationship between *θ* and *g*, elaborated in Supplementary Section 7.

It is worth noting that, when *g* further decreases beyond 499 nm, the fundamental mode $\left\vert 00\right\rangle$ evolves into a special mode with frequency above the band edge, denoted as $\left\vert \varepsilon \right\rangle$. This mode has effectively “less energy” than the fundamental mode, and can no longer be fully described within a purely Hermitian ring model. Instead, it can be explained by a non-Hermitian ring model with complex slowly varying wavevector ${\tilde{q}}_{x,y}$. Indeed, incorporating non-Hermiticity is important when the reflection boundaries are not perfectly ideal, allowing energy leakage from the bulk into the boundary. Under such conditions, the system supports a mode that is predominantly localized near the bulk-boundary interface. We emphasize that this boundary-localization behavior differs from conventional non-Hermitian skin effect^26^, which typically arises from non-reciprocal hoppings in lattice systems. In contrast, the boundary-localization observed here originates from leakage-induced complexification of slowly varying wavevectors in a reciprocal photonic structure. Further details are provided in Supplementary Section 8.

## Sample fabrication and experimental observation

**Fig. 3:**
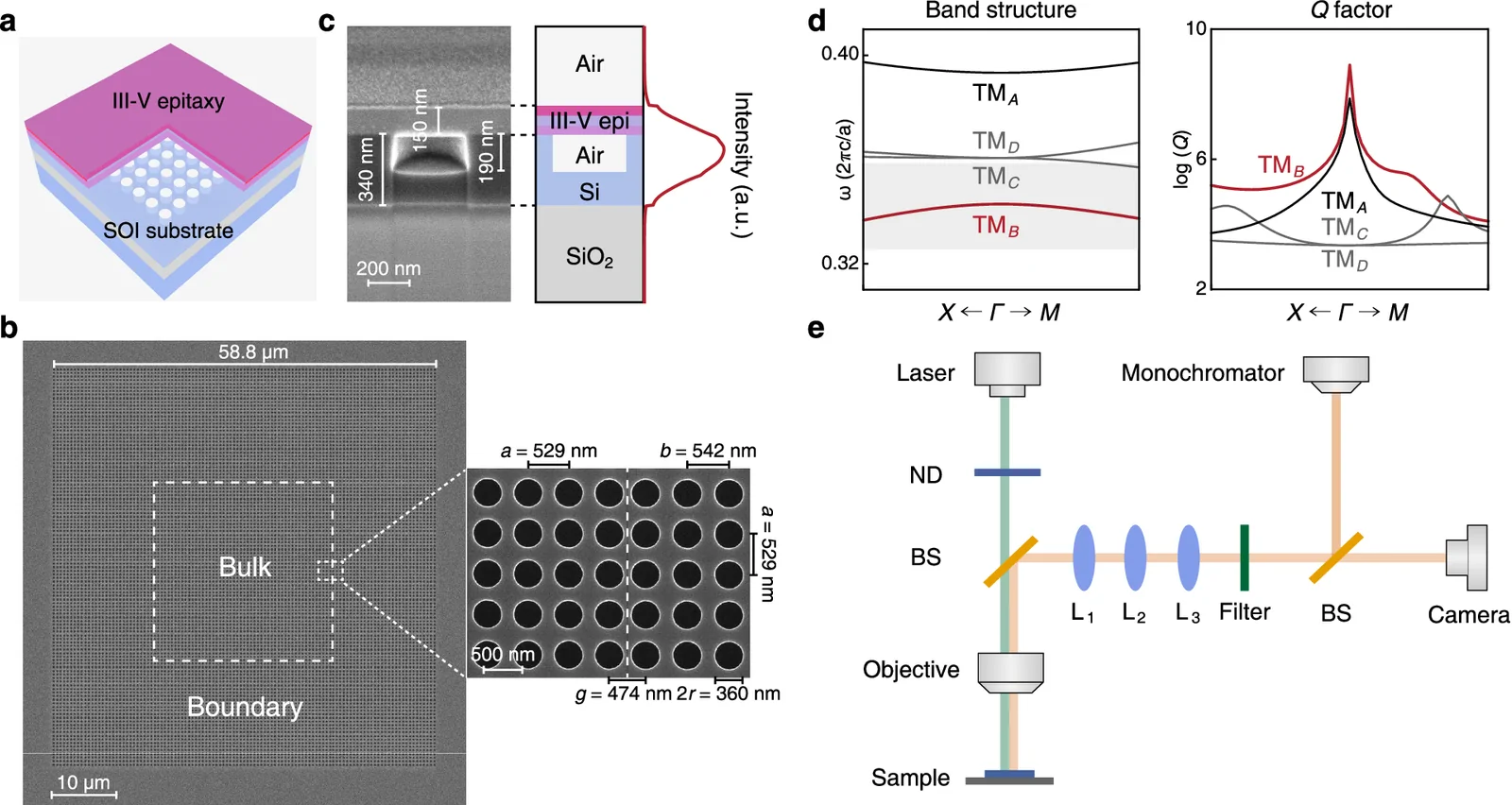
Sample design, fabrication and experimental setup. **a** Schematic of the designed sample on a hetero-bonded wafer, with an SOI patterned as PhCs, and III-V epitaxial layers providing optical gain. **b** Top view of the fabricated PhC (left panel), whose total size is 58.8 × 58.8 μm^2^, and the dashed box distinguishes the bulk region from the boundary region. The right panel shows the zoomed-in details. **c** Cross-sectional view of bulk PhC obtained by FIB cleaving (left), with its schematic provided for reference (right panel). The red line shows the cross-sectional profile of the TM_*B*_ mode at the *Γ* point as the lasing candidate. **d** Calculated band structures (left) and *Q* diagram (right panel) of the bulk region. Band TM_*B*_ is highlighted in red, and the photoluminescence range is gray-shaded. **e** Schematic of the measurement setup, with pumping light (green line) and lasing emission (orange lines). ND Neutral density filter, BS Beam splitter.

To experimentally observe the spectral flow and envelope morphing, we employ a hetero-bonded platform^62^ consisting of a silicon-on-insulator (SOI) wafer patterned as a PhC slab and a directly bonded InP-based epitaxial wafer that provides optical gain (Fig. 3a). We aim to achieve surface-emitting lasing to characterize the intrinsic properties of transverse modes through observing the lasing emission from the out-of-plane direction.

Specifically, fabrication begins with an SOI chip containing a 340 nm top silicon layer, on which the PhC pattern is defined by electron-beam lithography followed by inductively coupled plasma dry etching. By carefully controlling the etching time, the desired etching depth is achieved. In parallel, an InP-based epitaxial wafer is grown by metal-organic chemical vapor deposition, consisting of four pairs of multi-quantum wells sandwiched between two InGaAsP cladding layers to provide optical gain. The epitaxial wafer is cleaved into chips, then flipped and bonded to the SOI chip via a plasma-assisted bonding process. See Methods for more details about the fabrication process.

The fabricated sample is characterized using scanning electron microscopy (SEM). The top-view image (Fig. 3b) shows that the PhC has a total footprint of 58.8 × 58.8 μm^2^, comprising both the bulk and boundary regions as designed. A cross-sectional view obtained by focused ion beam (FIB) cleaving (left panel, Fig. 3c) reveals the multilayer structure of the bulk region, with a schematic provided for reference (middle panel). We calculate the band structures of the bulk region using three-dimensional (3D) numerical simulations based on the measured structural parameters (Fig. 3d). Four transverse-magnetic (TM) bands reside around the second *Γ* point, denoted as TM_*A*,*B*,*C*,*D*_, among which TM_*B*_ spectrally overlaps with the material photoluminescence range. To achieve stable single-mode lasing, we employ shallow etching to realize a quasi-mirror symmetry in the vertical direction. The cross-sectional optical intensity profile of the TM_*B*_ at the band edge (*Γ* point) is illustrated in the right panel of Fig. 3c, indicating that a significant portion of energy is confined within the active layer. Furthermore, a symmetry-protected bound state in the continuum (BIC)^63–65^ is supported at the band edge of TM_*B*_, which strongly suppresses vertical radiation loss and leads to an enhancement of the quality factor (*Q*) in its vicinity (right panel, Fig. 3d). Consequently, lower-order transverse modes derived from the TM_*B*_ band are expected to dominate the lasing action (see Supplementary Section 9 for details).

We use a confocal measurement system to characterize the fabricated samples (Fig. 3e). The samples are optically pumped at room temperature with a pulsed 1064-nm laser (green line). A 10× objective focuses the pump light onto the sample surface to initiate the out-of-plane lasing (yellow line). Both the lasing beam and reflected pump light are collected by the same objective and relayed through a cascaded 4*f* imaging system (objective + three lenses L_1−3_). A long-pass filter with a cutoff at 1300 nm removes the residual pump, after which the lasing pattern is recorded by a CMOS camera and the spectrum is measured by a monochromator. Both near-field patterns (NFPs) and far-field patterns (FFPs) of the lasing emission are obtained in the same setup by reconfiguring the imaging path using lens L_2_. Further details of the setup are provided in the Methods Section.

**Fig. 4:**
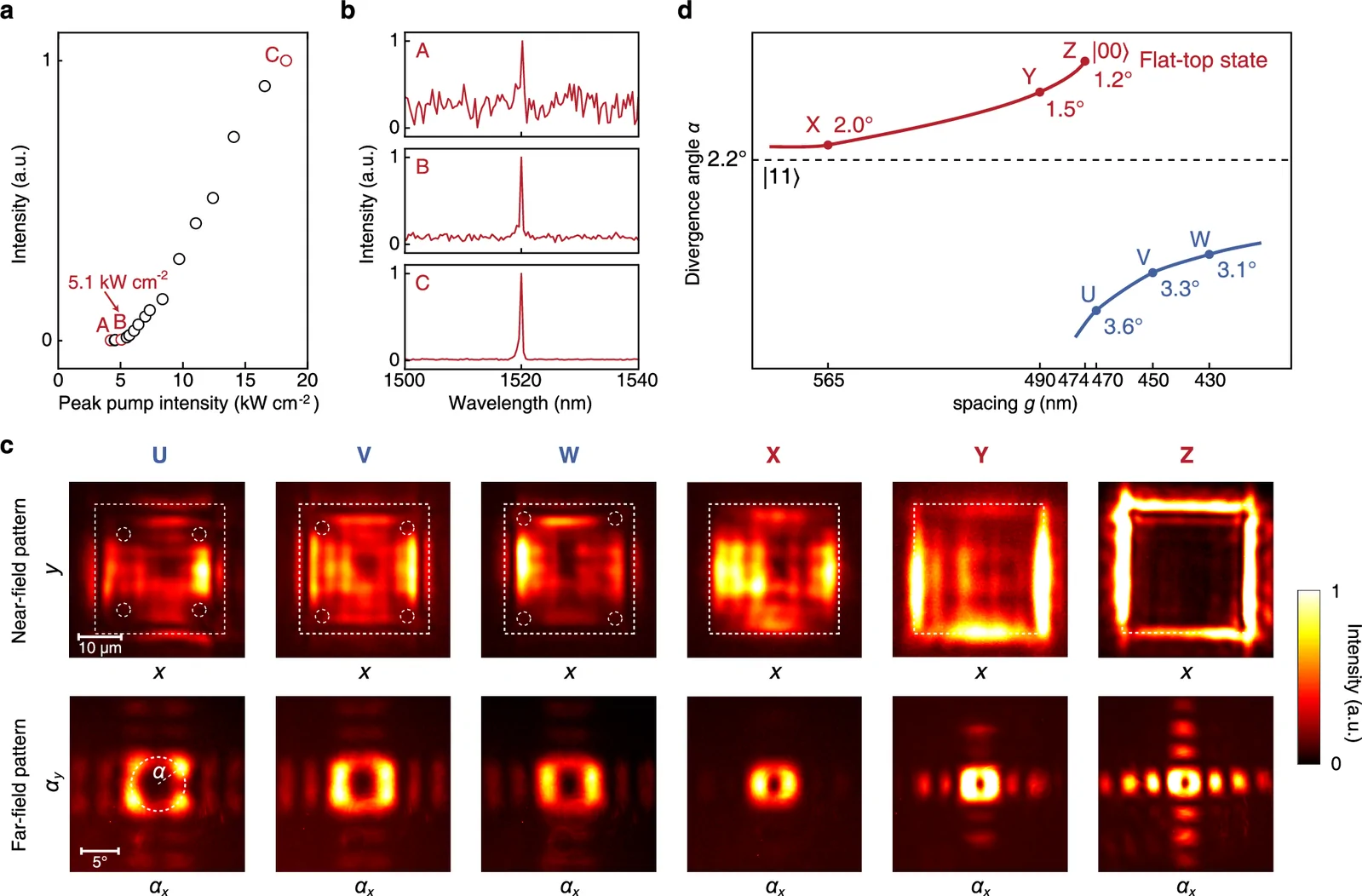
Experimental demonstration of spectral flow and wave morphing. **a** Measured light-output curve for sample Z with *g* = 474nm. Three points *A*, *B*, *C* are marked with pumping power of 4.2, 5.1, 18.3 kW/cm^2^, respectively, showing the status from spontaneous emission, threshold lasing, to stable single-mode lasing (wavelength of 1520 nm). **b** Measured lasing spectra corresponding to A (top), B (middle) and C (bottom), respectively. **c** Measured NFP (top panels) and FFP (bottom panels) of single-mode lasing for six samples with different *g*: U: 470 nm; V: 450 nm; W: 430 nm; X: 565 nm; Y: 490 nm; Z: 474 nm. From the divergence angles, U (3. 6^∘^), X (2. 0^∘^) and Z (1. 2^∘^) are identified as near-$\left\vert 22^{\prime} \right\rangle$, $\left\vert 11\right\rangle$ and near-$\left\vert 00\right\rangle$ modes, respectively. For sample Z (*g* = 474 nm), the FFP shrinks to its minimum (divergence angle of 1. 2^∘^) and the NFP appears almost dark within the cavity region, indicating the achievement of a near-flat-top envelope. In the NFP, the bulk-boundary interface is outlined by a dashed box, and dark nodes on the envelope are highlighted by dashed circles. In the FFP, the divergence angle *α* is indicated by a dashed circle. **d** Measured divergence angles of different samples as a function of spacing *g*, showing the evolutions of U-V-W (blue) and X-Y-Z (red). The dashed gray line represents the theoretical divergence angle for $\left\vert 11\right\rangle$ mode (2. 2^∘^).

To track the spectral flow, we fabricate six samples X, Y, Z, U, V, W by using the same fabrication process, but with different spacings *g* = 565, 490, 474, 470, 450, 430 nm, respectively. Other structural parameters are identical to those shown in Fig. 3b, c. The variation of *g* effectively modifies the reflection phases, thereby creating an artificial gauge potential. All samples exhibit stable single-mode lasing, dominated by the lowest-order transverse cavity mode. For clarity, sample Z (*g* = 474 nm) is taken as a representative example to demonstrate the lasing characteristics (Fig. 4a, b). The pump beam is adjusted to cover the entire PhC region, ensuring a uniform gain profile across the cavity region and minimizing gain-guiding effects, so that the reflection phase shift is primarily governed by the designed cavity boundaries. The light-output curve in Fig. 4a highlights three points (A–C), with corresponding spectra shown in Fig. 4b. At a low pump power of 4.2 kW/cm^2^ (A), only a weak resonant feature is observed above the spontaneous background. At point B, a pronounced peak emerges, indicating the onset of lasing with a threshold of 5.1 kW/cm^2^. Further increasing the pump power to 18.3 kW/cm^2^ (C) yields a sharp single peak, confirming stable single-mode lasing. These characteristics clearly distinguish lasing emission from non-lasing signals, evidenced by a well-defined threshold behavior. Results for other samples are provided in Supplementary Section 9.

Further, we measure the NFP (top panels) and FFP (bottom panels) for each sample when stable single-mode lasing is established (Fig. 4c). The NFPs reflect the spatial distribution of the lasing emission, which is influenced by both the intrinsic modal profiles and the radiation characteristics at each position (see Supplementary Section 10 for details). While they are not strictly identical to the underlying mode envelopes, they provide direct experimental access to the spatial variation of the mode. Moreover, the divergence angle of the lasing beam is extracted from the FFPs, serving as a robust and direct indicator of the slowly varying envelope wavevectors *q*_*x*,*y*_. By combining the real-space information from NFPs and the momentum-space information from FFPs, we can unambiguously identify the lasing mode in each sample and quantitatively track the spectral flow.

Specifically, we begin the tracking with sample U (*g* = 470 nm), designed for *θ* ~ *π*. The lasing mode is identified to be close to the $\left\vert 22^{\prime} \right\rangle$ mode, characterized by a relatively large divergence angle of 3. 6°. In its NFP, four dark spots (white dashed circles) are clearly observed, corresponding to the nodal structure of the modal envelope (Fig. 2c). Meanwhile, the FFP exhibits four distinct lobes with a central dark region, representing a typical far-field signature of $\left\vert 22^{\prime} \right\rangle$ mode when a *Γ*-point BIC exists^56,60^. As *g* is reduced to 450 nm (V) and 430 nm (W), the reflection phase increases beyond *π*. In the NFP, the dark nodes (white dashed circles) progressively shift toward the corners of the cavity region. Correspondingly, in the FFP, four separated lobes gradually merge into a donut-shaped pattern, and thus the divergence angle decreases to 3. 3^∘^ (V) and 3. 1^∘^ (W). This evolution indicates a continuous reduction of slowly varying wavevectors *q*_*x*,*y*_, and confirms that mode $\left\vert 22^{\prime} \right\rangle$ is transitioning towards $\left\vert 11\right\rangle$ as *g* decreases, corresponding to the blue branch in Fig. 2c. A complete transition to $\left\vert 11\right\rangle$ is expected for smaller *g*, although this regime is currently inaccessible due to fabrication limitations.

To further demonstrate the spectral flow toward the fundamental mode $\left\vert 00\right\rangle$ with a flat-top envelope, we consider another evolutionary branch (red line, Fig. 2c), corresponding to samples X-Z, where the reflection phase is tuned from 0 to *π*. First, the lasing mode of sample X (*g* = 565 nm) is identified as $\left\vert 11\right\rangle$ mode. The measured divergence angle is 2. 0^∘^, which is in good agreement with the theoretical value (2. 2^∘^) for $\left\vert 11\right\rangle$. Consistently, no dark node is observed in its NFP. When the gap is reduced to 490 nm (Y) and 474 nm (Z), the divergence angle decreases to 1. 5^∘^ (Y), and further to only 1. 2^∘^ (Z). This trend indicates a progressive reduction of the slowly varying wavevectors, suggesting that the $\left\vert 11\right\rangle$ mode is evolving toward the fundamental mode $\left\vert 00\right\rangle$. At the same time, pronounced side lobes emerge as *g* decreases, attributed to stronger bulk-boundary interface scattering. This tendency is also reflected in the NFP, where the emission within the bulk region becomes progressively smoother, while the interface region shows increased intensity.

We identify the lasing mode in sample Z (*g* = 474 nm) as a near-$\left\vert 00\right\rangle$ mode. We note that its NFP exhibits a dark region across the whole cavity due to the symmetry-protected BIC at the band edge of TM_*B*_. Specifically, as approaching the $\left\vert 00\right\rangle$ mode, the slowly varying wavevectors gradually decrease to zero and align with the band edge. As a result, radiation from the central bulk region is strongly suppressed due to the BIC, leading to a relatively low emission power. It is noteworthy that the darkness observed in the NFP does not indicate a vanishing modal energy, but instead reflects a uniform suppression of radiation across the cavity. In this case, the lasing emission is dominated by scattering at the bulk-boundary interfaces, arising from momentum mismatch when the flat-top envelope rapidly decays near the boundary. This interpretation is further supported by the FFP observation of sample Z: the donut-shaped pattern is preserved while the divergence angle reaches its minimum value, indicating that the slowly varying wavevectors *q*_*x*,*y*_ approach zero. Taken together, these observations provide strong evidence for the formation of a near-flat-top mode.

To provide a comprehensive view of spectral flow, we plot the measured divergence angles of all samples as a function of the spacing *g*, and map them onto the evolution trajectory (Fig. 4d). The calculated divergence angle for $\left\vert 11\right\rangle$ mode (2. 2^∘^, dashed line) at *θ* = 0 is annotated as a reference. As *g* decreases, the reflection phase *θ* increases from 0 to 2*π*, giving rise to two distinct evolution branches: $\left\vert 11\right\rangle \to \left\vert 00\right\rangle$ (samples X-Y-Z, red dotted line) and $\left\vert 22^{\prime} \right\rangle \to \left\vert 11\right\rangle$ (samples U-V-W, blue dotted line). The calculated divergence angle of $\left\vert 11\right\rangle$ mode serves as the asymptote between the two branches, agreeing well with our theoretical predictions (Fig. 2c). For sample Z with *θ* ~ *π*, the lasing mode approaches the fundamental mode $\left\vert 00\right\rangle$, exhibiting the smallest divergence angle observed in our experiments (1. 2^∘^), which is close to the expected limit for a near-flat-top mode under our fabrication constraints. We note that the experimentally accessible range of *g* differs from that in the 2D simulations shown in Fig. 2c. This discrepancy arises because the realistic 3D hetero-bonded geometry modifies the band structure, thereby altering the quantitative relation between *g* and the effective reflection phase *θ*.

**Fig. 5:**
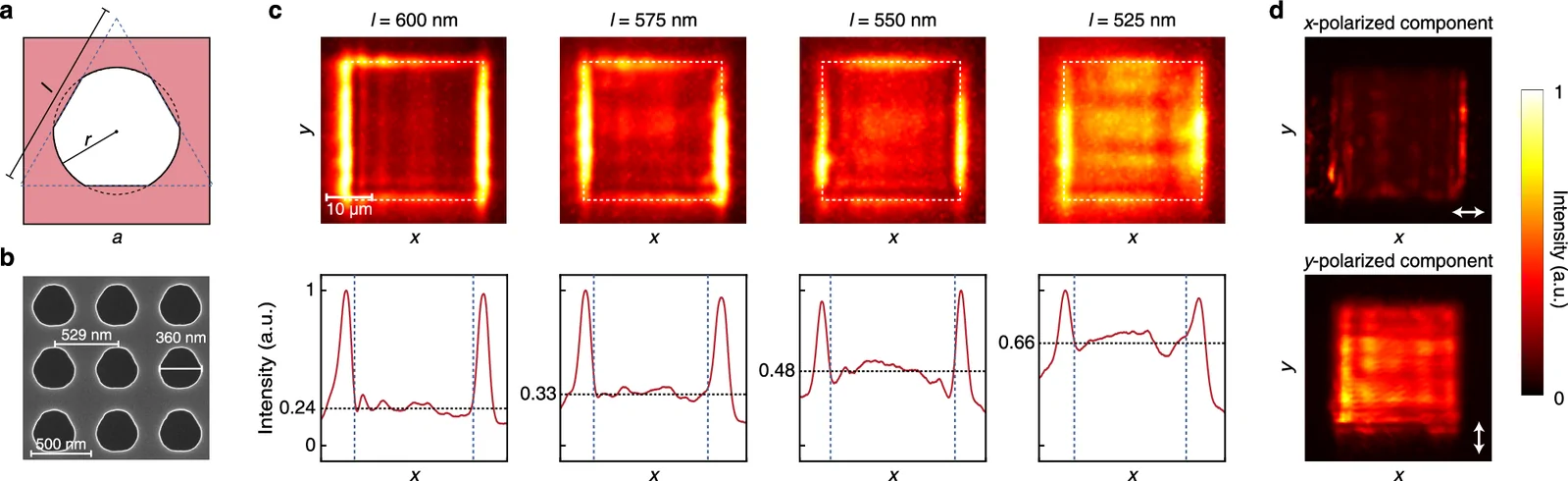
Near-flat-top modes in *C*_2_-symmetry-broken system. **a** Schematic of the irregularly-shaped air hole without *C*_2_ symmetry, designed as a circle of radius *r* cut by an equilateral triangle of side length *l*. **b** SEM image of the fabricated sample with designed irregularly-shaped air holes. **c** Top panels: Measured NFP for samples of different side lengths *l*; dashed boxes outline bulk-boundary interfaces. All NFPs are normalized with respect to the intensity of interface scattering. Bottom panels: Corresponding line-cuts along the *x* direction (integral over the *y* direction). As *C*_2_ symmetry breaking increases (*l* decreases), flat yet nonzero NFPs emerge, and the average intensity (horizontal dashed line) within the cavity region (between two vertical dashed lines) progressively increases. **d** Measured *x*- (top) and *y*-polarized (bottom panel) components of the lasing beam from the sample with *l* = 550 nm. The *x*-polarized component nearly vanishes while the *y*-polarized component exhibits a flat distribution.

A particularly intriguing feature of the flat-top fundamental mode $\left\vert 00\right\rangle$ is its ability to replicate the band-edge characteristics—generally considered a feature of the infinite bulk—throughout a finite domain. Beyond the case of a system hosting a BIC at the *Γ* point, as discussed above, we further demonstrate that this mechanism persists even in a *C*_2_-symmetry-broken system. Specifically, we consider a design with irregular air holes that break the in-plane *y*-mirror symmetry, formed by truncating a circle of radius *r* with an equilateral triangle of side length *l* (Fig. 5a). This in-plane asymmetry converts the ideal BIC into a quasi-BIC with finite vertical radiation near the band edge. Based on this design, we fabricate four samples (Fig. 5b) with decreasing *l*, thereby progressively enhancing the asymmetry. Despite the symmetry breaking, the quasi-BIC still supports a relatively high *Q*, enabling stable single-mode lasing in all samples (Supplementary Section 9).

We measure the NFP of the lasing modes for all samples with different side lengths *l* (top panels, Fig. 5c). These NFPs are normalized to the intensity of interface scattering, and the corresponding line-cuts along the *x* direction (integral over the *y* direction) are shown in the bottom panels. In contrast to the dark NFP observed for the near-$\left\vert 00\right\rangle$ mode in the *C*_4_-symmetric design (sample Z, Fig. 4), these samples exhibit flat yet nonzero NFPs due to the broken *C*_2_ symmetry. In this case, the measured NFPs directly reflect the underlying flat and uniform modal envelope, providing clear evidence for the formation of flat-top modes. As *l* decreases to promote radiation, the average intensity of NFP within the bulk region increases (bottom panels, Fig. 5c), leading to a comparable brightness with the interface scattering. Furthermore, as shown in Fig. 5d, we perform polarization-resolved NFP measurements on the sample with *l* = 550 nm, where residual interface scattering is suppressed by using a square-shaped iris to improve visibility. The *x*-polarized component (left panel) is nearly absent, while the *y*-polarized component (right panel) remains a pronounced and spatially uniform distribution across the cavity region. This observation confirms that, under the designed boundary reflection phases, the near-$\left\vert 00\right\rangle$ mode faithfully inherits the radiation characteristics of the TM_*B*_ band at the *Γ* point, namely, a pure linear polarization along the *y* direction, throughout the whole cavity region.

## Discussion

We suggest that global phase accumulation induced by an artificial gauge field can drive continuous spectral flow and reshape the spatial profiles of wave states, thereby offering a simple and general route to flat-top modes with uniform, nonzero envelopes, which we experimentally observed via single-mode lasing in an active PhC slab that is laterally confined by a photonic bandgap with engineered reflection phases. This wave morphing behavior arises from the geometric effect of the artificial gauge field rather than local structural details and is therefore largely robust against variations in bulk properties, including geometry, structural size, and group velocity. From a scientific viewpoint, this phase-engineering perspective provides a general strategy for tailoring wave envelopes across a wide class of wave systems, and can be extended to topologically nontrivial systems^66,67^. From a technological viewpoint, this mechanism enables band-edge properties, such as emission strength^68,69^, polarization^70^, chirality^71,72^ and directionality^73^, to be replicated over a finite yet, in principle, scalable region, thereby opening promising opportunities for applications from quantum emitters to lasers.

## Methods

### Numerical simulations

Numerical simulations are conducted using the finite-element method implemented in COMSOL Multiphysics. The band structure in the left panel of Fig. 2c is computed using a 2D unit cell with Floquet periodic boundary conditions applied on the sides. The evolution and corresponding field distributions of the energy levels shown in the right panel of Fig. 2c are obtained through 2D simulations of the heterogeneous PhC structure. Particular focus is placed on the $\left\vert 00\right\rangle$ and $\left\vert 11\right\rangle$ modes. The field distribution of the $\left\vert 00\right\rangle$ mode, detailed in Fig. 2d, f, is used to derive the phase profiles of *ϕ*_*R*,*L*_ via partial-wave decomposition (Fig. 2e). The divergence angle of the $\left\vert 11\right\rangle$ mode in Fig. 4d is determined by applying a Fourier transform to its field profile. The band structures and *Q* diagram in Fig. 3d are calculated using a 3D unit cell representing the hetero-bonded bulk region, incorporating Floquet periodic boundaries on the sidewalls and perfectly matched layers at the top and bottom surfaces.

### Sample fabrication

The hetero-bonded system comprises two parts: the SOI chip is diced into 10 × 10 mm pieces, consisting of a stack with a 340 nm Si layer, 2 μm SiO_2_, and a 625 μm Si substrate; the InP-epi chip is diced into 5 × 5 mm pieces, where the active region consists of four 7.5 nm compressively strained *In*_0.51_*Ga*_0.49_*As*_0.932_*P*_0.068_ quantum wells and five 12 nm lattice-matched *In*_0.75_*Ga*_0.25_*As*_0.558_*P*_0.442_ quantum barriers, sandwiched by undoped 30 nm *In*_0.75_*Ga*_0.25_*As*_0.25_*P*_0.75_ cladding layers, and the thickness of the InP substrate is 325 μm. The two parts are bonded via a plasma-assisted bonding process. Firstly, the PhC and VOCs (vertical out-gassing channels) structures are patterned on the SOI chip. During bonding, both the SOI and epitaxial chips undergo cleaning with organic and inorganic solvents to remove particles and contaminants. An *O*_2_ plasma activation step follows, generating hydroxyl-rich functional groups on the chip surfaces. The chips are then aligned and pre-bonded at 150 ^∘^C under a 30 N force for 45 min, followed by annealing at 250 ^∘^C under the same force and duration to enhance bonding strength. After bonding, the InP substrate is mechanically ground and polished to a thickness of approximately 35 μm. The remaining InP substrate is then chemically etched using a solution of citric acid, hydrochloric acid, and water in a 1g : 8ml : 4ml ratio. Finally, the sample undergoes organic cleaning to remove surface particles and is dried with a nitrogen gun.

### Measurement and data processing

We use a 1064-nm-pulsed laser with a repetition frequency of 10 kHz and a pulse duration of 2 ns, to pump our sample at room temperature. As shown in Fig. 3e, a 10× objective lens (IOPAMI137110X-NIR, Mitutoyo) is used to converge the pump light to a spot and also collect the lasing beam generated from the sample. The following lens L_1_ (*f* = 200 mm) is confocal with the objective lens, as well as L_2_ (*f* = 150 mm) and L_3_ (*f* = 300 mm), forming a 4*f* system. The near-field and far-field images are captured by an InGaAs infrared CMOS camera (IMX990, Sony) and undergo data smoothing with a window size of 9 pixels after deducting CMOS intrinsic noise and ambient background noise. Meanwhile, the lasing beam is focused into a monochromator with a 600 g/mm optical grating and an infrared array detector (IsoPlane SCT320 and NIRvana 640, Princeton Instruments).

### Reporting summary

Further information on research design is available in the Nature Portfolio Reporting Summary linked to this article.

## Supplementary information


Supplementary Information
Reporting Summary
Transparent Peer Review file


## Source data


Source Data


## Data Availability

All the data supporting the findings of this study are available in the main paper, its Supplementary Information, or Source Data file. All data are available from the corresponding authors upon request. Source data are provided with this paper.
