# Supplementary material for: Wave morphing towards the flat-top envelope in photonics systems driven by artificial gauge fields
Source: Nat Commun. 2026 Jul 29;17:9178. doi: 10.1038/s41467-026-76042-0 (PMC13522590; doi:10.1038/s41467-026-76042-0)
Supplement: Supplementary file 1 [file 41467_2026_76042_MOESM1_ESM.pdf]

Supplementary Information – Wave morphing towards the flat-top envelope in photonics systems driven by artificial gauge fields

Peishen Li[1,#], Xiaoyu Zhang[1,#], Feifan Wang[1], Ye Chen[1], Xuefan Yin[1,*], and Chao Peng[1,2,*]

[1]State Key Laboratory of Photonics and Communication, School of Electronics & Frontiers Science Center for Nano-optoelectronics, Peking University, Beijing 100871, China

[2]Peng Cheng Laboratory, Shenzhen 518055, China

[#]These authors contributed equally to this work.

[*]To whom correspondence should be addressed; E-mail Xuefan Yin (xuefan_yin@pku.edu.cn), Chao Peng (pengchao@pku.edu.cn).

Supplementary Note 1. Artificial ring model with phase kinks

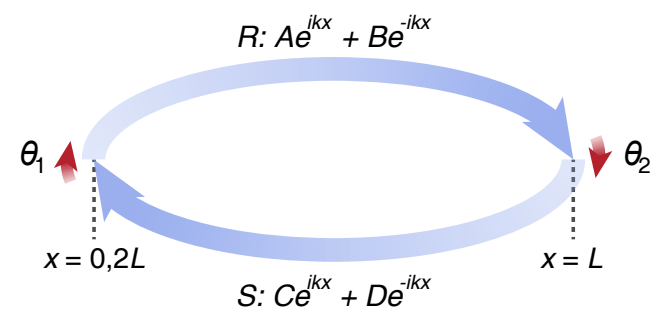

Supplementary Figure 1: **Artificial ring model with phase kinks.** Schematic of the ring model (divided into R and S) with two phase kinks serving as artificial gauge fields.

This section provides a detailed discussion upon the artificial ring model with phase kinks, shown in Fig. 1b in the main text. Without loss of generality, we take a ring (circumference $2L$) with two phase kinks (θ_1 and θ_2) as an example. These two kinks are located at $x = 0$ and $x = L$, respectively. Obviously, due to the phase kinks, the wavefunction of the eigenstate is no longer a continuous function in the ring. Instead, as shown in Supplementary Fig. 1, the kinks divide the whole ring into two arcs, labeled as R and S, connecting with each other tail to end. Upon each arc, the wavefunction is well-defined, taking a form as:

$$\begin{aligned} \psi_R(x) &= Ae^{ikx} + Be^{-ikx} \qquad x \in (0, L) \\ \psi_S(x) &= Ce^{ikx} + De^{-ikx} \qquad x \in (L, 2L) \end{aligned} \tag{1}$$

where $A,\ B,\ C,\ D$ are coefficients to be solved, and k is the wavevector. Note that $x = 0$ and $x = 2L$ refer to the same location on the ring. Right at the kinks where two arcs intersect, the boundary conditions can be derived as:

$$\begin{aligned} \psi_R(0_+) &= r_1 e^{i\theta_1}\psi_S(2L_-) \qquad \psi_R'(0_+) = r_1 e^{i\theta_1}\psi_S'(2L_-) \\ \psi_S(L_+) &= r_2 e^{i\theta_2}\psi_R(L_-) \qquad \psi_S'(L_+) = r_2 e^{i\theta_2}\psi_R'(L_-) \end{aligned} \tag{2}$$

where $r_{1,2}$ are real numbers representing the reflectivity. For Hermitian ring model, $|r_1| = |r_2| = 1$ holds in the following discussions. Combining Eq. 1 and Eq. 2, two quantization conditions can be solved:

$$\begin{aligned} r_1 r_2 e^{i(\theta_1+\theta_2)} e^{2ikL} &= 1 \\ r_1 r_2 e^{i(\theta_1+\theta_2)} e^{-2ikL} &= 1 \end{aligned} \tag{3}$$

These quantization conditions are only dependent upon the cumulative phase $\theta_{\text{tot}} = \theta_1 + \theta_2$, indicating that the specific distribution of the kinks upon the ring only acts as an artificial local gauge potential, and the cumulative phase acts as an effective magnetic flux. Further, the wavefunctions can be solved according to different quantization conditions as:

$$\begin{aligned} k_- &= \frac{2n\pi - \theta_{\text{tot}}}{2L} \qquad \psi_- = \begin{cases} \psi_{R-} = e^{ik_- x} & (0 < x < L) \\ \psi_{S-} = e^{i\theta_2} e^{ik_- x} & (L < x < 2L) \end{cases} \\ k_+ &= \frac{2n\pi + \theta_{\text{tot}}}{2L} \qquad \psi_+ = \begin{cases} \psi_{R+} = e^{-ik_+ x} & (0 < x < L) \\ \psi_{S+} = e^{i\theta_2} e^{-ik_+ x} & (L < x < 2L) \end{cases} \end{aligned} \tag{4}$$

in which $|r_1| = |r_2| = 1$ is applied. A relation between k_- and k_+ is derived as $k_{-,\pm n} = -k_{+,\mp n}$. Since energy E is proportional to k^2 ($E \propto k^2$), $k_{-,\pm n}$ and $k_{+,\mp n}$ exactly correspond to the same energy level E_n. In other words, when k is not zero, the eigenstates are doubly degenerate, propagating in either clockwise (k>0) or counter-clockwise ($k < 0$) direction.

Especially, when $\theta_{\text{tot}} = 2\pi$, both $k_{-,1}$ and $k_{+,-1}$ become zero, giving rise to the zero energy level. The wavefunction of zero energy can be derived as:

$$\psi_0 = \begin{cases} \psi_{R,0} = 1 & (0 < x < L) \\ \psi_{S,0} = e^{i\theta_2} & (L < x < 2L) \end{cases} \tag{5}$$

The eigenstate with zero energy is non-degenerate, whose wavefunction is constant in both arcs. Note that, although the energy levels as well as wavevectors are only dependent upon the total phase θ_{tot}, the wavefunction ψ_0 is associated with the phase kink θ_2. As a result, the energy levels are gauge-invariant — they are determined only by the artificial magnetic flux (θ_{tot}). At the same time, the wavefunctions are not gauge-invariant and are dependent upon the local gauge potentials (phase kinks distribution). By fixing the artificial magnetic flux (θ_{tot}) while adjusting the local gauge potential (θ_2), different wavefunctions corresponding to the same energy levels can be obtained, which is exactly the gauge transformation.

Supplementary Note 2. Homogeneous Neumann boundary conditions in potential well

Furthermore, we provide a detailed discussion of the effective homogeneous Neumann boundary conditions in the 1D potential well shown in Fig. 1c of the main text. First, we elaborate on the reflective boundaries in such a configuration. Specifically, the wavefunction inside the 1D potential well takes a simple form as:

$$\psi_{1D} = c_R e^{ikx} + c_L e^{-ikx} \tag{6}$$

where c_R and c_L denotes the amplitude coefficients of right- and left-propagating waves. Under proper gauge, the perfectly reflective boundaries are derived as:

$$c_R = -e^{i\theta_1} c_L \qquad\qquad c_L = -e^{i\theta_2} e^{2ikL} c_R \tag{7}$$

To derive the Neumann boundary condition, we consider the derivative of wavefunction ψ_{1D}:

$$\psi'_{1D} = ikc_R e^{ikx} - ikc_L e^{-ikx} \tag{8}$$

Then, substituting Eq.7 into Eq. 8, we have

$$\begin{aligned} \psi'_{1D}(0) &= ik(c_R - c_L) = -ik(1 + e^{i\theta_1})c_L = k\cot(\frac{\theta_1}{2})\psi_{1D}(0) \\ \psi'_{1D}(L) &= k\cot(\frac{\theta_1}{2} + kL)\psi_{1D}(L) \end{aligned} \tag{9}$$

The perfectly reflective boundary conditions are intrinsically general mixed boundary conditions which both involve $\psi_{1D}(0)$ and $\psi'_{1D}(0)$ (also $\psi_{1D}(L)$ and $\psi'_{1D}(L)$). Nevertheless, for zero energy level $k = 0$, mixed boundary conditions in Eq. 9 could be directly simplified to homogeneous Neumann boundary conditions $\psi'_{1D}(0) = \psi'_{1D}(L) = 0$, independent of the specific gauge θ_1. Moreover, the simplest special solution satisfying the homogeneous Neumann conditions is ψ^s_{1D} = const, indicating that at this time the wavefunction is completely uniform within the 1D potential well.

Supplementary Note 3. Continuous gauge transformation and effective potential

This section presents a complementary perspective on the formation of flat-top envelop. While boundary phase shifts offer an intuitive explanation for our experimental design, the flat-top envelope can also emerge in cavities without well-defined boundaries, motivating a more general gauge-based description. The analysis reveals a unified description based on an effective gauge potential that controls the relative phase between counter-propagating Bloch components.

Consider a 1D hetero photonic crystal cavity [Supplementary Fig. 2a], where the lattice constant is kept uniform throughout the structure, while a smooth spatial modulation of the hole radius r is introduced [Supplementary Fig. 2b]: in the cavity region, the hole radius symmetrically increases as $\delta r = \eta|x|^N$, followed by a sharp drop to form a photonic band gap. Here η denotes the modulation coefficient, integer N denotes the modulation order, and x is the spatial coordinate of each hole. This modulation induces an effective permittivity variation $\delta n(x)$, which continuously modifies the bulk mode profile. As shown in Supplementary Fig. 2c–e, different values of η lead to distinct envelope shapes: when $N = 1$, a Gaussian-like profile appears for $\eta = 0$, a flat-top-like envelope emerges at $\eta \approx 0.5$ nm/cell, and a boundary-enhanced profile is obtained at larger η (1 nm/cell). For $N = 2$, a flat-top-like envelope can also be realized by setting $\eta = 0.06$ nm/cell^2 [Supplementary Fig. 2f]. These results demonstrate that flat-top-like modes can arise from distributed permittivity modulation, without explicitly invoking boundary phase shifts.

To elucidate the physical origin of these flat-top-like modes, we analyze the transverse cavity mode near the Γ point, expressed as a superposition of counter-propagating Bloch com-

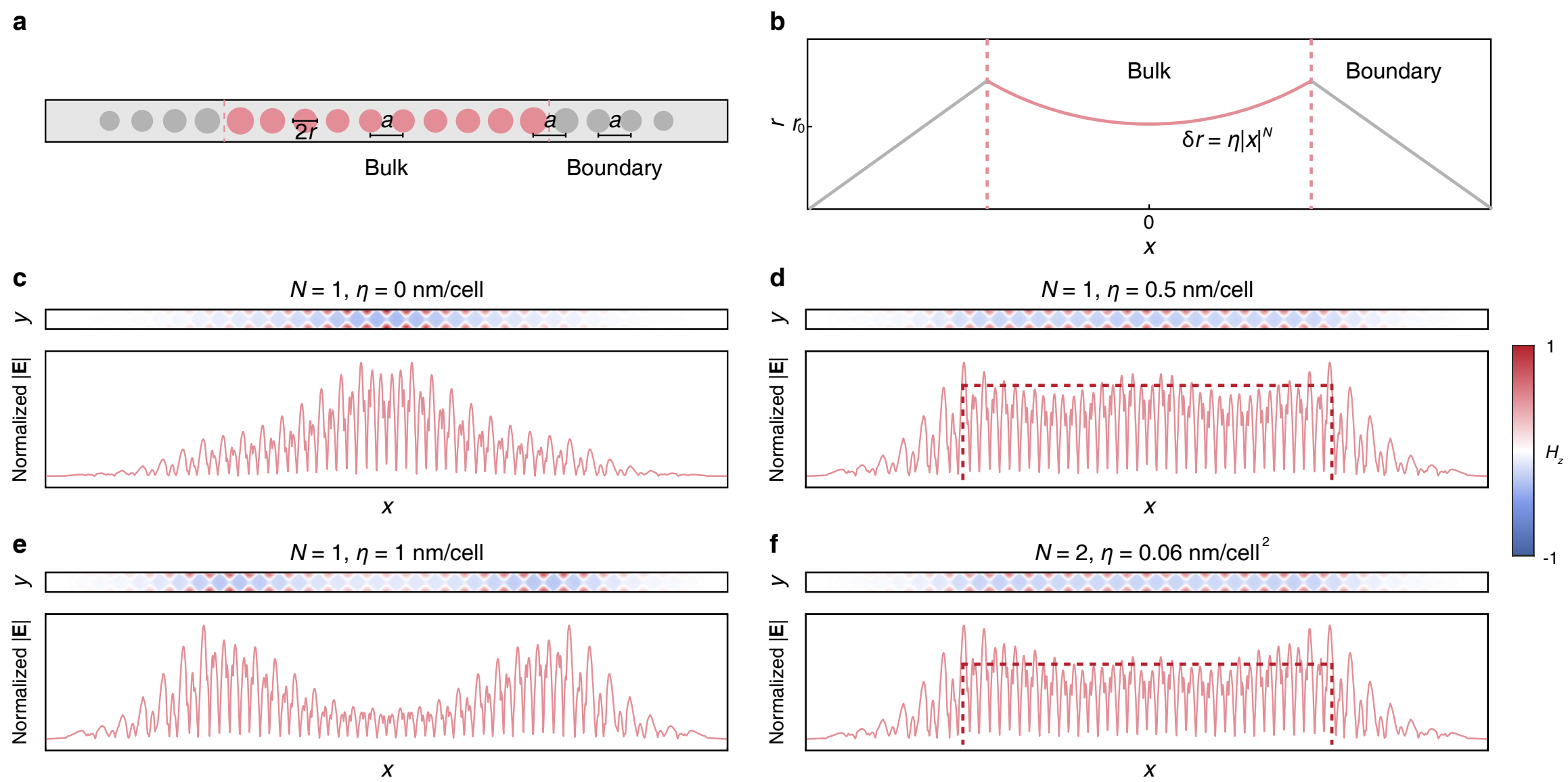

Supplementary Figure 2: **Generation of flat-top envelope via distributed spatial modulation of permittivity in a 1D hetero PhC cavity.** (a) Schematic of the cavity, with uniform crystal constant and modulated air-hole radius. (b) Spatial modulation function of hole radius r, showing a symmetric increase as $\delta r = \eta|x|^N$ followed by a sharp drop at the boundary region. (c–e) Mode envelopes for modulation order $N = 1$ with different modulation coefficients η: Gaussian-like profile ($\eta = 0$), flat-top-like profile ($\eta \approx 0.5$ nm/cell), and boundary-enhanced profile ($\eta = 1$ nm/cell). (f) Flat-top-like mode envelope achieved for $N = 2$ with $\eta = 0.06$ nm/cell^2.

ponents:

$$E = R(x)\mathrm{e}^{ikx} + S(x)\mathrm{e}^{-ikx} \tag{10}$$

where $R = |R|\mathrm{e}^{i\theta_R}$, $S = |S|\mathrm{e}^{i\theta_S}$, and $\phi = \theta_R - \theta_S$ is the relative phase. The weak spatial permittivity modulation $\delta n(x)$ introduced by gradually altering the hole radius gives rise to a position-dependent wavevector detuning $\delta k(x) \propto \delta n(x)$. The coupled-mode equations can then be derived as:

$$\frac{dR}{dx} = i\kappa S + i\delta k(x)R \qquad \frac{dS}{dx} = -i\kappa^\dagger R - i\delta k(x)S \tag{11}$$

where κ denotes the Bragg coupling originating from the distributed feedback of the lattice structure itself. Since the modulation is relatively weak, we assume κ is constant, and Eq. 11 can be rearranged as:

$$i\frac{d}{dx}\begin{pmatrix} R \\ S \end{pmatrix} = -[\mathrm{Re}[\kappa]\sigma_x - \mathrm{Im}[\kappa]\sigma_y + \delta k(x)\sigma_z]\begin{pmatrix} R \\ S \end{pmatrix} \tag{12}$$

In this form, the detuning $\delta k(x)$ plays the role of an effective "mass" term. By applying a gauge transformation:

$$R(x) \rightarrow \tilde{R}(x) = R(x)\mathrm{e}^{i\theta(x)} \qquad S(x) \rightarrow \tilde{S}(x) = S(x)\mathrm{e}^{-i\theta(x)} \tag{13}$$

and letting $\theta'(x) = \delta k(x)$, we obtain:

$$\frac{dR}{dx} = i\tilde{\kappa}S \qquad \frac{dS}{dx} = -i\tilde{\kappa}^\dagger R \tag{14}$$

where the detuning term is not eliminated, but rather transferred into a position-dependent phase of the coupling coefficient:

$$\tilde{\kappa} = \kappa\mathrm{e}^{-2i\theta(x)} = \kappa\mathrm{e}^{-2i\int \delta k(x)dx} \tag{15}$$

This transformation shows that the real-space modulation can be equivalently interpreted as a spatially varying phase in the coupling term, analogous to a Peierls phase in tight-binding

systems. In this sense, $\delta k(x)$ plays the role of an effective gauge potential, although no real magnetic field is involved.

According to Eq. 13, gauge transformation converts the spatial permittivity modulation into an additional phase difference between the two counter-propagating Bloch components:

$$\phi = 2kx + 2\int \delta k(x)dx \tag{16}$$

where the first term represents the fast oscillating dynamic phase and does not contribute to the slowly varying envelope. From this perspective, both boundary-induced phase shifts and distributed permittivity modulations can be viewed as different ways of engineering the same quantity: the relative phase between coupled Bloch components, which governs their interference and thereby determines the envelope profile.

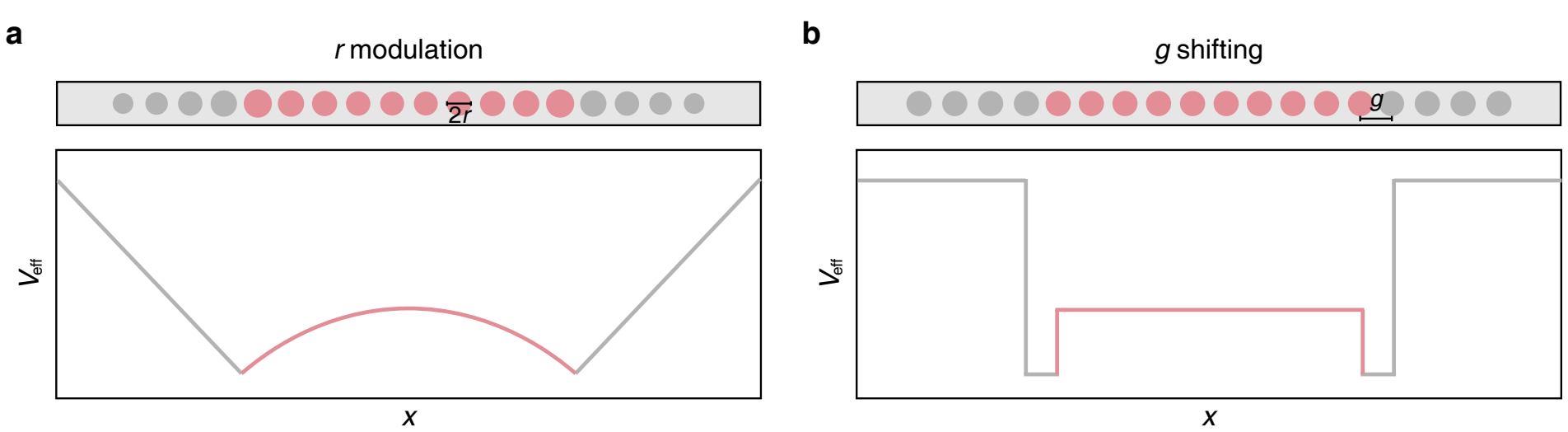

Supplementary Figure 3: **Effective gauge potential introduced by spatial engineering** with (a) r modulation and (b) g shifting (our experimental design).

As summarized in Supplementary Fig. 3, the gap g introduced in our design can be regarded as an attractive potential at the bulk-boundary interface, giving rise to a delta-like modulation $\delta k(x)$. While this implementation is not strictly adiabatic around the boundaries, it effectively produces the required phase shift in a compact form compatible with fabrication constraints. These examples suggest that the formation of flat-top envelopes does not rely on a specific implementation, but rather arises from a common physics: the spatial engineering of

phase gradients or effective momentum distributions. This unified phase-engineering perspective may also provide a general guideline for designing tailored wave envelopes in a wide class of photonic and wave systems.

Supplementary Note 4. Coupled-wave theory for homogeneous 2D PhC slab

To discuss the homogeneous PhC slab shown in Figs. 2 and 4 in the main text, we develop an analytical model by employing the coupled-wave theory (CWT) framework. According to the Bloch theorem, the bulk modes in the PhC slab can be expanded into a series of partial waves, denoted by indices (s, q):

$$E_{x,y}(x, y, z) = \sum_{sq} E^{sq}_{x,y}(z) e^{i(\mathbf{G}_{sq} - \mathbf{k}_{\|})\mathbf{r}} = \sum_{sq} E^{sq}_{x,y}(z) e^{is\beta_0 x + iq\beta_0 y} e^{-i\mathbf{k}_{\|}\mathbf{r}} \tag{17}$$

where $\mathbf{k}_{\|}$ is the in-plane Bloch vector related to the BZ center (Γ point). As we stated in the main text, for Bloch modes around the second-order Γ point, four partial waves dominate the modal energy, satisfying $s^2 + q^2 = 1$. Taking TE mode as an example, under transverse-field assumption, the components of these four partial waves take a form as:

$$\begin{aligned}
E^{1,0}_y(\mathbf{r}) &= \phi_R Z(z) e^{i\beta_0 x} \qquad E^{1,0}_x(\mathbf{r}) = 0 \\
E^{-1,0}_y(\mathbf{r}) &= \phi_L Z(z) e^{-i\beta_0 x} \qquad E^{-1,0}_x(\mathbf{r}) = 0 \\
E^{0,1}_x(\mathbf{r}) &= \phi_U Z(z) e^{i\beta_0 y} \qquad E^{0,1}_y(\mathbf{r}) = 0 \\
E^{0,-1}_x(\mathbf{r}) &= \phi_D Z(z) e^{-i\beta_0 y} \qquad E^{0,-1}_y(\mathbf{r}) = 0
\end{aligned} \tag{18}$$

Here $Z(z)$ is the out-of-plane profile in the 3D PhC structure. For the 2D PhC structure shown in Fig. 2 in the main text, we got $Z(z) \equiv 1$. The wavevector $\beta_0 = 2\pi/a$ describes the fast spatial oscillation part, corresponding to the Bloch band around the second Γ point. $\phi_{R,\ L,\ U,\ D}$ are complex amplitudes for the four partial waves. For an infinite PhC slab, the amplitudes are constant for every partial wave as ($\phi_{R,\ L,\ U,\ D} \equiv 1$). When a sufficiently large PhC is encircled by a heterogeneous PhC with a photonic bandgap, as discussed in the main text, the optical modes gain a slowly varying envelope in space.

Under the CWT framework, the coupling equations between the partial waves in heterogeneous PhC slab can be derived as:

$$\frac{\omega^2}{c^2}\begin{pmatrix}\phi_R\\ \phi_L\\ \phi_U\\ \phi_D\end{pmatrix} = \mathbf{C}\begin{pmatrix}\phi_R\\ \phi_L\\ \phi_U\\ \phi_D\end{pmatrix} + \frac{2i\beta_0}{\varepsilon_0}\begin{pmatrix}\frac{\partial}{\partial x}\phi_R\\ -\frac{\partial}{\partial x}\phi_L\\ \frac{\partial}{\partial y}\phi_U\\ -\frac{\partial}{\partial y}\phi_D\end{pmatrix} \tag{19}$$

where $\omega = 2\pi f$ is the frequency of the eigenstate; ε_0 denotes the average permittivity of the PhC slab; c denotes the speed of light in a vacuum. Matrix $\mathbf{C}$ refers to the coupling matrix in an infinite homogeneous PhC slab. Obviously, if the envelopes are constant, the spatial derivative terms in Eq. 19 vanish, and the equation can be simplified back to the homogeneous case.

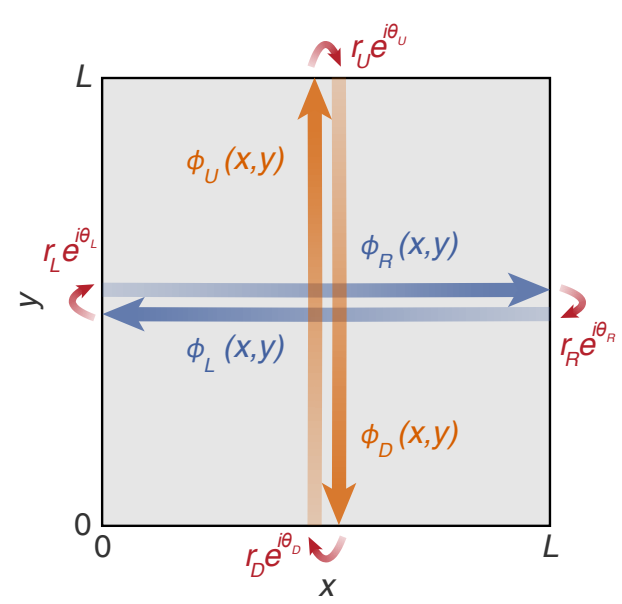

Supplementary Figure 4: **CWT model.** Reflective boundary conditions for partial waves.

For square boundaries shown in Supplementary Fig. 4, the reflective boundary conditions are as follows:

$$\begin{aligned}\phi_R(0,y) &= r_L e^{i\theta_L}\phi_L(0,y) \qquad & \phi_L(L,y) &= r_R e^{i\theta_R}\phi_R(L,y)\\ \phi_U(x,0) &= r_D e^{i\theta_D}\phi_D(x,0) \qquad & \phi_D(x,L) &= r_U e^{i\theta_U}\phi_U(x,L)\end{aligned} \tag{20}$$

where L is the size of the bulk region, and $L = Na$ is applied such that $e^{\pm\beta_0 L} = 1$ holds in both x and y directions. In this way, the boundary conditions have no relationship with the fast

oscillations. $r_{R,\ L,\ U,\ D}$ are absolute values of reflectivities upon four bulk-boundary interfaces, and $\theta_{R,\ L,\ U,\ D}$ are reflection phases accordingly. For the Hermitian case, $r_{R,\ L,\ U,\ D} = 1$ holds to ensure that no energy escapes from the central bulk region.

Next, we adopt an ansatz for the envelopes within the bulk PhC slab surrounded by the boundaries, which can be assumed as:

$$\begin{pmatrix} \phi_R \\ \phi_L \\ \phi_U \\ \phi_D \end{pmatrix} = \begin{pmatrix} V_{R,1} \\ V_{L,1} \\ V_{U,1} \\ V_{D,1} \end{pmatrix} c_1 e^{-iq_x x - iq_y y} + \begin{pmatrix} V_{R,2} \\ V_{L,2} \\ V_{U,2} \\ V_{D,2} \end{pmatrix} c_2 e^{iq_x x - iq_y y} + \begin{pmatrix} V_{R,3} \\ V_{L,3} \\ V_{U,3} \\ V_{D,3} \end{pmatrix} c_3 e^{-iq_x x + iq_y y} + \begin{pmatrix} V_{R,4} \\ V_{L,4} \\ V_{U,4} \\ V_{D,4} \end{pmatrix} c_4 e^{iq_x x + iq_y y} \tag{21}$$

in which $c_{1,\ 2,\ 3,\ 4}$ are superposition coefficients; vector $\mathbf{V}_j = [V_{R,j},\ V_{L,j},\ V_{U,j},\ V_{D,j}]^T$ is a solution to the perturbed Bloch bands:

$$\frac{\omega^2}{c^2}\mathbf{V}_j = (\mathbf{C} + \frac{2i\beta_0}{\varepsilon_0}\Delta_j)\mathbf{V}_j \tag{22}$$

with $\Delta_1 = \mathrm{diag}[-iq_x, iq_x, -iq_y, iq_y]$, $\Delta_2 = \mathrm{diag}[iq_x, -iq_x, -iq_y, iq_y]$, $\Delta_3 = \mathrm{diag}[-iq_x, iq_x, iq_y, -iq_y]$ and $\Delta_4 = \mathrm{diag}[iq_x, -iq_x, iq_y, -iq_y]$. Obviously, the eigenstates in finite-sized heterogeneous PhC slab can be considered as a superposition of several Bloch modes with different perturbations. These perturbations come from the last term in Eq. 19. Additionally, Ansatz solution in Eq. 21 indicates that under square-shaped boundaries, the 2D heterogeneous PhC can be decomposed into two 1D subsystems. For example, by letting $q_y = 0$, Eq. 21 can be simplified as:

$$\begin{pmatrix} \phi_R \\ \phi_L \end{pmatrix} = \begin{pmatrix} V_{R,1}c_1 + V_{R,3}c_3 \\ V_{L,1}c_1 + V_{L,3}c_3 \end{pmatrix} e^{-iq_x x} + \begin{pmatrix} V_{R,2}c_2 + V_{R,4}c_4 \\ V_{L,2}c_2 + V_{L,4}c_4 \end{pmatrix} e^{iq_x x} \tag{23}$$

which is exactly the expression stated in the main text ($c^{+}_{R,L} = \sum_{j=2,4} V_{R,L;j} c_j$ and $c^{-}_{R,L} = \sum_{j=1,3} V_{R,L;j} c_j$). In other words, ϕ_R and ϕ_L form a 1D subsystem governed by boundaries on the left and right. Similar analyses can be applied to $\phi_{U,\ D}$ by assuming $q_x = 0$.

Further, two quantization conditions can be derived by substituting Eq. 21 and Eq. 22 into boundary conditions (Eq. 20):

$$\begin{aligned} e^{2iq_xL} &= \frac{(V_{L,1} - r_R e^{i\theta_R} V_{R,1})(V_{R,2} - r_L e^{i\theta_L} V_{L,2})}{(V_{R,1} - r_L e^{i\theta_L} V_{L,1})(V_{L,2} - r_R e^{i\theta_R} V_{R,2})} = V_x(\theta_R, \theta_L) \\ e^{2iq_yL} &= \frac{(V_{D,2} - r_U e^{i\theta_U} V_{U,2})(V_{U,4} - r_D e^{i\theta_D} V_{D,4})}{(V_{U,2} - r_D e^{i\theta_D} V_{D,2})(V_{D,4} - r_U e^{i\theta_U} V_{U,4})} = V_y(\theta_U, \theta_D) \end{aligned} \tag{24}$$

Obviously, when $|r_{R,\ L,\ U,\ D}| = 1$, the absolute values of functions $V_{x,y}$ are always equal to 1. In this case, we let $\Theta_{x,y} = -\arg(V_{x,y})$, and two quantization equations can be derived:

$$2q_xL + \Theta_x(\theta_R, \theta_L) = 2m\pi \qquad 2q_yL + \Theta_y(\theta_U, \theta_D) = 2n\pi \tag{25}$$

which is exactly Eq. 1 in the main text.

Note that functions $V_{x,y}$ not only depend on the reflection phase $\theta_{x,y}$, but also are associated with the vectors $\mathbf{V}_j$. Comparing to quantization conditions in the simple ring model (Eq. 3), here the quantization conditions are modified by the Bloch bands. In fact, in a heterogeneous, finite-size PhC, it is the counter-propagating partial waves (i.e. $\phi_{R,L}$) that form a closed circulation which is topologically equivalent to a ring. As a superposition of partial waves, the Bloch mode itself acts as a “carrier wave" to support such an effective ring configuration. From this point of view, different energy levels determined from Eq. 25 correspond to different “signal" modulated onto the original Bloch mode, and the zero energy level exactly corresponds to the non-modulated one ($q_x = q_y = 0$) which aligns with the band-edge of original Bloch band.

Supplementary Note 5. Details of eigenstate $|11\rangle$

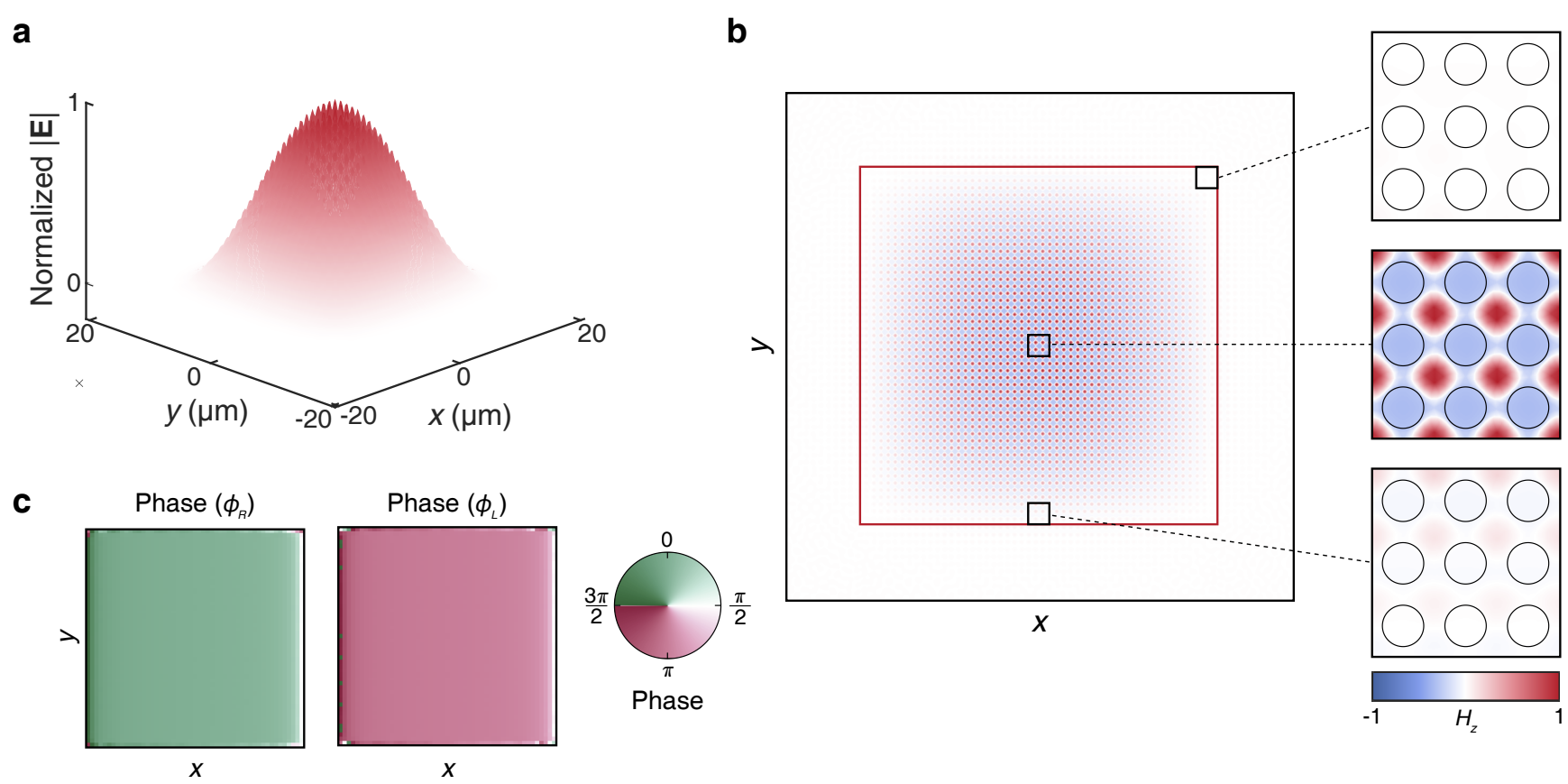

Supplementary Figure 5: **Details of the $|11\rangle$ mode.** (a) Envelope of the $|11\rangle$ mode. (b) Field distribution of the $|11\rangle$ mode in the entire PhC (left panel), with zoomed-in field distributions at its center, edge, and corner (right panels). (c) Phase distribution of partial waves $\phi_{R,\ L}$ for $|11\rangle$. All results are calculated by using numerical simulation (COMSOL Multiphysics).

In this section, we elaborate on the $|11\rangle$ mode as the candidate morphing into the nontrivial fundamental mode $|00\rangle$. Specifically, Supplementary Fig. 5 presents the modal characteristics from numerical simulations (COMSOL Multiphysics), in which the bulk-boundary gap is set to $g = 675$ nm. Supplementary Fig. 5a shows the normalized electric field distribution of the $|11\rangle$ mode, which exhibits a sinusoidal envelope with maximum intensity at the center, decaying toward the edges, and vanishing at the boundaries. Besides, Supplementary Fig. 5b plots the field distributions at selected locations. While the field remains strong and uniform in the central bulk region, it attenuates sharply near edges and corners, indicating strong confinement of $|11\rangle$.

Also, we check the phase distributions of partial waves within the bulk region. Similar to the main text, here we take $\phi_{R,L}$ of $|11\rangle$ as examples, whose phase distributions are presented in

Supplementary Fig. 5c. Note that the initial phase of the eigenstate itself can be arbitrary, and an arbitrary phase can be added onto $|11\rangle$. Nevertheless, the relative phase between two counter-propagating partial waves is independent on this initial phase. As shown in Supplementary Fig. 5c, under a proper choice of initial phase, in the central region, the phases of two partial waves remain close to 0 and π, respectively, showing a π difference. Then, these phases vary monotonically with x: one increasing (left panel for ϕ_R) and the other decreasing (right panel for ϕ_L), until they undergo rapid variation near the boundaries. Eventually, the two phases reach the same value on the left ($3\pi/2$) and right ($\pi/2$) boundaries, confirming a reflection phase of $\theta = \phi_R - \phi_L = 0$ for the $|11\rangle$ around the boundaries, consistent with the analysis in the main text.

The distinction in field distributions of $|11\rangle$ and $|00\rangle$ can be interpreted from a partial wave perspective. In both cases, the counter-propagating waves exhibit a π phase difference in the bulk. However, the $|11\rangle$ exhibits a reflection phase $\theta = 0$ on the boundaries. This phase mismatch impedes wave propagation from the bulk to the boundary, suppressing the mode at the boundaries and producing the observed sinusoidal envelope. In contrast, the $|00\rangle$ retains $\theta = \pi$ at the boundaries, matching the phase condition within the bulk and permitting unimpeded propagation, resulting in a uniform field profile. Although phase properties may vary across bands, the general approach of modal tailoring via boundary phase modulation remains broadly applicable.

Supplementary Note 6. Morphing of eigenstate field profiles

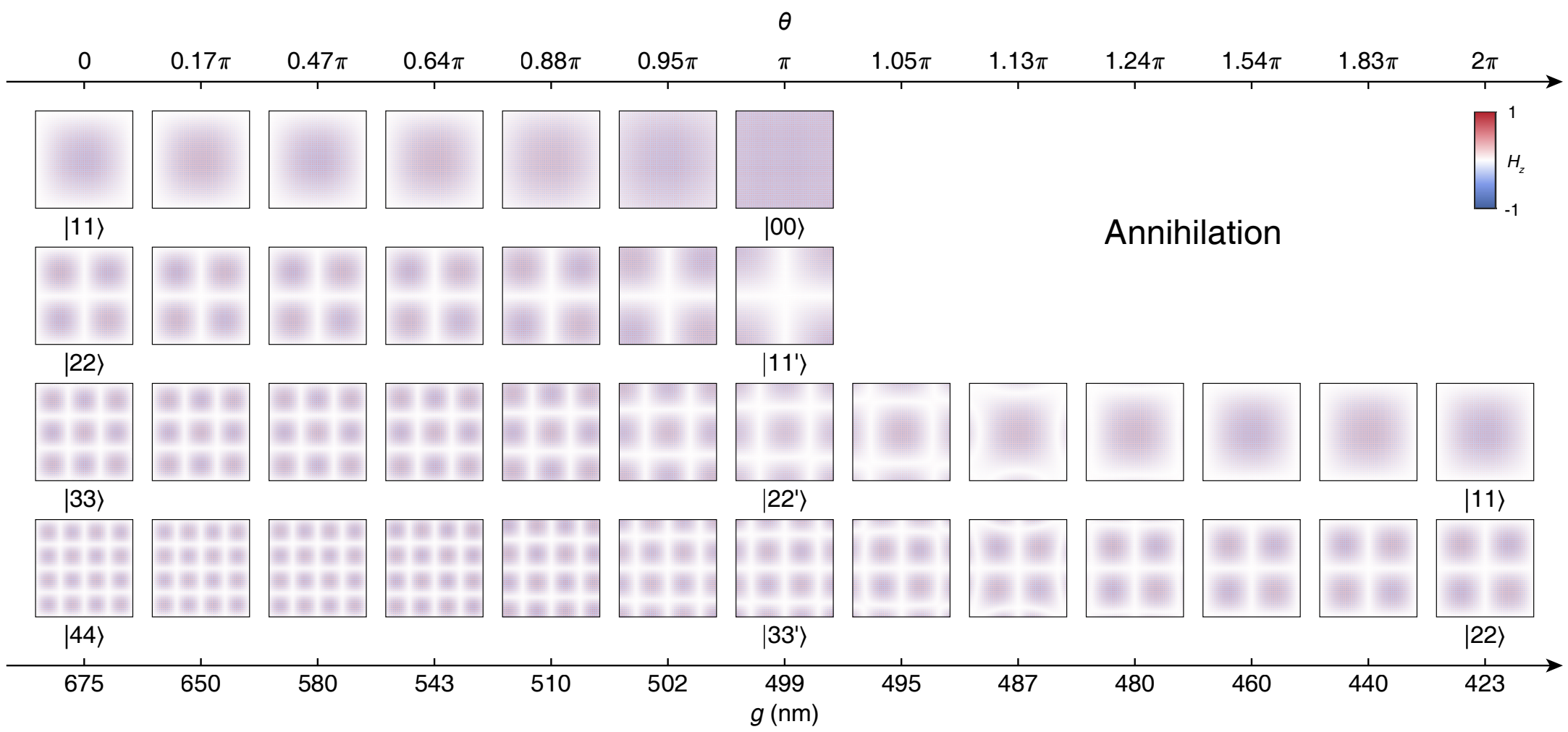

Supplementary Figure 6: **Field profile morphing during energy levels shifting.** The evolution of the H_z field distributions in the bulk region with respect to the reflection phase θ. As the spacing g is adjusted from 675 nm to 423 nm, θ correspondingly evolves continuously from 0 to 2π. The four rows represent the evolution from $|11\rangle$ to $|00\rangle$ (flat-top state) followed by annihilation, the evolution from $|22\rangle$ to $|11'\rangle$ followed by annihilation, the evolution from $|33\rangle$ to $|11\rangle$, and the evolution from $|44\rangle$ to $|22\rangle$, respectively. These evolutions correspond to the top-down four curves in the right panel of Fig. 2c. All results are calculated by using numerical simulation (COMSOL Multiphysics).

We further present the evolution of H_z field distributions as a function of reflection phases θ by varying the gap g (as summarized in Supplementary Fig. 6). Each row in Supplementary Fig. 6 corresponds to the continuous evolution of one energy level. Most data points are explicitly indicated in Fig. 2c. The reflection phase θ is calculated from the numerical simulation results combined with partial wave decomposition method. As we stated in the main

text, under square-shaped boundaries, wave morphing in x and y directions can be implemented independently. For clarity, here we restrict our discussion to modes $|mn\rangle$ with $m = n$.

At $g = 675$ nm, $\theta = 0$. The energy level $|nn\rangle$ exhibits an in-plane field envelope folded $(n-1)$ times ($n-1$ nodes) along both orthogonal directions, with vanishing field intensity at the boundary. As g decreases, θ increases from zero, leading to outward expansion of the modal profiles and the emergence of non-zero field intensity at the boundary. When $g = 499$ nm and $\theta = \pi$, the first energy level acquires a uniform spatial profile, corresponding to a flat-top mode defined as $|00\rangle$. For other energy levels, the field intensity reaches its maximum at the boundary. At this point, each such mode not only obtains an eigenfrequency originally belonging to the $(n-1)$-th level, but also exhibits a modal profile equivalent to a spatially shifted $|n-1, n-1\rangle$ mode, denoted as $|n-1, n-1'\rangle$ mode. In this way, a morphing from $|nn\rangle$ to $|n-1, n-1'\rangle$ is observed for every $n \geq 1$.

As g further decreases and θ exceeds π, higher-order modes ($n \geq 2$) continue expanding outward. For instance, the $|22'\rangle$ mode exhibits an enlarged central region and significantly attenuated peripheral lobes. At $g = 423$ nm and $\theta = 2\pi$, the outer lobes vanish completely, field intensity again reaches zero at the boundary, and the field profile converges to that of the standard $|11\rangle$ mode. This completes a transition from $|nn\rangle$ to $|n-2, n-2\rangle$ over one full cycle of θ from 0 to 2π. As for the first two energy levels $|00\rangle$ and $|11'\rangle$, as discussed in the next section, due to the non-Hermiticity brought by the boundaries, they would become confined around the boundaries and quickly undergo annihilation.

Notably, as g decreases below 423 nm, θ exceeds 2π and the evolution enters a subsequent cycle. Theoretically, such cyclic and continuous energy-level shifting can be repeated indefinitely through geometric modulation of boundary parameters.

Supplementary Note 7. Theoretical model of $\theta - g$ relationship

We present a simple theoretical model that reveals the connection between gap spacing g and reflection phase θ. The transverse modes exhibiting a flat-top profile near the second-order Γ point can be approximately described as a superposition of only two counter-propagating Bloch components, Re^{ikx} and Se^{-ikx}. Here $k \approx |G| = 2\pi/a$ because the wavevector associated with the slowly varying envelope vanishes for such flat-top modes. G denotes the reciprocal lattice vector and a is the lattice constant. Since these two components carry most of the mode energy, higher-order contributions are neglected for simplicity. The relative phase between the R and S components can then be written as

$$\theta_{\text{tot}} = \theta_R + \theta_D = \theta_R - 2GL = \theta_R - \frac{4\pi}{a}L \tag{26}$$

where θ_R is the phase shift directly induced by the photonic-band-gap reflection. Its value depends on a variety of factors, such as the width of the photonic band gap, the material permittivity, the group velocity of the band of interest, etc. For a given design in which only the bulk-boundary gap g is varied, θ_R can be regarded as a constant and simply serves as a bias.

The second term, $\theta_D = -2GL$, accounts for the dynamical phase accumulated during propagation. By writing $L = Na + 2g$, where N is the number of unit cells in the bulk region, the dynamical phase can be separated into two parts: $\theta_D = \theta_f + 2\theta_g$. The first part $\theta_f = -4N\pi$ attributes to the fast oscillation and does not affect the slowly varying envelope. The second part is two times of extra phase accumulated in an interface gap g:

$$\theta_g = -\frac{4\pi}{a}g \tag{27}$$

which represents an extra phase accumulated owing to the gap g. This contribution is crucial for engineering the boundary phase. By adjusting the gap by half a lattice constant, $\Delta g \approx a/2$,

an additional phase shift of $\Delta\theta_g = -2\pi$ can be achieved. In our design, the lattice constant is $a = 529$ nm. As shown in Supplementary Fig. 7, changing g from 675 nm to 423 nm, i.e., $\Delta g = 252$ nm $\approx a/2$, produces a boundary phase shift of $\Delta\theta = 2\pi$, which agrees well with the theoretical prediction.

By assuming θ_R is unchanged as g varies, we plot the theoretical prediction of θ with respect to g according to Eq.27, shown as a dashed line in Supplementary Fig. 7. From the simplified model, θ shows a linear dependence on g. However, in simulations dealing with a realistic cavity, $\theta(g)$ curve is not linear, but twists when θ approaches π. The distinction mainly comes from the fact that θ_R actually varies with respect to g. For example, when approaching to the flat-top condition ($\theta \approx \pi$), the slowly varying wave vector of the fundamental mode vanishes, corresponding to the band edge, and thus the group velocity also vanishes. As a result, the direct reflection θ_R changes accordingly.

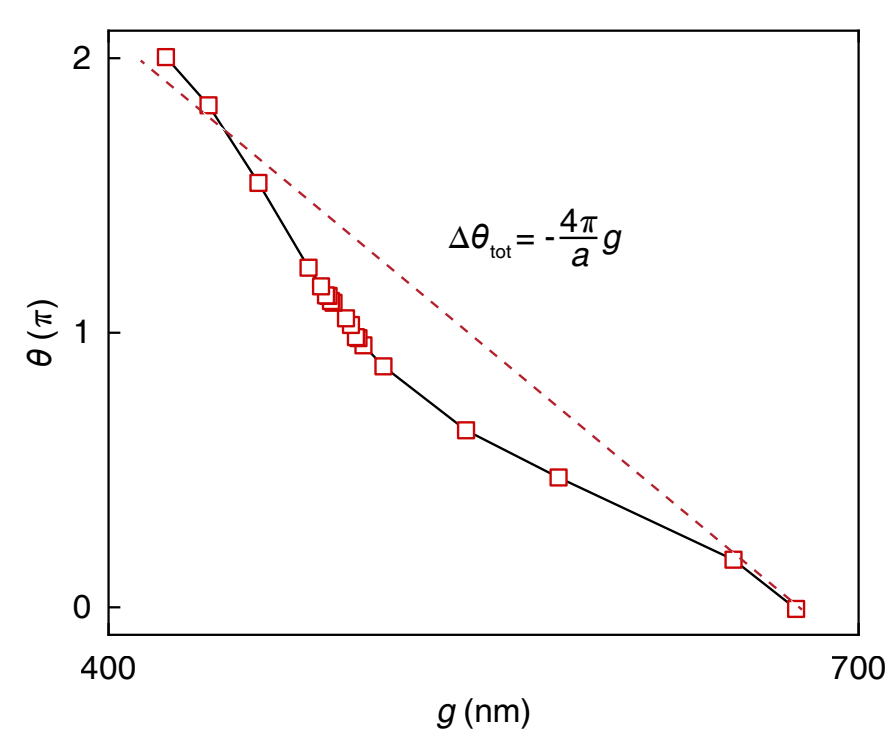

Supplementary Figure 7: **Reflection phase θ as a function of the spacing g.** The solid curve corresponds to simulation results, and the dashed line represents the theoretical prediction assuming θ_R remains unchanged as g varies.

Supplementary Note 8. Non-Hermitian ring model

In previous sections, we focused on the Hermitian case, where only phases change at perfectly reflective boundaries. Here, we consider the non-Hermitian case where the energy is allowed to escape to the surrounding environment.

First, we consider the non-Hermitian ring model. In Eq. 2 and Eq. 3, when $|r_{1,2}| \neq 1$, a complex k should be considered: $k = k_r + ik_i$. Consequently, Eq. 3 can be rewritten as:

$$
\begin{aligned}
r_1 r_2 e^{i(\theta_1+\theta_2)} e^{2i(k_r+ik_i)L} = 1 \quad &\rightarrow \quad \begin{cases} 2k_r L + \theta_1 + \theta_2 = 2n\pi \\ r_1 r_2 = e^{2k_i L} \end{cases} \\
r_1 r_2 e^{i(\theta_1+\theta_2)} e^{-2i(k_r+ik_i)L} = 1 \quad &\rightarrow \quad \begin{cases} -2k_r L + \theta_1 + \theta_2 = 2n\pi \\ r_1 r_2 = e^{-2k_i L} \end{cases}
\end{aligned} \tag{28}
$$

Obviously, the original two-fold degeneracy is lifted: the counter-propagating modes would suffer either effective gain ($k_i > 0$) or loss ($k_i < 0$). By choosing a proper gauge, k_r can still be zero, indicating that the wave no longer oscillates. However, a nonzero k_i implies that the wave can still grow or decay along the ring. At this time, an energy level even lower than the zero level can be estimated: $E \propto k^2 = (ik_i)^2 = -|k_i|^2 < 0$, indicating that energy can escape from the ring.

Then, we turn to the 1D potential well with imperfectly reflective boundaries ($|r_{R,L}| < 1$). Similarly, the quantization conditions should be revised with a complex $k = k_r + ik_i$ as:

$$
r_R r_L e^{i(\theta_1+\theta_2)} e^{2ikL} = 1 \quad \rightarrow \quad \begin{cases} 2k_r L + \theta_1 + \theta_2 = 2n\pi \\ r_R r_L = e^{2k_i L} \end{cases} \tag{29}
$$

Since we have $|r_{R,L}| < 1$, a negative $k_i = -|k_i| < 0$ can be readily derived. Then, as shown in Supplementary Fig. 8a, for right-propagating wave $c_R e^{ikx} = c_R e^{ik_r x} e^{|k_i| x}$, a growing envelope is expected along the x direction. While a decaying envelope is obtained for left-propagating wave $c_L e^{-ikx} = c_L e^{-ik_r x} e^{-|k_i| x}$. In other words, when energy is allowed to escape from the central bulk region into the boundary, the waves tend to be localized around the boundaries. This phenomenon is quite similar to the non-Hermitian skin effect in the topological SSH chain, where Bloch wavevectors become complex due to non-Hermiticity.

A similar phenomenon can be realized in a heterogeneous PhC structure. As shown in Supplementary Fig. 8b, when decreasing g from 499 nm to 496 nm, the boundaries become lossy for $|00\rangle$, transitioning it from the zero-energy state to the special $|\varepsilon\rangle$ state with energy "lower" (frequency higher) than the bulk band edge (also in Fig. 2c in the main text). The NFP and FFP of this $|\varepsilon\rangle$ state are shown in Supplementary Fig. 8c,d. Obviously, the field envelope of $|\varepsilon\rangle$ is not flat anymore, but localized around the bulk-boundary interfaces and corners. Accordingly, the central main lobe in FFP becomes even smaller than that of $|00\rangle$, and the side lobes become much brighter than the main lobe.

It is crucial to distinguish the boundary-localized behavior described above from the conventional non-Hermitian skin effect (NHSE). The NHSE originates from non-reciprocal hopping amplitudes and accumulates all bulk modes at one boundary, even without external leakage. In our system, the boundary-enhanced localization of the ($|\varepsilon\rangle$) state arises from leakage-induced complex wavevectors ($k = k_r + ik_i$), which produce a reciprocal gain/loss-type envelope rather than any non-reciprocal pumping. The flat-top fundamental state $|00\rangle$, by contrast, appears in the Hermitian limit of the ring model: a geometric phase serves as an artificial gauge potential, inducing a gauge-field-induced spectral flow that, together with the effective Neu-

mann boundary conditions, yields a vanishing envelope wavevector and a stationary uniform field distribution. Hence, the flat-top state is fundamentally distinct from both the leakage-induced boundary-enhanced states and the non-Hermitian skin modes — it originates from gauge-field engineering and boundary-condition design, not from non-reciprocity.

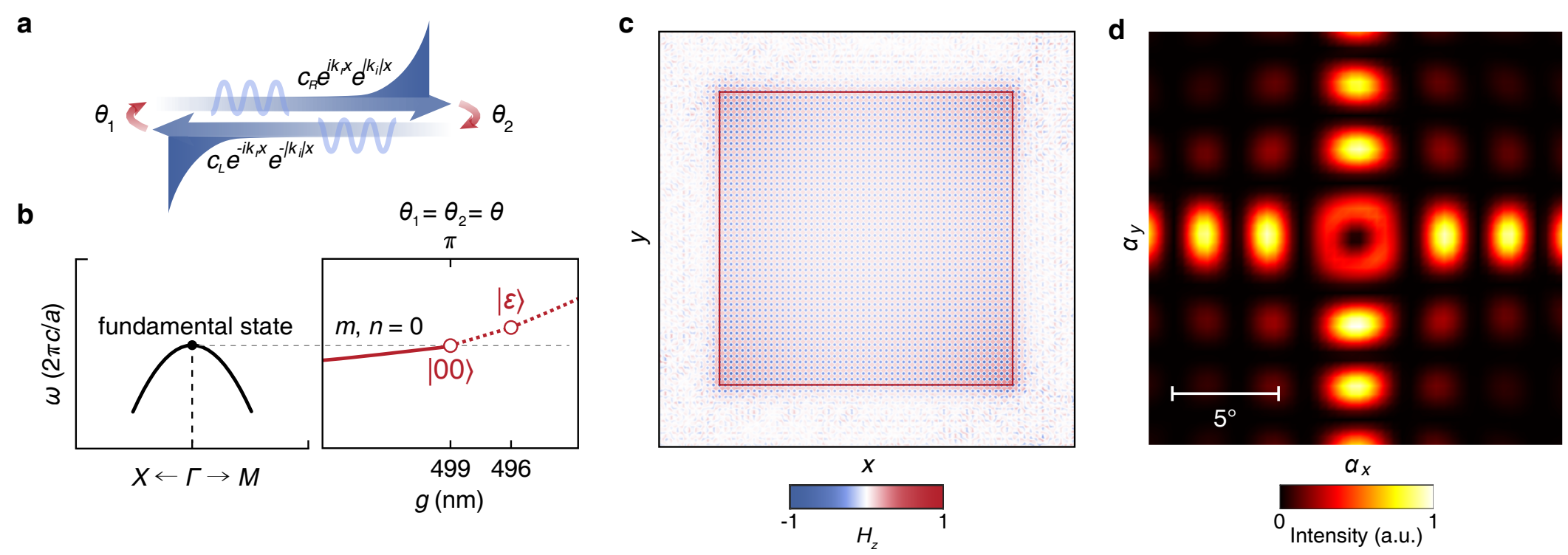

Supplementary Figure 8: **Non-Hermitian ring model and realization in PhC structure.** (a) Schematic of the Non-Hermitian ring model with waves localized around the boundaries. (b) Left panel: band structure with a gray dashed line indicating the band edge and the fundamental state with $m = n = 0$. Right panel: transition from the zero-energy state $|00\rangle$ to $|\varepsilon\rangle$ when decreasing g from 499 nm to 496 nm. (c) Field distributions of $|\varepsilon\rangle$ state. (d) Far-field pattern of $|\varepsilon\rangle$ state. All results are calculated by using numerical simulation (COMSOL Multiphysics).

Supplementary Note 9. Design of PhC for lasing

In C_2-symmetric systems, BICs arise at the Γ point with theoretically diverging Q factors, but in practice their performance is limited by a rapid Q reduction with wavevector deviation, especially in compact devices. Off-Γ BICs, protected by $C_2^z T$ symmetry and characterized by quantized winding of the far-field polarization vector, can be tuned geometrically to shift toward the symmetry-protected BIC at Γ. As they merge, the radiative Q factor becomes far less sensitive to momentum-space perturbations, greatly enhancing the feasibility of high-Q resonances in finite-sized structures.

However, the heterogeneous bonding process naturally introduces vertical asymmetry to the stack, which strongly impedes the formation of merging BICs. To counter this, we exploited the comparable effective indices of the III–V epitaxial layers (150 nm, $n \approx 3.28$) and the Si layer (340 nm, $n = 3.48$) by applying a shallow etch of $h = 190$ nm into the Si layer during PhC fabrication. The nearly equal residual thicknesses in both materials restore approximate vertical symmetry and effectively satisfy $C_2^z T$ symmetry — thereby enabling a quasi-merging BIC. As shown in Supplementary Fig. 9a, the calculated Q-factor map of the TM_B band exhibits a maximum at the Γ point, consistent with in-plane C_2 symmetry, as well as high-Q ridges extending along Γ–M. This feature evidences a quasi-merging BIC near Γ, which broadens the momentum-space region sustaining large Q factors. Such a shallow-etched design significantly boosts the Q factor in finite-size PhC structures, reduces lasing thresholds, and enables stable single-mode lasing. Note that the gain profile in such a quasi-$C_2^z T$ symmetry structure remains asymmetric, as only the III/V epitaxial layers provide optical gain. Consequently, the TM_B mode still exhibits relatively strong in-plane electric components $E_{x,\,y}$ within the active layers to ensure sufficient excitation.

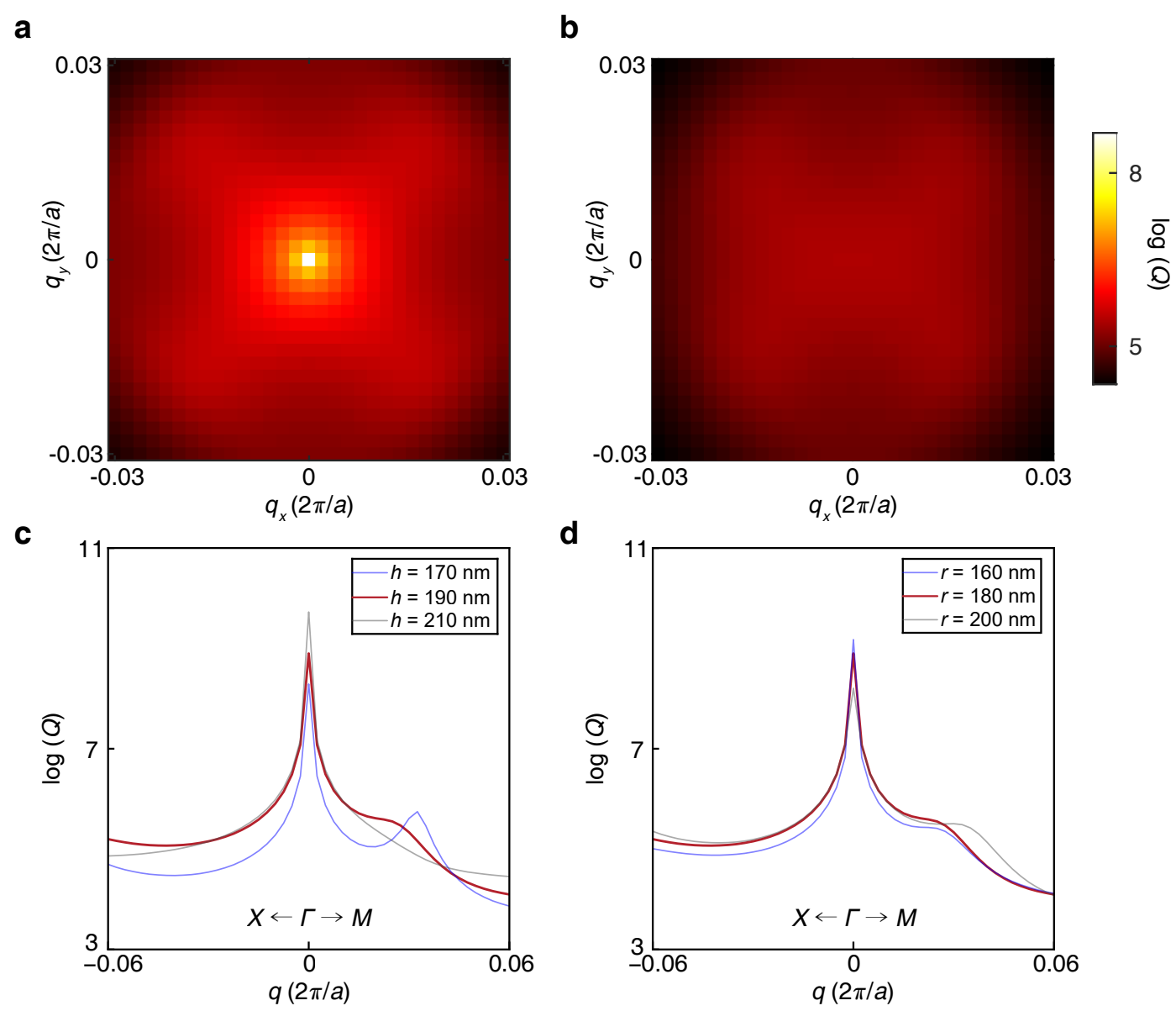

Supplementary Figure 9: ***Q*s of hetero-bond slabs.** (a)(b) Qs of the TM_B band of hetero-bond PhC slabs with (a) circular holes and (b) irregular-shaped holes with l = 550 nm. (c)(d) Comparison of Qs of the TM_B band between shallow-etched circular holes with (c) etching depth h varied around 190 nm and (d) radius r varied around 180 nm. All results are calculated by using numerical simulation (COMSOL Multiphysics).

The lasing mode is selected primarily by the quality factor Q and the spatial overlap Γ_{active} with the gain region, with the mode possessing the highest Q and largest Γ_{active} tending to prevail. In our cavities, the total Q of each transverse mode comprises both out-of-plane radiative loss and non-radiative losses from side leakage, random scattering, and material absorption. For a given set of transverse modes, the non-radiative contribution is expected to be comparable, as it is largely governed by fabrication imperfections and intrinsic material properties. Furthermore, the optical pump spot is deliberately chosen to be sufficiently large to cover the entire cavity, ensuring that all transverse modes experience a nearly identical gain distribution and thus a similar Γ_{active}. Consequently, differences in the radiative Q become the dominant factor in determining the lasing mode.

On the first energy band, a BIC with infinite radiative Q at the Γ point endows the lowest-order transverse modes with the highest Qs. As shown in Fig. 2c, when the boundary phase shift $\theta < \pi$, the modes on the $|11\rangle \rightarrow |00\rangle$ trajectory possess the highest radiative Qs and therefore win the lasing competition; for samples X-Z, which lie on this trajectory, the lasing mode belongs to this lowest branch. When $\theta > \pi$, the original $|11\rangle$ and $|22\rangle$ evolve into modes localized at the bulk–boundary interfaces and corners, suffering severe energy leakage and rapid annihilation. Instead, the original $|33\rangle$ mode (relabeled $|22'\rangle$) gradually evolves into a newborn $|11\rangle$ mode and retains the highest Q, thereby dominating the lasing action for samples U-W on the latter part of the $|33\rangle \rightarrow |11\rangle$ branch. In this manner, for every sample from U to Z, the lasing mode is consistently the one with the highest radiative Q among all supported modes under the respective geometric parameters, in full agreement with experimental observations. As shown in Supplementary Fig. 10, the lasing spectra of samples U through Z exhibit stable single-mode operation over a broad wavelength range of approximately 200 nm (from 1450 nm to 1650 nm).

To demonstrate the flat-top envelop, we modified the geometry of the cylindrical air holes, as illustrated in Fig. 5a of the main text. By cutting the circular holes with equilateral triangles, we deliberately broke the C_2 symmetry. This lifts the symmetry-protected BIC at the Γ point, causing its integer topological charge to split into a pair of half-integer charges carried by oppositely circular polarization states. As shown in Supplementary Fig. 9b, the Q distribution no longer exhibits a sharp peak at the Γ point, but instead remains uniformly high across a broad region of the Brillouin zone. Such a symmetry-broken design not only opens a radiation channel at the Γ point — allowing direct observation of the flat-top state — but also preserves a low lasing threshold and high beam quality. As demonstrated in Supplementary Fig. 11, the samples with broken C_2 symmetry (Fig. 5 of the main text) still support stable single-mode lasing, confirming that the design remains compatible with practical laser operation.

Finally, we assessed the robustness of the heterogeneous PhC slab by introducing geometric variations within typical fabrication tolerances. The etch depth h and hole radius r were independently varied by ± 20 nm from their nominal values to emulate practical errors. The resulting Q-factor distributions (blue and gray curves) compared with the original design (red curves) in Supplementary Fig. 9c,d show that such deviations cause no severe degradation of optical performance or lasing characteristics. This robustness arises from the topological protection of the quasi-merging BIC, while the results also confirm that the nominal design lies at a local optimum within the explored parameter space.

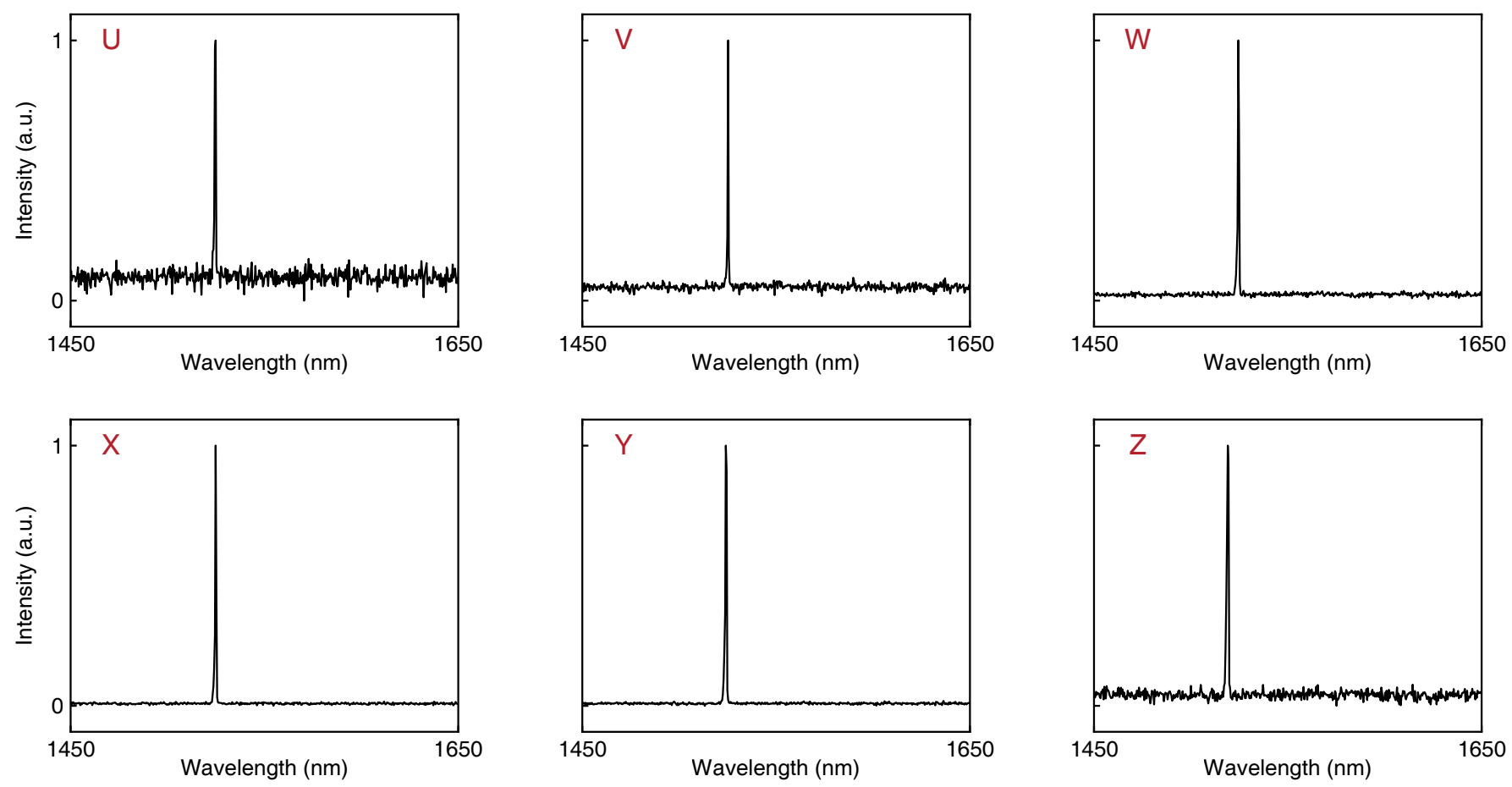

Supplementary Figure 10: **Measured lasing spectra for sample X through Z with circular holes.**

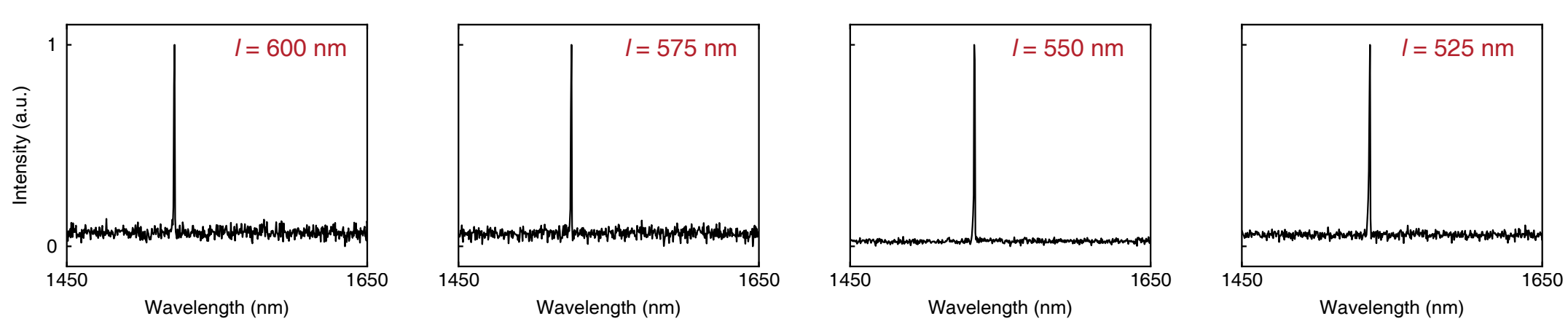

Supplementary Figure 11: **Measured lasing spectra for samples with irregular-shaped air holes.**

Supplementary Note 10. Interpreting near-field patterns and realizing uniform out-of-plane emission

In the main text, we observed that as the system approaches the $|00\rangle$ state, the NFP of sample Z exhibits a dark central region, with emission mainly arising from the bulk-boundary interfaces (Fig. 4c). To properly interpret this observation, it is essential to distinguish between the modal envelope profile (MEP) and the near-field pattern (NFP). The MEP represents the slowly varying envelope of the full modal field distribution, whereas the NFP corresponds to the out-of-plane emission imaged at the upper surface, determined by both the MEP and the local radiative rate.

In a photonic-crystal slab, the electric field can be expressed as:

$$E(\mathbf{r}) = F(x, y) \sum_{m,n} E_{m,n}(z) e^{iG_m x + iG_n y} \tag{30}$$

where $F(x, y)$ is the in-plane slowly varying envelope, and $G_{m,n}$ are reciprocal vectors. Only components with $|G_{m,n}| < \omega/c$ radiate to the far field and form the NFP; the remaining components are evanescent and do not contribute. By removing the fast-oscillating part, we can extract $F(x, y)$, as shown in Fig. 2d in the main text. For the $|00\rangle$, the NFP can thus be expressed as $\gamma_\Gamma F(x, y)$, where γ_Γ is the radiative rate at the Γ point.

In our C_2-symmetric design with circular air holes, a symmetry-protected BIC exists at the Γ point, eliminating the radiation ($\gamma_\Gamma = 0$). Consequently, the central cavity region appears dark in the NFP, even though the modal envelope $F(x, y)$ remains flat and non-zero. The darkness

originates from the vanishing band-edge radiation, not from a zero envelope.

The interface scatterings observed in the NFP arise from momentum mismatch between the bulk and boundary PhC regions, due to the finite gap g introduced for phase accumulation. Because the flat envelope maintains a finite field amplitude near the interface, these scatterings become pronounced when the bulk radiation is suppressed. Importantly, the scatterings do not indicate that the modal field is localized at the interfaces, nor do they affect the in-plane phase engineering or the formation of the $|00\rangle$ mode. This is directly verified by a simplified 3D cavity with circular holes optimized for the $|00\rangle$ (Supplementary Fig. 12a): the MEP (bottom panel) is flat-top-like, whereas the corresponding NFP shows a dark central region together with interface scatterings, confirming that NFP and MEP are fundamentally different.

To render the flat-top state directly observable as a bright near-flat NFP, we explore two methods that leave the modal flatness intact.

The first method is to make the central region brighter by breaking C_2 symmetry, thereby converting the BIC into a quasi-BIC with a finite γ_Γ. We introduce irregular-shaped air holes with asymmetry parameter l (smaller l corresponds to stronger symmetry breaking). For each l, the gap g is tuned to maintain the $|00\rangle$ with a flat-top envelope. The simulated out-of-plane emission patterns in Supplementary Figs. 12b–e show that as l decreases, the bulk region becomes progressively brighter and eventually comparable to the interface scatterings, yielding a near-flat NFP. This trend is in good agreement with the experimental NFPs presented in Fig. 5 of the main text.

The second method is to suppress interface scatterings by replacing the boundary PhC with a metallic mirror. A metal mirror provides a hard boundary that eliminates momentum

mismatch, and the reflection phase can be tuned by adjusting the distance between the PhC and the metal. However, fabricating a high-quality metallic side mirror with precise alignment and low optical loss remains challenging, and we have not pursued this approach experimentally in the present work.

Together, these two methods demonstrate that the flat-top state can be engineered to produce a uniform out-of-plane emission pattern, enabling scalable high-uniformity photonic devices.

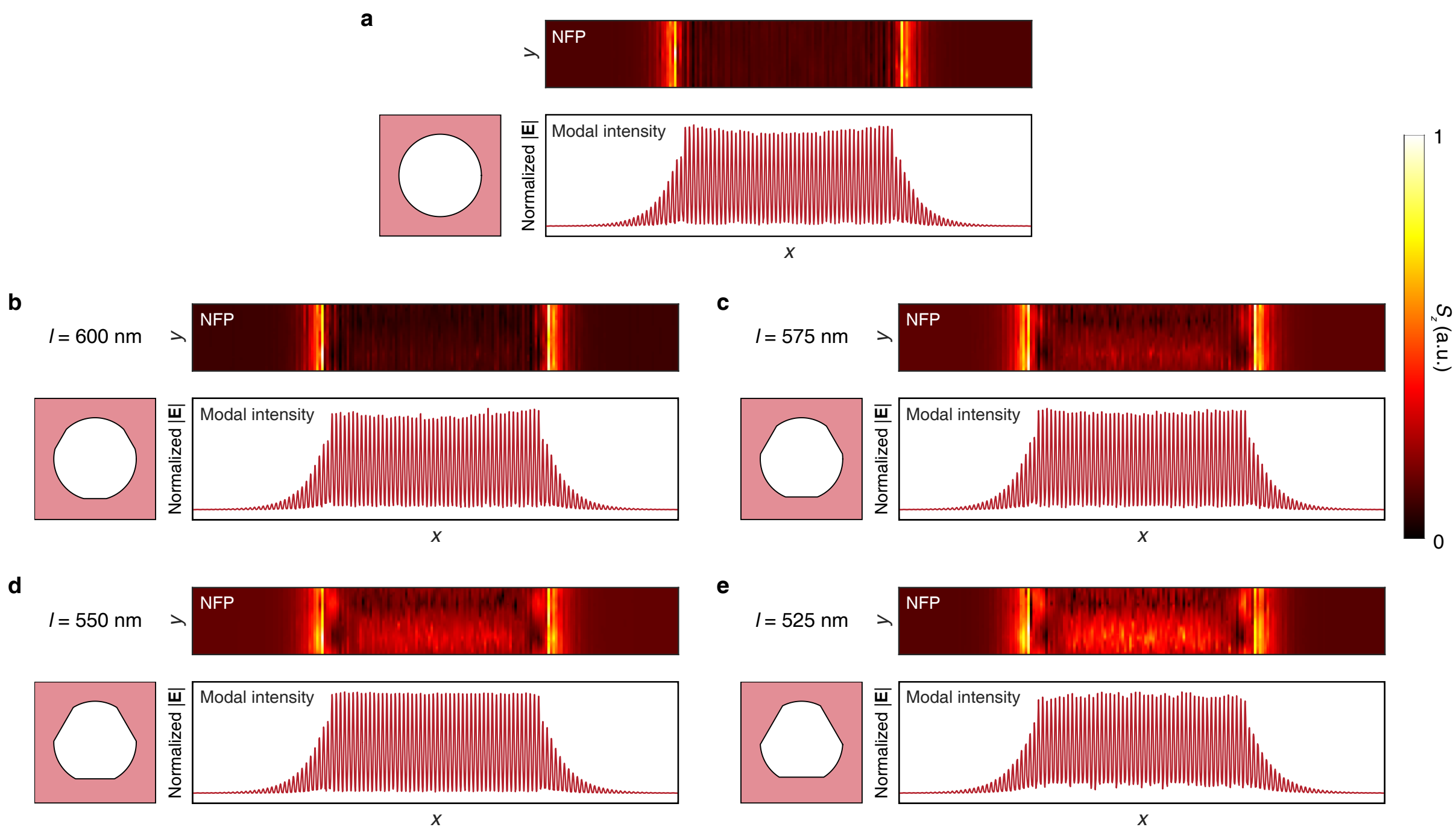

Supplementary Figure 12: **Achieving a bright flat NFP by increasing the degree of C_2-symmetry breaking.** Numerically simulated near-|00⟩ states with (a) circular holes and (b)-(e) irregularly-shaped air holes with different values of the parameter l (defined in Fig. 5a). Left panel: accurate structure diagram of the air holes. Upper right panel: magnitude of the z-component of the Poynting vector (S_z) above the top surface of the |00⟩ mode. Lower right panel: field distribution of the |00⟩ mode. To simplify the calculation, a bar-shaped structure extended along the x direction is used, with only one unit cell in the y direction and periodic boundary conditions applied.

Supplementary Note 11. Lasing characteristics of the $|11\rangle$ mode

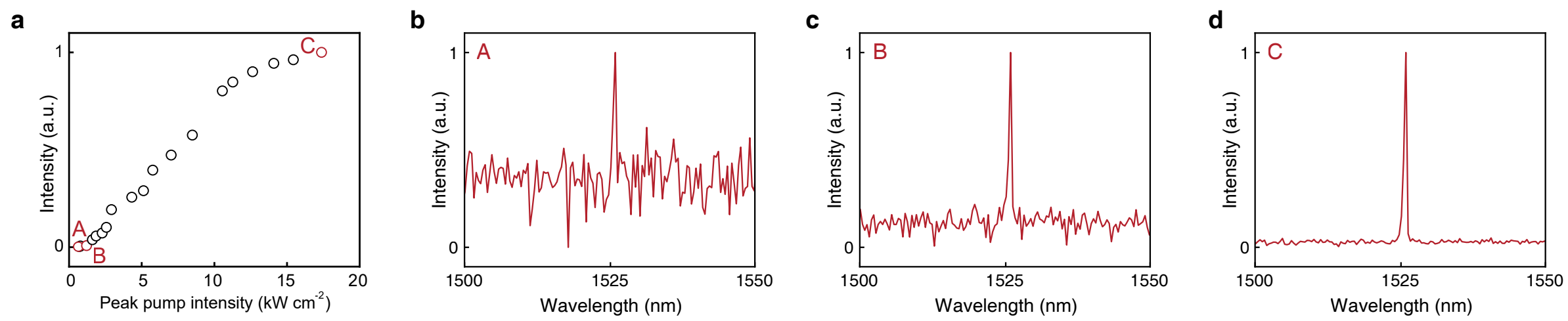

Supplementary Figure 13: **Lasing characteristics of the $|11\rangle$ mode.** (a) Measured power curve for sample X in Fig. 4 in the main text with $g = 565$ nm. The lasing mode is identified as $|11\rangle$. Three points A, B, C are marked with pumping power of 0.6, 1.2, 17.4 kW/cm^2, respectively, showing the status from spontaneous emission, the threshold lasing, to single-mode lasing (wavelength of 1526 nm). (b)(c)(d) Measured lasing spectra corresponding to A, B, C, respectively.

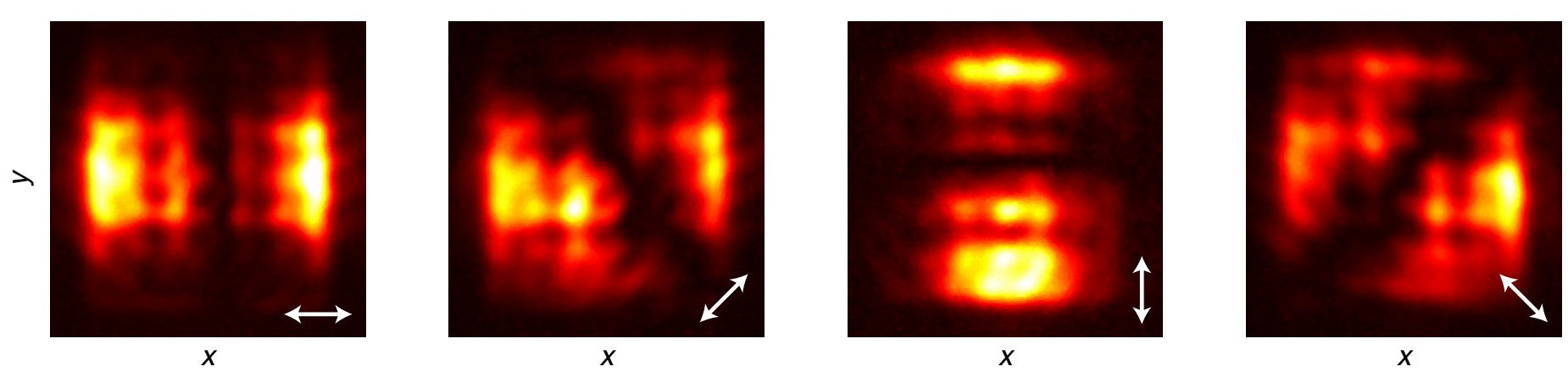

Supplementary Figure 14: **Polarization-resolved distribution of the $|11\rangle$ mode.** The polarization-resolved distribution of the $|11\rangle$ mode along 0°, 45°, 90° and 135°, respectively. All polarization components are observed to be non-zero.

Supplementary Note 12. Scalability of the flat-top mode

In the main text, the bulk region size ($N_a = 51$, while N_a represents the number of lattice periods in the bulk region) was chosen to lower the lasing threshold and enable clear pattern visualization. The flat-top mode was unambiguously confirmed both numerically and experimentally. Here, we show that $N_a = 51$ is not unique: the nontrivial fundamental mode can be robustly realized across structures of varying sizes.

We numerically evaluated the scalability of the flat-top mode using COMSOL Multiphysics. With all other parameters fixed, including the gap $g = 499$ nm, we reduced N_a from 51 to 41, 31, 21, and 11. The eigenfrequencies of the fundamental modes (Supplementary Fig. 15, red curve) remain aligning with the band edge (dashed gray line) at the Γ point with zero in-plane wavevector, yielding zero phase difference across unit cells. Modal profiles confirm the uniform field distributions, and the reflection phase θ (blue curve) stays close to π for all sizes. These results show that flat-top modes persist robustly across different lateral dimensions.

We fabricated samples with $N_a = 21$ and 51 and characterized their emission under lasing conditions. To avoid complete darkness in the bulk region —caused by Γ-BICs — the air holes were perturbed as in Fig. 5 of the main text. The near-field patterns (Supplementary Figs. 16a,c) show relatively uniform emission within the bulk, despite strong out-of-plane scattering at the boundaries from momentum mismatch. The far-field patterns (Supplementary Figs. 16b,d) display side lobes adjacent to the central lobe, consistent with the expected flat-top mode behavior.

The scalability of flat-top modes carries both fundamental and technological significance. From a research perspective, it enables flat-top modes at accessible scales, providing a versatile platform to study and validate complex quantum-mechanical concepts in classical systems.

Technologically, scalability ensures that flat-top modes can be maintained across devices of different sizes — whether enlarging cavities for higher output power or shrinking them for dense integration — simply by following identical boundary phase design rules. This greatly streamlines device design and supports large-scale, standardized fabrication. In turn, scalability enables the transfer of optimal flat-top designs across photonic devices of varying dimensions, paving the way for batch production of high-performance, uniform optoelectronic devices. Ultimately, this capability opens new opportunities in laser technology, quantum simulation, and integrated photonics.

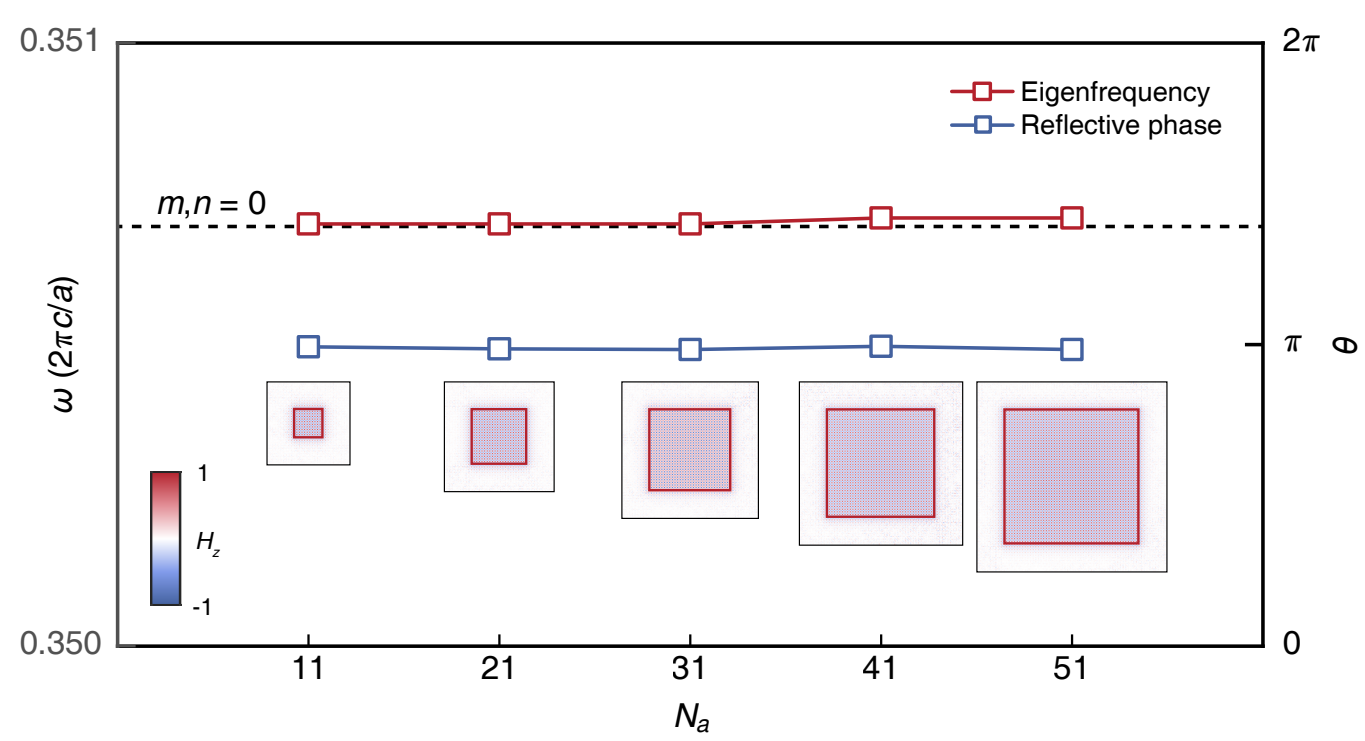

Supplementary Figure 15: **Flat-top modes at different sizes.** 2D simulation results obtained by varying the number of periodic units in the bulk region (N_a) while keeping other geometric parameters unchanged. The red curve represents the eigenfrequencies of the finite-sized modes, and the blue curve denotes the reflection phase θ calculated by the partial wave decomposition method. The field distributions are shown below. The results indicate that the finite-sized modes under different sizes are all in a flat-top mode. All results are calculated by using numerical simulation (COMSOL Multiphysics).

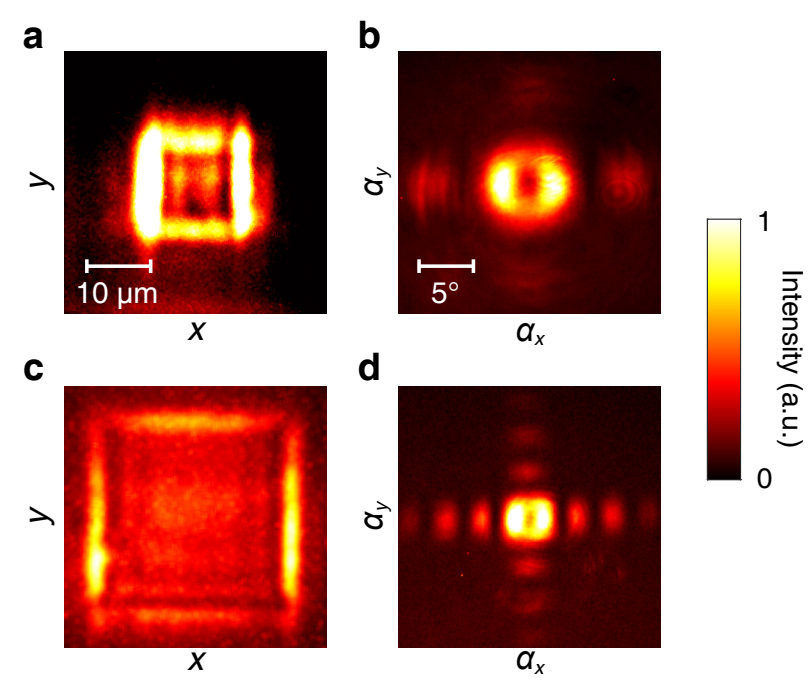

Supplementary Figure 16: **Observation of flat-top envelops at different sizes.** Observation of near-field and far-field patterns for (a)(b) $N_a = 21$ and (c)(d) $N_a = 51$ in the experiment while keeping other geometric parameters unchanged. Flat-top modes are observed in both cases.

Supplementary Note 13. Impact of group velocity on the modal profile

In the main text, we focused on boundary conditions, particularly the reflection phase θ, in shaping finite-sized modes. In principle, the morphing of wavefunctions and the realization of flat-top $|00\rangle$ have no dependence upon the bulk properties. However, in practice, it is impossible to realize perfectly reflective boundaries. In this case, the photonic band structure — especially the group velocity v_g at the operating point — also plays a critical role. Specifically, as stated in the main text, the reflective boundaries are realized through a finite-width photonic band gap (PBG). When a partial wave propagates near the boundary, it wouldn't be reflected back immediately, but partly penetrates into the boundary region with a skin depth associated with v_g: the larger v_g is, the longer the skin depth. In this way, the reflectivities of the boundaries are determined from both the v_g and the PBG width, and thus both boundary conditions and band geometry jointly impact the mode profile. In this section, we discuss the impact of v_g upon the formation of flat-top modes.

We consider a 2D PhC structure (Supplementary Fig. 17a), where circular holes (red) are arranged in a square lattice with a = 450 nm, radius r = 175 nm, and tunable refractive index n_0. The outermost column contains N = 21 holes. The slab (light gray) has a fixed permittivity n_{slab} = 3.25. Instead of using a PBG, here square-shaped air grooves (dark gray, n_{air} = 1) surrounding the central bulk act as the reflective boundaries. Specifically, the central bulk region is oriented at 45° with respect to the air grooves such that the high index contrast enables total internal reflection (TIR) of four main partial waves (yellow and blue arrows) around the second Γ point, providing in-plane confinement of finite-sized modes and introducing an additional reflection phase θ. Unlike PBG confinement which is sensitive to bandgap variations, TIR-based confinement offers stable (but still not perfect) control for modes around second Γ point.

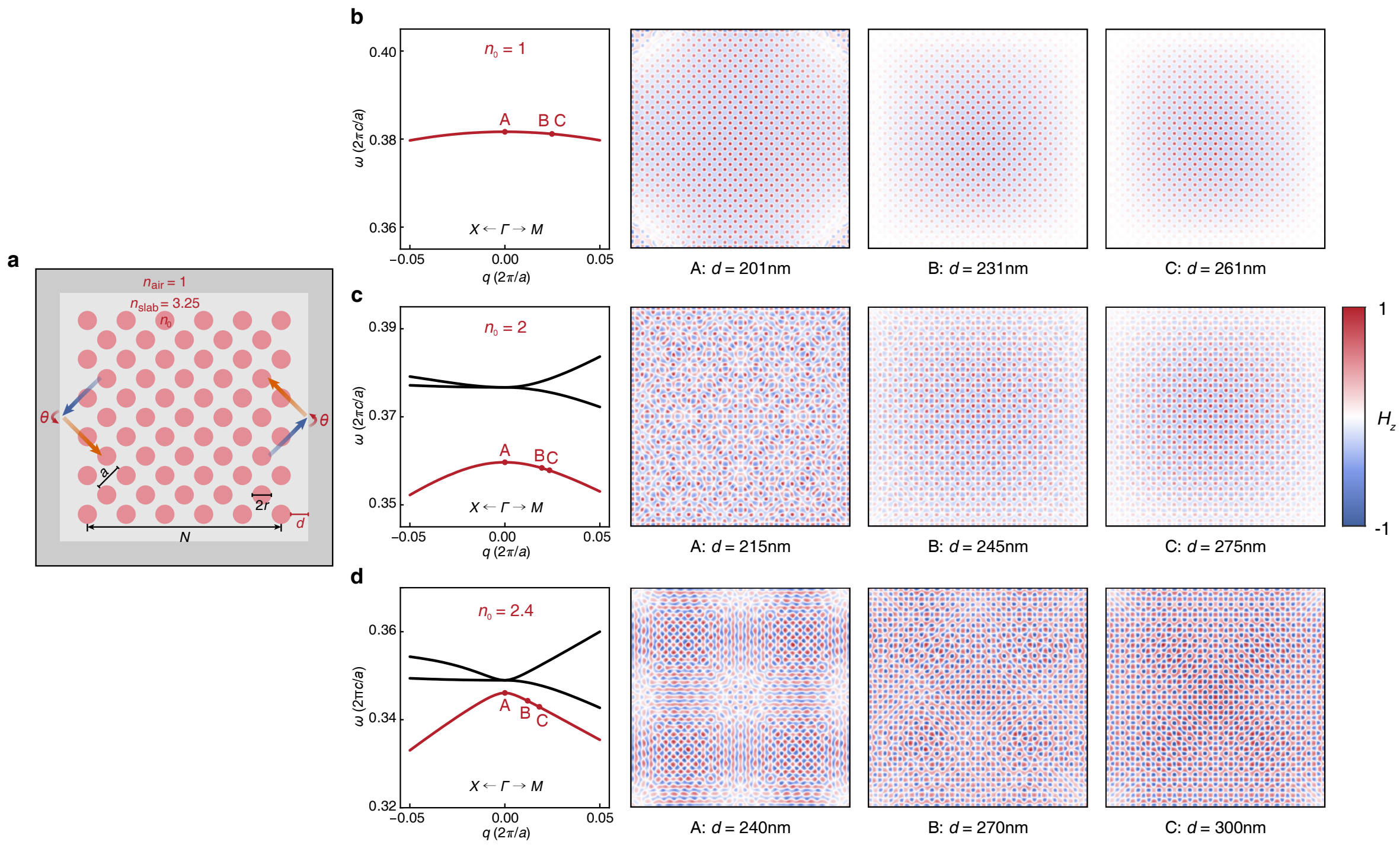

Supplementary Figure 17: **Modes with different group velocities.** (a) Configuration of a 2D PhC slab with optical confinement via TIR. Red regions indicate circular holes with tunable refractive index n_0; light-gray is the slab and dark-gray represents air grooves. Arrows show the propagation of basic waves toward the boundaries, their total internal reflection, and the resulting additional phase shift θ. (b–d) Photonic band structures and field distributions of finite-sized modes for different n_0 values, with bands supporting finite-sized modes highlighted in red. The modal profile and eigenfrequency are tuned by varying the distance d between holes and air grooves. In each case, A corresponds to the mode at the band edge, while states B and C correspond to d increased by 30 nm and 60 nm, respectively.

Here, θ can be tuned by varying the distance d between the outermost holes and the air grooves. Using this mechanism, we aim to realize the flat-top mode.

Supplementary Fig. 17b shows the band structure and field distributions of the first-order finite-sized mode. The band of interest (red solid line) in this range is relatively flat, giving a small group velocity v_g that enhances in-plane confinement. By tuning the boundary parameter d, both the reflection phase θ and in-plane wave vector q can be adjusted, thereby shaping the modal profile. When $d = 201$ nm, $q = 0$ and the mode frequency aligns with the band edge at the Γ point, where fields at different lattice sites share zero phase difference. At this point, the mode is identified as near-$|00\rangle$ mode which remains well confined but yields a broad, stable flat-top mode. The modal profile confirms such a largely uniform field distribution across the slab, with minor distortions near the corners due to boundary misalignment. Increasing d to 231 nm drives the mode into a localized state as θ and q shift, and a similar contraction is seen at $d = 261$ nm, indicating the fundamental mode $|00\rangle$ has been morphed into a well-localized $|11\rangle$ mode.

When n_0 is increased to 2, the target band (red curve) acquires stronger curvature from coupling with adjacent bands, yielding a clear quadratic dispersion (Supplementary Fig. 17c). The corresponding increase in group velocity v_g hampers the confinement. At $d = 215$ nm, the mode lies near the band edge with a broad profile, but the higher v_g causes severe boundary leakage and irregular reflection phases, producing a chaotic field distribution that precludes flat-top modes. As d increases to 245 nm and 275 nm, both θ and q shift, contracting the modal profile, reducing leakage, and stabilizing the field distribution. These results confirm that while boundary conditions can still tune finite-sized modes under quadratic dispersion, the elevated v_g makes achieving a uniform flat-top mode much more difficult.

As n_0 approaches a critical value, the three bands in Supplementary Fig. 17c converge at the Γ point to form an accidental triple degeneracy with linear conical dispersion. To avoid band inversion, we set $n_0 = 2.4$ (Supplementary Fig. 17d), where the triple degeneracy is not fully formed but the dispersion near Γ becomes nearly linear, yielding a very large group velocity v_g. At $d = 240$ nm, the finite-sized mode lies near the band edge, but the high v_g induces strong boundary leakage and disordered phase reflections, producing a highly irregular modal profile. Only at $d = 300$ nm does the mode become more organized, with a central intensity maximum that gradually decays toward the edges.

In summary, in realistic situations, the profile of finite-sized modes is jointly governed by boundary conditions and band geometry. While tuning boundary parameters effectively modifies the modal profile across all band types, the evolution differs significantly. For flat bands with low v_g, modes generally localize but can yield uniform intensity under suitable boundaries. In contrast, linear bands with high v_g could in principle replicate a unit-cell phase profile across the structure, yet strong boundary leakage produces disordered, extended fields that resist confinement. Thus, achieving uniform flat-top modes with high coherence and Q value is more robust when combining low-v_g bands with boundary tuning.
