# Supplementary material for: Wave morphing towards the flat-top envelope in photonics systems driven by artificial gauge fields
Source: Nat Commun. 2026 Jul 29;17:9178. doi: 10.1038/s41467-026-76042-0 (PMC13522590; doi:10.1038/s41467-026-76042-0)
Supplement: Supplementary file 3 [file 41467_2026_76042_MOESM3_ESM.pdf]

nature portfolio

Peer Review File

Wave morphing towards the flat-top envelope in photonics systems driven by artificial gauge fields

Corresponding Author: Professor Chao Peng

This file contains all reviewer reports in order by version, followed by all author rebuttals in order by version.

Version 0:

Reviewer comments:

Reviewer #1

(Remarks to the Author)
This manuscript addresses the general problem of wave confinement in finite systems, where boundary-induced reflections lead to discrete energy levels and spatial optical modes, called in the text wavefunctions. In conventional bounded systems, the ground state is a function of the space and its modulus square corresponds to probability distribution in a confined region. The authors aim is to engineer a nonzero-field ground state with a flat top, corresponding to a confined spatial wavefunction with transverse wavevector k\rightarrow0.

The central idea of the work is claimed to be inspired by the Byers–Yang theorem, which states that the energy levels and wave profiles of a system defined on a ring geometry vary periodically with an applied magnetic flux. I feel that this claim is an exaggeration. Phase shifts in photonic are an essential ingredient very commonly used and there is no need to invoke gauge theory, nor the magnetic field. The reality of their photonic implementation, as there is no magnetic flux, is to introduce an effective phase shift θ imposed at the boundaries of a photonic crystal. This phase shift modifies the boundary condition according to θ+2kL=2n\pi and therefore it allows the control over the transverse wavevector k. In the limit θ\rightarrow2\pi, for the n=1 mode thus k\rightarrow0, resulting in the formation of a flat-top spatial wavefunction.

To prove experimentally the existence of such a mode, the authors have fabricated a photonic crystal formed by a periodic arrangement of approximately 300 nm air holes etched into a silicon layer bonded to an InP wafer, providing optical gain around λ = 1.5 µm. The bulk region of the crystal is distinguished from the boundary region by a different lattice period, and the distance g between two holes at the bulk–boundary transition acts as the control parameter for the effective phase shift. Numerical simulations demonstrate that tuning g allows a fine tuning of the transverse energy levels. A single longitudinal mode (perpendicular to the crystal plane) that falls within the photoluminescence band is selected. By fabricating samples with different values of g, the authors investigate the near-field and far-field emission profiles under optical pumping, aiming to experimentally demonstrating the formation of a flat-top spatial mode.

From a conceptual standpoint, the theoretical idea of introducing a boundary-induced phase shift in a photonic crystal is interesting, however it has been already exploited in many different situations in p. The analogy with a simple ring model is helpful, and the numerical simulations are convincing on the possibility of designing a flat top mode. Yet, I have some serious doubt about the interest of such a mode, which, as I will explain later, does not produce any significant improvements in mode quality. Finally, I have significant concerns regarding the experimental validation of the proposed concept.

First, in the simulations the parameter g is varied from 675 nm down to 423 nm, whereas in the experiments it spans only from 565 nm to 430 nm. The reason for this reduced experimental range is not explained. As a result, the experimental data do not show a clear evolution of the spatial mode profile as a function of g, which is somewhat disappointing given the central role of this parameter in the theoretical framework.

Second, the transverse confinement length of the structure is approximately L = 30 µm. This suggests that several transverse modes should be supported, since the corresponding transverse wavevectors k is much larger than 2\pi/L. It is therefore unclear how the authors are able to isolate and track a single transverse spatial mode experimentally. This point should be clarified.

Third, the experimentally measured spatial mode profiles do not appear to closely match the simulated ones. More importantly, the main objective of the paper is to demonstrate the formation of a flat-top spatial mode, yet the mode shown in Figure 4c (Z) exhibits essentially zero field intensity in the central region. Instead, the experimental data consistently show that the optical intensity is concentrated at the edges of the structure, i.e., near the boundaries. It is not obvious that such a mode is of practical interest from a device perspective. Indeed, the far-field emission is dominated by the edge emission and displays a diffraction pattern that is typically undesirable in photonic devices.

The authors attribute the absence of intensity in the central region to the formation of a bound state in the continuum (BIC) that suppresses radiation losses. However, the physical mechanism leading to the formation of this BIC is not clearly explained. Moreover, to substantiate the claim of a BIC, additional experimental evidence should be provided. For instance, measurements of the quality factor or a systematic study of the laser threshold, as the system approaches the supposed BIC condition, would significantly strengthen the manuscript.

In conclusion, while the manuscript presents an interesting and original theoretical idea supported by convincing numerical simulations, the experimental results are not enough to validate the proposed concept. The central claim of realising a BIC flat-top spatial mode is not convincingly demonstrated, and the experimental observations raise doubts about the physical interpretation, particularly regarding mode selection and the claim of a bound state in the continuum. Key aspects of the experimental design, parameter space, and supporting evidence remain insufficiently explained. Given the gap between the theoretical proposal and the experimental validation, I do not believe that the present work meets the level of completeness and impact required for publication in Nature Communications. I therefore do not recommend the manuscript for publication.

Reviewer #2

(Remarks to the Author)
This manuscript reports an interesting and compelling experimental and theoretical study on wave morphing toward a flat-top ground state in laterally confined photonic crystal slabs, enabled by an artificial gauge field implemented via boundary reflection phases. By invoking the Byers–Yang theorem and mapping the system to an effective ring model, the authors demonstrate continuous energy-level shifting and experimentally probe the eigenstate evolution through lasing emission. The work is technically strong, clearly presented, and experimentally impressive. Thus, I recommend acceptance of this manuscript after the authors clarify some conceptual and interpretational points to further strengthen the manuscript.

1. A central result is the observation of lasing modes approaching a near-flat-top ground state (notably sample Z). While this is an intriguing manifestation of gauge-field-induced wave morphing, its practical relevance for lasing applications remains somewhat ambiguous. In particular, a flat-top ground state corresponds to vanishing in-plane wavevector and isotropically suppressed group velocity, closely resembling a band-edge or frozen mode. Such states typically suffer from weak mode selectivity and may be unfavorable for robust, high-quality single-mode lasing once issues such as gain competition and saturation are considered. Thus, the authors may wish to clarify: whether the flat-top ground state is envisioned primarily as a linear-wave or passive eigenstate, rather than a practical lasing mode; or whether specific gain, loss, or symmetry-breaking mechanisms (beyond those briefly discussed in Fig. 5) could stabilize such a mode for lasing.
A short discussion acknowledging these limitations would improve the physical interpretation and avoid overextending the technological claims.

2. The manuscript briefly connects the observed flat-top states to non-Hermitian physics and mentions skin-effect-induced localization. However, I assume the flat-top ground states demonstrated here are qualitatively different from conventional non-Hermitian skin modes. Although the authors correctly note that non-Hermiticity becomes relevant when boundary leakage is present, the relationship between these two phenomena could be articulated more clearly. It would be helpful if the authors can clarify this explicitly: the flat-top distributions are not skin modes in the usual sense, and they arise from gauge-field-induced spectral shifting and effective Neumann boundary conditions rather than non-reciprocal pumping? Such clarification would help place the work more precisely within the broader non-Hermitian photonics literature.

3. The discussion of bound states in the continuum (BICs) is appropriate and well supported, particularly in connection with band-edge lasing and radiation suppression near the $|00\rangle$ state. However, recent studies have shown that higher-order topological phases can also host BICs (for instance, Cerjan et al., Phys. Rev. Lett. 125, 213901, 2020; Hu, et al. Light Sci Appl , 10, 164, 2021), including corner- or hinge-localized BICs protected by crystalline symmetries. Given that the present system already relies on symmetry, boundary engineering, and band topology, the authors may consider briefly commenting on: whether higher-order topological BICs could be realized in similar heterogeneous photonic crystal geometries; or whether such modes could coexist or compete with the flat-top ground state demonstrated here.
Even a short qualitative remark and one or two representative citations would broaden the conceptual scope of the work.

4. Some fonts in figures seem to be too small without zoom-in for clear visualization.

Overall, this is a strong and elegant contribution, combining clear theoretical insight with convincing experimental validation. Addressing the points above would mainly improve conceptual clarity and positioning, rather than requiring new experiments. I recommend publication after minor revision.

Reviewer #3

(Remarks to the Author)
The manuscript presents a general mechanism for tuning energy levels and wave morphology via geometric phase, and demonstrates, both theoretically and experimentally, the controlled morphing of optical waves into a flat-top ground state via an artificial gauge field. By engineer- ing boundary reflections, a spatially uniform, non-zero ground state can be realized in a finite, confined system. That is counterintuitive and opens new avenues for light manipulation. I find this present work integrates the core concept of gauge field theory from condensed matter physics with the authors' prior researches (including BIC modes, heterogeneous integration techniques, and optical field manipulation), thereby establishing a novel research paradigm.
All in all, this work is conceptually novel, technically sound, and represents a significant contribution to the fields of photonics, wave physics, and quantum simulation. The manuscript is, also, well and clearly written, even for non-specialist readers, and minor suggestions and comments include:

1. Figure 2c and the experimental section only present the numerical values of g and the corresponding phenomena. The values of θ (e.g., θ = π) appear to be derived from simulation or theoretical inference. Readers cannot ascertain, for instance, how θ increases linearly or nonlinearly from 0 to π as g changes from 565 nm to 474 nm. Other researchers may be confused about accurately predicting the gauge field state of their system when given a specific value of g.

2. The identification of the "near-flat-top state" in Fig. 4 relies heavily on the minimal divergence angle (1.2°), but no quantitative metric for absolute field uniformity(e.g., intensity variance) is provided. The statement that "the bulk region appears almost dark" raises the question: does this imply energy is primarily confined at the boundary? If so, how does this align precisely with the definition of a "flat-top" state distributed throughout the bulk? Furthermore, in the mapping from the ring model to the two-dimensional photonic crystal, the functional relationship between the reflective phase (θ) and the gap spacing (g) is not explicitly provided in the main text and is only illustrated through simulation.

Version 1:

Reviewer comments:

Reviewer #2

(Remarks to the Author)
I have read the authors' response and the revised manuscript carefully, and I am satisfied that the authors have adequately addressed the concerns raised in my previous review. In particular, the revised manuscript now provides a much clearer and more balanced discussion regarding the physical interpretation and practical implications of the flat-top ground states, especially in the context of lasing applications and gain–loss engineering. The distinction between the gauge-induced flat-top modes demonstrated here and conventional non-Hermitian skin modes has also been clarified substantially, which improves the conceptual positioning of the work within the broader non-Hermitian photonics literature.
I also appreciate very much the authors' expanded discussion on the relation between the present platform and bound states in the continuum (BICs), including the possible extension toward topologically nontrivial systems.

Overall, the revisions have significantly improved the clarity and presentation of the manuscript without altering its core contributions. In my opinion, the manuscript now represents a strong and elegant contribution combining clear theoretical insight with convincing experimental validation. I therefore support publication of this revised version in Nature Communications.

Reviewer #3

(Remarks to the Author)
The authors have fully considered and have provided well-organized responses to all comments. They adequately address concerns about the "θ–g relationship", the "quantitative validation of flat-top profiles", and the "interpretation of near-field patterns". And the additional discussion is clear and logic also for non-experts on the topic. The response demonstrates strong physical understanding, appropriately supplements theoretical modeling and experimental data, and aligns fully with the standards expected in a high-quality revision.

Open Access This Peer Review File is licensed under a Creative Commons Attribution 4.0 International License, which permits use, sharing, adaptation, distribution and reproduction in any medium or format, as long as you give appropriate credit to the original author(s) and the source, provide a link to the Creative Commons license, and indicate if changes were made.
In cases where reviewers are anonymous, credit should be given to 'Anonymous Referee' and the source.
The images or other third party material in this Peer Review File are included in the article's Creative Commons license, unless indicated otherwise in a credit line to the material. If material is not included in the article's Creative Commons license and your intended use is not permitted by statutory regulation or exceeds the permitted use, you will need to obtain permission directly from the copyright holder.
To view a copy of this license, visit https://creativecommons.org/licenses/by/4.0/

Reply to Referee 1

Specific comments and questions

COMMENT 1.A

> This manuscript addresses the general problem of wave confinement in finite systems, where boundary-induced reflections lead to discrete energy levels and spatial optical modes, called in the text wavefunctions. In conventional bounded systems, the ground state is a function of the space and its modulus square corresponds to probability distribution in a confined region. The authors aim is to engineer a nonzero-field ground state with a flat top, corresponding to a confined spatial wavefunction with transverse wavevector $k \to 0$.
>
> The central idea of the work is claimed to be inspired by the Byers-Yang theorem, which states that the energy levels and wave profiles of a system defined on a ring geometry vary periodically with an applied magnetic flux. I feel that this claim is an exaggeration. Phase shifts in photonic are an essential ingredient very commonly used and there is no need to invoke gauge theory, nor the magnetic field. The reality of their photonic implementation, as there is no magnetic flux, is to introduce an effective phase shift θ imposed at the boundaries of a photonic crystal. This phase shift modifies the boundary condition according to $\theta + 2kL = 2n\pi$ and therefore it allows the control over the transverse wavevector k. In the limit $\theta \to 2\pi$, for the $n = 1$ mode thus $k \to 0$, resulting in the formation of a flat-top spatial wavefunction.

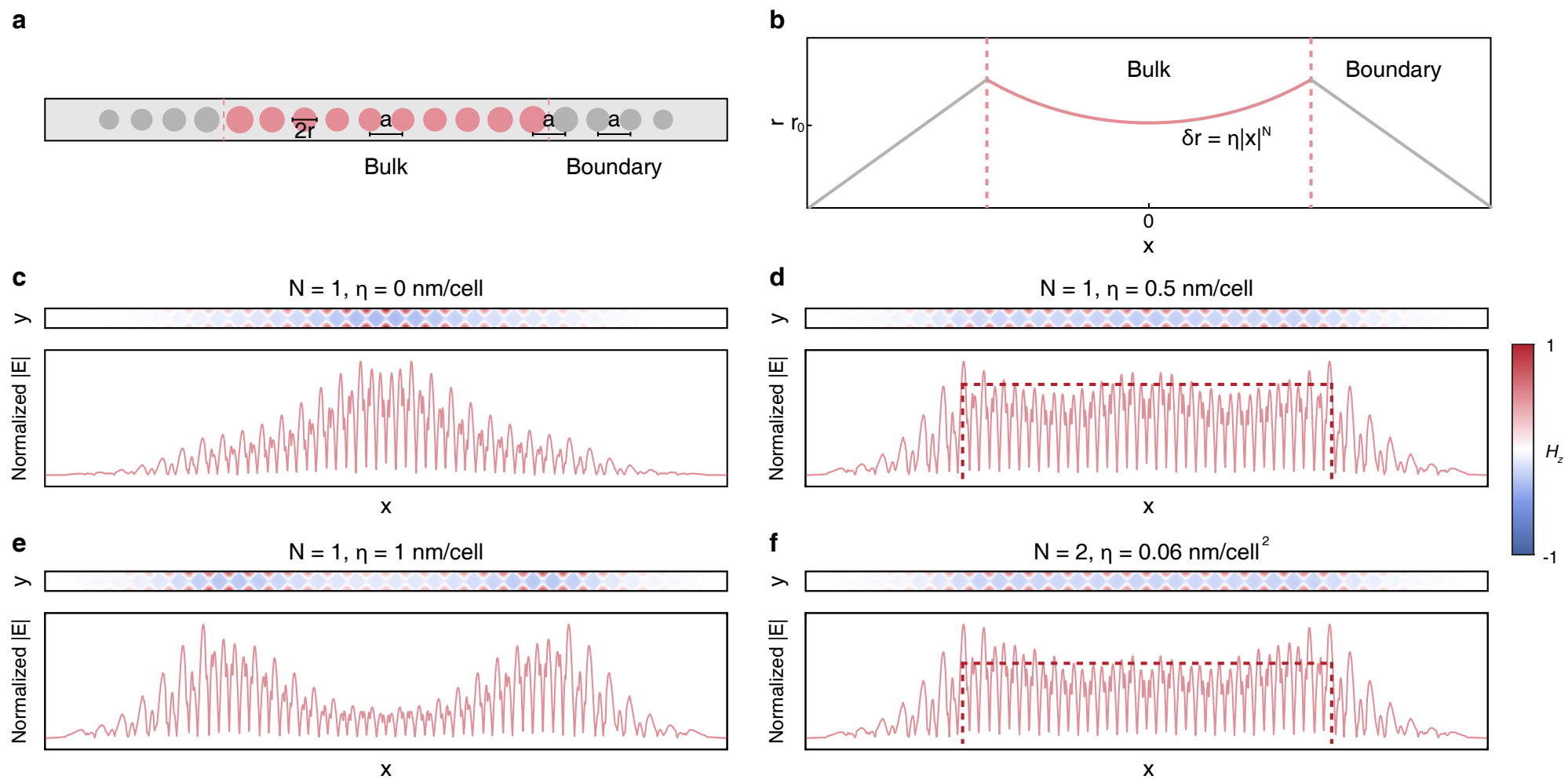

Figure R1 **Figure S2 in the Supplementary Material.** Generation of flat-top modes via distributed spatial modulation of permittivity in a 1D hetero PhC cavity. (a) Schematic of the cavity, with uniform crystal constant and modulated air-hole radius. (b) Spatial modulation function of hole radius r, showing a symmetric increase as $\delta r = \eta|x|^N$ followed by a sharp drop at the boundary region. (c–e) Mode envelopes for modulation order $N = 1$ with different modulation coefficients η: Gaussian-like profile ($\eta = 0$), flat-top-like profile ($\eta \approx 0.5$ nm/cell), and boundary-enhanced profile ($\eta = 1$ nm/cell). (f) Flat-top-like mode envelope achieved for $N = 2$ with $\eta = 0.06$ nm/cell^2.

REPLY 1.A

> Thanks for your insightful comments. We fully agree that, in our specific implementation presented in the main text, the formation of flat-top modes can indeed be understood from a boundary-condition perspective, namely, an effective reflection phase shift at the boundaries modifies the resonance and consequently leads to a flat-top envelope. This interpretation is correct and intuitive.

However, we would like to clarify that we invoke the gauge perspective not to overstate the magnetic analogies, but rather to provide a general description for the flat-top envelope formation, since we found that such a phenomenon is not restricted to boundary phase shifting, but can also arise from more general distributed permittivity modulations within the cavity where the boundaries are not well-defined.

To illustrate this point, we provide another example, which is a 1D hetero photonic crystal (PhC) cavity (Fig. R1a) where the lattice constant is kept unchanged across the whole structure, and a smooth spatial modulation of the hole radius r is introduced (Fig. R1b): in the cavity region, the hole radius symmetrically increases as $\delta r = \eta|x|^N$, followed by a sharp drop to form a photonic band gap. Here η denotes the modulation coefficient; integer N denotes the modulation order; x is the spatial coordinate of each hole. This modulation induces an effective permittivity variation $\delta n(x)$, which continuously modifies the cavity mode profile. As shown in Fig. R1c-e, different η lead to different envelope shapes: when $N = 1$, a Gaussian-like profile for $\eta = 0$, a flat-top-like profile at $\eta \approx 0.5$ nm/cell, and a boundary-enhanced profile at larger η (1 nm/cell). For $N = 2$, we can also create a flat-top-like profile by turning $\eta = 0.06$ nm/cell^2 (Fig. R1f). This demonstrates that flat-top modes can emerge without explicitly invoking boundary phase shifts, but rather from distributed permittivity modulation.

To elaborate on the physical origin of flat-top modes, we consider a transverse mode near the Γ point, expressed as a superposition of counter-propagating Bloch components:

$$E = R(x)\mathrm{e}^{ikx} + S(x)\mathrm{e}^{-ikx} \tag{R1}$$

where $R = |R|\mathrm{e}^{i\theta_R}$, $S = |S|\mathrm{e}^{i\theta_S}$, and $\phi = \theta_R - \theta_S$ is the relative phase. As stated above, we introduce a weak spatial permittivity modulation $\delta n(x)$ by gradually altering the hole radius, giving rise to a position-dependent wavevector detuning $\delta k(x) \propto \delta n(x)$. Thus, the coupled-mode equation can be derived as:

$$\frac{dR}{dx} = i\kappa S + i\delta k(x)R \qquad \frac{dS}{dx} = -i\kappa^\dagger R - i\delta k(x)S \tag{R2}$$

where κ denotes the Bragg coupling, coming from the distributed feedback caused by the lattice structure itself. Since the modulation is relatively weak, we assume κ is constant, and Eq.R2 can be further rearranged as:

$$i\frac{d}{dx}\begin{pmatrix} R \\ S \end{pmatrix} = -[\mathrm{Re}[\kappa]\sigma_x - \mathrm{Im}[\kappa]\sigma_y + \delta k(x)\sigma_z]\begin{pmatrix} R \\ S \end{pmatrix} \tag{R3}$$

Clearly, the detuning $\delta k(x)$ serves as an effective "mass" term. By employing a gauge transformation:

$$R(x) \to \tilde{R}(x) = R(x)\mathrm{e}^{i\theta(x)} \qquad S(x) \to \tilde{S}(x) = S(x)\mathrm{e}^{-i\theta(x)} \tag{R4}$$

and letting $\theta'(x) = \delta k(x)$, we obtain:

$$\frac{dR}{dx} = i\tilde{\kappa}S \qquad \frac{dS}{dx} = -i\tilde{\kappa}^\dagger R \tag{R5}$$

where the detuning term is not eliminated, but rather transferred into a position-dependent phase of the coupling coefficient:

$$\tilde{\kappa} = \kappa\mathrm{e}^{-2i\theta(x)} = \kappa\mathrm{e}^{-2i\int \delta k(x)dx} \tag{R6}$$

This transformation shows that the real-space modulation can be equivalently interpreted as a spatially varying phase in the coupling term, analogous to a Peierls phase in

tight-binding systems [R1–R3]. In this sense, $\delta k(x)$ plays the role of an effective gauge potential, although no real magnetic field is involved.

According to Eq. R4, the gauge transformation actually turns the spatial permittivity modulation into an additional phase difference between the two counter-propagating Bloch components:

$$\phi = 2kx + 2\int \delta k(x)dx \tag{R7}$$

where the first term represents the dynamic phase, which oscillates rapidly and does not contribute to the slowly varying envelope. From this perspective, both the boundary phase shifts and distributed permittivity modulations act as different ways of engineering the same quantity: the relative phase between coupled Bloch components, which governs their interference and thus determines the envelope profile.

In the literature, similar flat-top or plateau-like wave distributions have been reported in a variety of wave systems where phase accumulation or effective gauge potentials play a central role. For example, in systems exhibiting Bloch oscillations or Wannier-Stark localization [R4–R6], a linear potential induces a position-dependent phase accumulation, leading to strongly reshaped spatial envelope profiles. Such behavior has been demonstrated in both discrete lattice structures [R7, R8] and chirped photonic systems [R9]. As illustrated in Fig. R2, a flat-top-like envelope can be realized in an array of circularly curved optical waveguides, where the curvature induces an effective gauge potential that generates a spatial phase gradient and consequently reshapes the modal envelope [R8]. These observations consistently agree with our picture that the root origin of flat-top-like profiles is an appropriate phase accumulation, or the effective gauge potential.

In addition, it is well established that position-dependent Peierls phases can modify the spatial modal distributions in photonic lattices with synthetic gauge fields [R10–R12]. As pointed out in Ref. [R12], "the presence of a gauge field can fundamentally modify wave localization even in topologically trivial systems", highlighting that gauge-induced phase indeed can reshape modal profiles. In this context, our approach can be understood as a continuous analogue of Peierls-phase engineering.

As shown in Fig. R3, the gap introduced in our experimental design can be regarded as an attractive potential at the bulk-boundary interface, giving rise to a δ-like modulation of $\delta k(x)$. While this implementation is not strictly adiabatic around the boundaries, it effectively produces the required phase shift in a compact form, which is compatible with fabrication constraints. In fact, realizing a small modulation rate (i.e. $\eta \approx 0.5$ nm/cell for $N = 1$) is beyond our fabrication capability. Although the abrupt changes around the bulk-boundary interface create scatterings, they do not qualitatively affect the formation of the flat-top mode in the central region. We will explain how to relieve these interface scatterings in the following responses.

In summary, we believe that the gauge picture provides a general perspective for understanding the flat-top envelope formation, and suggests that such a principle holds from photonic system to other wave systems. The flat-top envelope formation does not rely on a specific implementation, but rather arises from a common mechanism: spatial engineering of phase gradients or effective momentum distributions. This unified phase-engineering perspective may also provide a general guideline for tailoring wave envelopes in a wide class of wave systems. At the same time, we fully understand the reviewer's concern regarding the use of gauge-related concepts. To avoid any possible misunderstanding, we have updated the manuscript to improve the clarity, and have added a new section in Supplementary Material Section 3, "Continuous gauge transformation and effective potential", to clarify our central idea of envelope morphing via phase engineering.

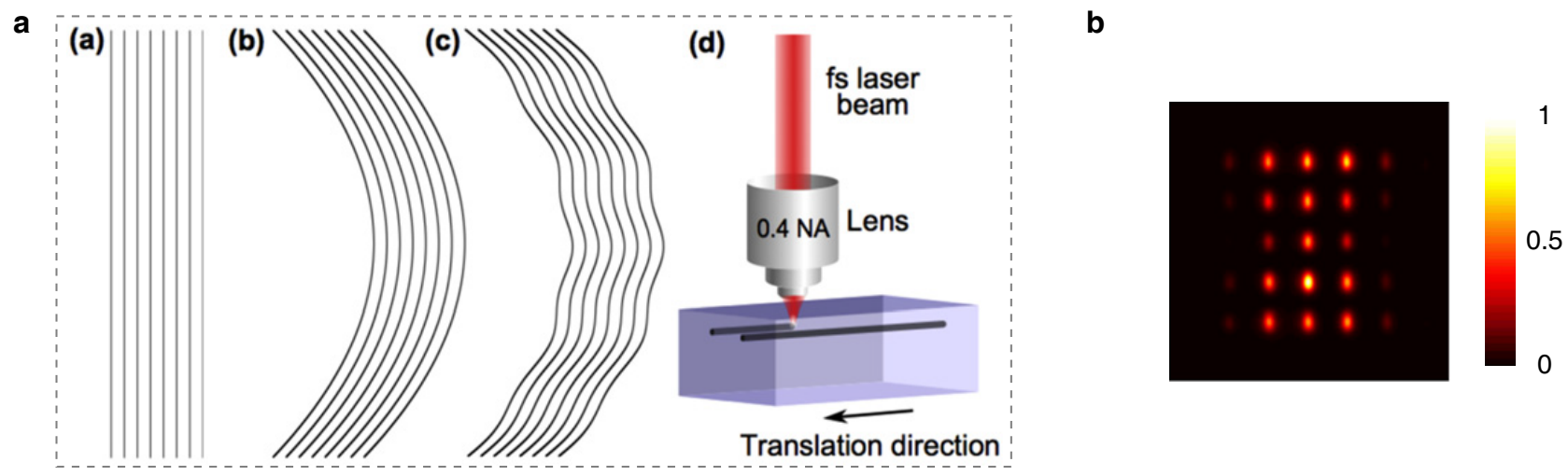

Figure R2 An example of the flat-top-like profile in engineered phase accumulation [R8]. (a) Schematic diagrams of arrays of circularly curved optical waveguides. (b) Flat-top-like output intensity distribution.

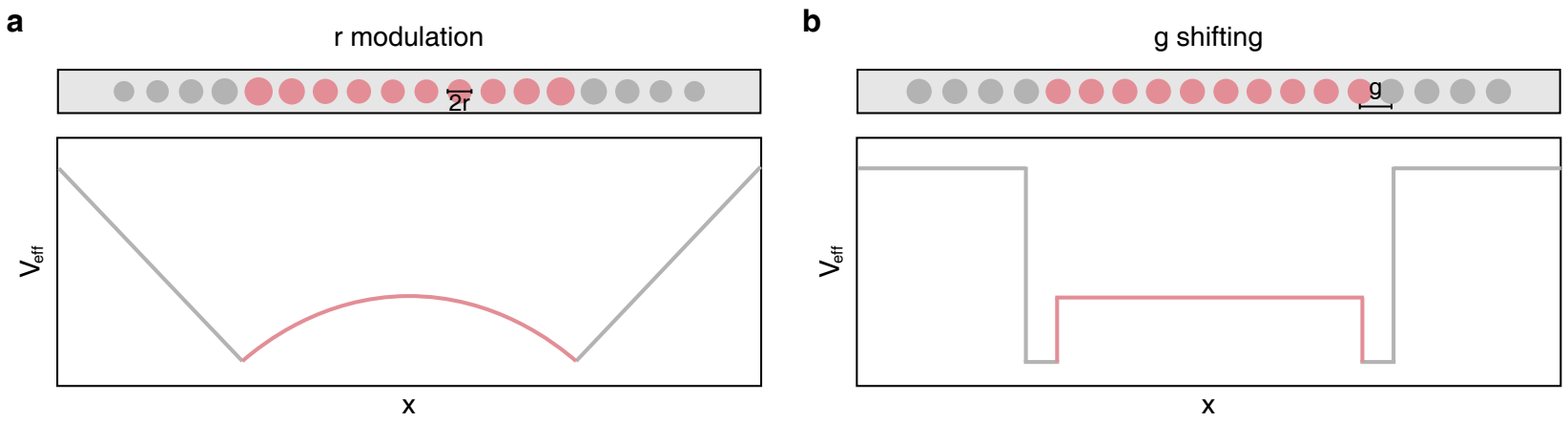

Figure R3 **Figure S3 in the Supplementary Material.** Effective gauge potential introduced by spatial engineering with (a) r modulation and (b) g shifting (our experimental design).

COMMENT 1.B

> To prove experimentally the existence of such a mode, the authors have fabricated a photonic crystal formed by a periodic arrangement of approximately 300 nm air holes etched into a silicon layer bonded to an InP wafer, providing optical gain around $\lambda = 1.5$ μm. The bulk region of the crystal is distinguished from the boundary region by a different lattice period, and the distance g between two holes at the bulk–boundary transition acts as the control parameter for the effective phase shift. Numerical simulations demonstrate that tuning g allows a fine tuning of the transverse energy levels. A single longitudinal mode (perpendicular to the crystal plane) that falls within the photoluminescence band is selected. By fabricating samples with different values of g, the authors investigate the near-field and far-field emission profiles under optical pumping, aiming to experimentally demonstrating the formation of a flat-top spatial mode.
>
> From a conceptual standpoint, the theoretical idea of introducing a boundary-induced phase shift in a photonic crystal is interesting, however it has been already exploited in many different situations in p. The analogy with a simple ring model is helpful, and the numerical simulations are convincing on the possibility of designing a flat top mode. Yet, I have some serious doubt about the interest of such a mode, which, as I will explain later, does not produce any significant improvements in mode quality. Finally, I have significant concerns regarding the experimental validation of the proposed concept.

REPLY 1.B

> We thank the reviewer for his/her detailed comments. We appreciate the reviewer's summary of our work, and fully understand the concerns regarding both the conceptual significance and the experimental validation. We would clarify several key points here.
>
> First, we clarify that our cavity supports a set of transverse modes along (x, y), each associated with a discrete energy level (Fig. 1c in the main text). These modes can be understood as collective guided resonances [R13, R14], where the presence of

boundaries or spatial permittivity modulation breaks the translational symmetry of the PhC slab and leads to the hybridization of multiple Bloch components. As a result, the spatial envelopes of transverse cavity modes are governed by the relative phase between these Bloch components. From this point, transverse modes bear similarity to Wannier-like states, and the spatial phase modulation can be interpreted as an effective gauge potential acting on the underlying Bloch components.

Second, we clarify that the interface gap g in our design is not limited to the conventional boundary engineering as widely explored in the literature. Instead, as discussed in our response to comment 1.A, the gap g serves as a fabrication-friendly implementation of phase-gradient control, which ultimately governs the spatial interference between counter-propagating Bloch components and determines the envelope profile in the central cavity region. This is consistent with the general concept of phase-engineered envelope shaping.

Third, the preliminary objective of this work is not aimed at improving the mode quality metrics (such as linewidth or threshold), but rather to experimentally verify the physics of controlled envelope morphing. The flat-top mode represents a distinct class of spatial distributions, characterized by a nearly uniform envelop in the central region and rapid decay near the boundaries. Such profiles are fundamentally different from conventional Gaussian-like modes and are of interest in scenarios requiring uniform field distribution, such as nonlinear interactions, uniform gain utilization, and beam shaping.

Regarding the experimental validation, we demonstrate the flat-top mode in a laser scheme, by directly probing both the near- and far-field patterns of the lasing beam under controlled variation of g. The observed transition towards flat-top-like profiles, together with its systematic dependence on g, is consistent with our theoretical predictions and numerical simulations, providing direct evidence that the observed mode shaping originates from phase engineering. We further clarify these points in detail in the following responses.

COMMENT 1.C

First, in the simulations the parameter g is varied from 675 nm down to 423 nm, whereas in the experiments it spans only from 565 nm to 430 nm. The reason for this reduced experimental range is not explained. As a result, the experimental data do not show a clear evolution of the spatial mode profile as a function of g, which is somewhat disappointing given the central role of this parameter in the theoretical framework.

REPLY 1.C

Thanks for raising this important point. We fully agree that the range of the experimentally varied gap g does not exactly match that in the 2D simulations, and we appreciate the opportunity to clarify this discrepancy.

The primary reason for the mismatched g range lies in the intrinsic differences between the ideal 2D model and the actual 3D sample. In fact, the effective reflection phase θ depends on both the geometric gap g and the band structures of PhC slab, related to both the band gap width Δ at boundaries and the group velocity v_g of the Bloch band in the central cavity. The 2D simulations adopt a simplified model that captures the essential physics of phase engineering and the resulting energy level shifting. In contrast, the experimental hetero-bonded structure has a complicated vertical geometry, consisting of a patterned Si layer, a III-V epitaxial layer that provides optical gain, and a substrate. Obviously, the band structures of the 3D device differ from the simplified 2D model. As a result, the range of g required to sweep θ from 0 to 2π differs between the ideal 2D model and the actual 3D device.

We note that a full 3D simulation of the entire heterogeneous cavity is computationally prohibitive due to the mesh resolution limit. Therefore, in the experiments on actual samples, we map the observed envelopes to the evolution of transverse modes. We believe the selected experimental range sufficiently captures a complete evolution from the $|11\rangle$ mode to the near-$|00\rangle$ mode.

As a supplement to a wider range, we have fabricated an additional sample with $g = 580$ nm (denoted as $\mathcal{S}$) and characterized its lasing characteristics. The near-field (NFP) and far-field patterns (FFP), along with those from the original six samples shown in the main text, are summarized in Fig. R4. The divergence angles of FFPs are plotted in Fig. R5. Clearly, the divergence angle ($\sim 2.0°$) of sample $\mathcal{S}$ is nearly identical to that of sample $\mathcal{X}$ ($g = 565$ nm), and both of them are very close to the theoretical result of $|11\rangle$ ($\sim 2.2°$), indicating that for $g \geq 565$ nm, the mode has already stabilized and asymptotically approaches the $|11\rangle$ mode. Similar asymptotic behavior could also be found in 2D simulation. As shown in Fig. 2c in the main text and Fig. S6 in the Supplementary Material, when $g \geq 580$ nm, the wavelengths and modal profiles remain virtually unchanged. Therefore, either sample $\mathcal{S}$ or $\mathcal{X}$ indeed serves as a faithful experimental representation of the $|11\rangle$ mode.

For the other branch of evolution (blue solid line, Fig. R5 and Fig. 4d in the main text), sample $\mathcal{W}$ indeed does not approach the theoretical $|11\rangle$ mode as sample $\mathcal{S}$ does. This is because further reducing g below 430 nm would violate the fabrication constraints on the PhC's filling factor, preventing reliable device fabrication. Nevertheless, samples $\mathcal{U} - \mathcal{V} - \mathcal{W}$ have already catch the evolution trends from $|33\rangle$ to $|11\rangle$, by showing a decreasing divergence angle.

We hope the additional data and explanation clarify the consistency between our experimental observations and the underlying theoretical framework. To avoid any misunderstanding, we add a sentence on page 15 to clarify the distinctions between the simulation and experimental range of g.

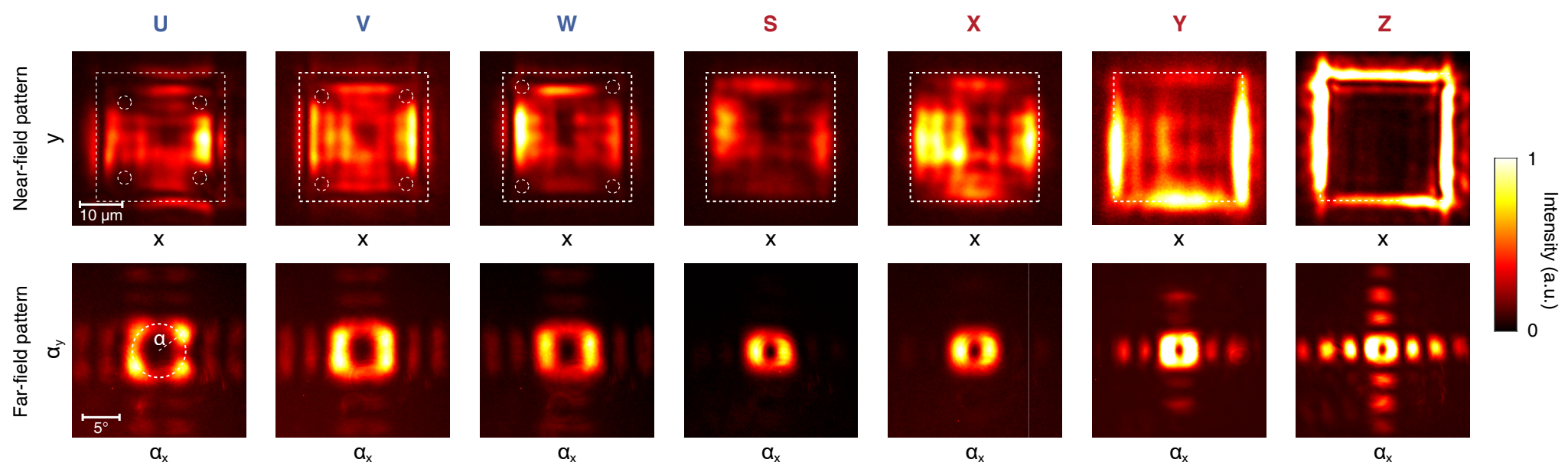

Figure R4 Near-field and far-field patterns of samples with varying gap spacing g (sample $\mathcal{S}$ with $g = 580$ nm included).

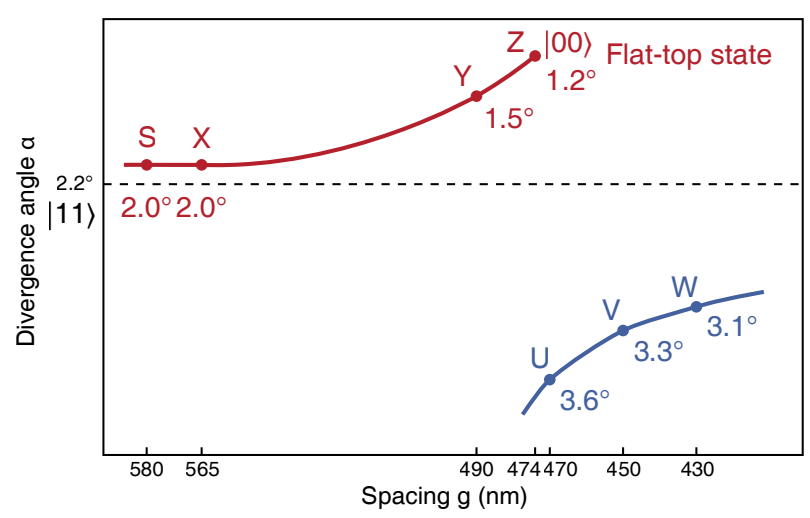

Figure R5 Divergence angle α as a function of gap spacing g (sample $\mathcal{S}$ with $g = 580$ nm included).

COMMENT 1.D

> Second, the transverse confinement length of the structure is approximately $L = 30\ \mu$m. This suggests that several transverse modes should be supported, since the corresponding transverse wavevectors k is much larger than $2\pi/L$. It is therefore unclear how the authors are able to isolate and track a single transverse spatial mode experimentally. This point should be clarified.

REPLY 1.D

Thanks for this insightful comment. We agree that a PhC cavity with a size of approximately $L = 30\ \mu$m can, in principle, support multiple transverse modes. We hereby present a detailed clarification on the mode selectivity.

As shown in Fig. 2c in the main text, the continuous momentum space becomes discrete by an interval of $k = \pi/L$ [R13], supporting a set of transverse cavity modes with different quality factors (Qs) and modal volumes (Vs). When L is relatively large, the free spectral range between two neighboring modes is quite small (~ 2 nm), so a single mode can hardly be isolated using a conventional optical filter. To address this, we apply an optically-pumped laser scheme to select the mode from mode competition; then we can trace a single transverse mode and probe its near-field and far-field patterns.

Specifically, the lasing mode selection is governed by multiple factors, primarily including the Q factor and the spatial overlap Γ_{active} with the gain region. In general, the mode with the highest Q and largest Γ_{active} tends to prevail in the lasing. In our design, the modal Q factor is determined by both radiative loss and non-radiative loss, i.e., the side leakage, random scatterings, and material absorption. For a set of cavity modes, the non-radiative loss is comparable due to the same level of fabrication imperfections and material properties. In addition, the optical pump spot is intentionally chosen to be sufficiently large to cover the entire cavity region. As a result, difference in the radiative Q becomes the dominant factor in determining the lasing mode.

We focus on the first Bloch band, where a bound state in the continuum (BIC) with infinite radiative Q residing at the Γ point, and thus the lowest-order transverse mode exhibits strongly suppressed radiation and therefore possesses the highest radiative Q. As shown in Fig. 2c in the main text, when the boundary phase shift $\theta < \pi$, the modes on the $|11\rangle \to |00\rangle$ trajectory have the highest radiative Qs, and thus dominate the lasing. This is consistent with our experiments: For samples $\mathcal{X}$ through $\mathcal{Z}$ with larger g, relatively small divergence angles are observed, indicating that the lasing mode belongs to the lowest energy level, following the $|11\rangle \to |00\rangle$ branch.

When $\theta > \pi$, the original $|11\rangle$ and $|22\rangle$ modes tend to localize at the bulk-boundary interfaces and corners, suffering severe in-plane leakage and rapid annihilation (see Supplementary section 8, "Non-Hermitian ring model" for details). Instead, the original $|33\rangle$ mode (now $|22'\rangle$ mode) gradually evolves into a newborn $|11\rangle$ mode, keeping the highest Q and thus dominate the lasing action. This is also consistent with our experiments: For samples $\mathcal{U}$ through $\mathcal{W}$ with smaller g, larger divergence angles are observed, corresponding to the latter half of the $|33\rangle \to |11\rangle$ branch. In this way, we successfully capture the evolution from $|11\rangle$ to $|00\rangle$ and $|33\rangle$ to $|11\rangle$.

To validate that we are tracking a single mode, we show the lasing spectra of samples $\mathcal{U}$ through $\mathcal{Z}$ over a broad wavelength range of approximately 200 nm (from 1450 nm to 1650 nm) in Fig. R6. Each sample exhibits stable single-mode lasing,indicating that the intrinsic mode selectivity in the lasing scheme is sufficient to maintain single-mode operation throughout the entire evolution trajectory. Similar single-mode behavior is observed for the C_2-symmetry-broken samples (Fig. R7), further supporting the robustness of the mode selection mechanism. We have added these discussions and spectral data to the Supplementary Material (Section 9, "Design of PhC for lasing").

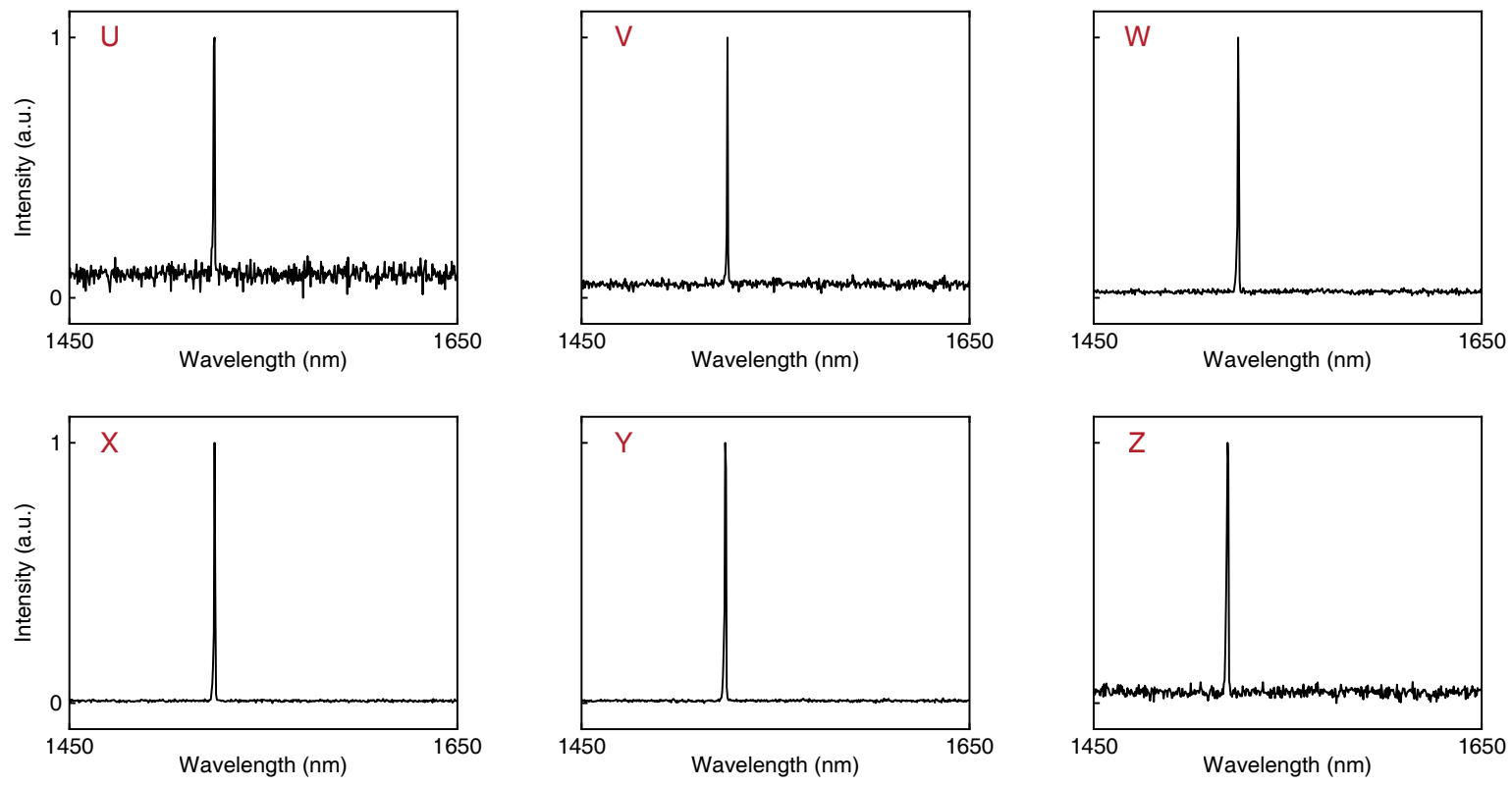

Figure R6 **Figure S10 in the Supplementary Material.** Measured lasing spectra for sample $\mathcal{X}$ through $\mathcal{Z}$ with circular holes.

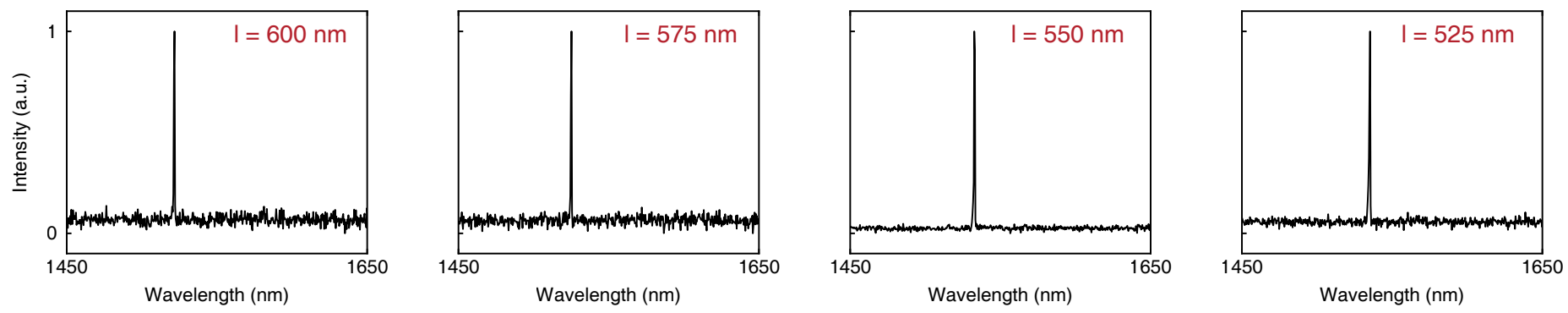

Figure R7 **Figure S11 in the Supplementary Material.** Measured lasing spectra for samples with irregular-shaped air holes.

COMMENT 1.E

> Third, the experimentally measured spatial mode profiles do not appear to closely match the simulated ones. More importantly, the main objective of the paper is to demonstrate the formation of a flat-top spatial mode, yet the mode shown in Figure 4c ($\mathcal{Z}$) exhibits essentially zero field intensity in the central region. Instead, the experimental data consistently show that the optical intensity is concentrated at the edges of the structure, i.e., near the boundaries. It is not obvious that such a mode is of practical interest from a device perspective. Indeed, the far-field emission is dominated by the edge emission and displays a diffraction pattern that is typically undesirable in photonic devices.

REPLY 1.E

We sincerely thank the reviewer for this important comment. It reminds us that there may be some confusion about the modal envelope profile (MEP) calculated by theory and simulation, and the experimentally observed near-field pattern (NFP).

We clarify that these two quantities (MEP and NFP) are distinct in physics (Fig. R8): the MEP represents the slowly varying envelope of the full modal field distribution. In contrast, the NFP corresponds to the patterns of out-of-plane emission imaged at the upper-surface of the laser, which is determined by both the MEP and the radiation rate at each position. In the experiment, only the radiative component, not the full field, emits as the lasing beam. Therefore, the NFP reflects a combined effect of the modal envelope modulated by the radiative rate.

Specifically, for a mode in the PhC cavity, its electric fields can be expressed as:

$$E(r) = F(x, y) \sum_{m,n} E_{m,n}(z) e^{iG_m x + iG_n y} \tag{R8}$$

where $G_{m,n}$ are reciprocal vectors and m, n are integers denoting its order; $E_{m,n}(z)$

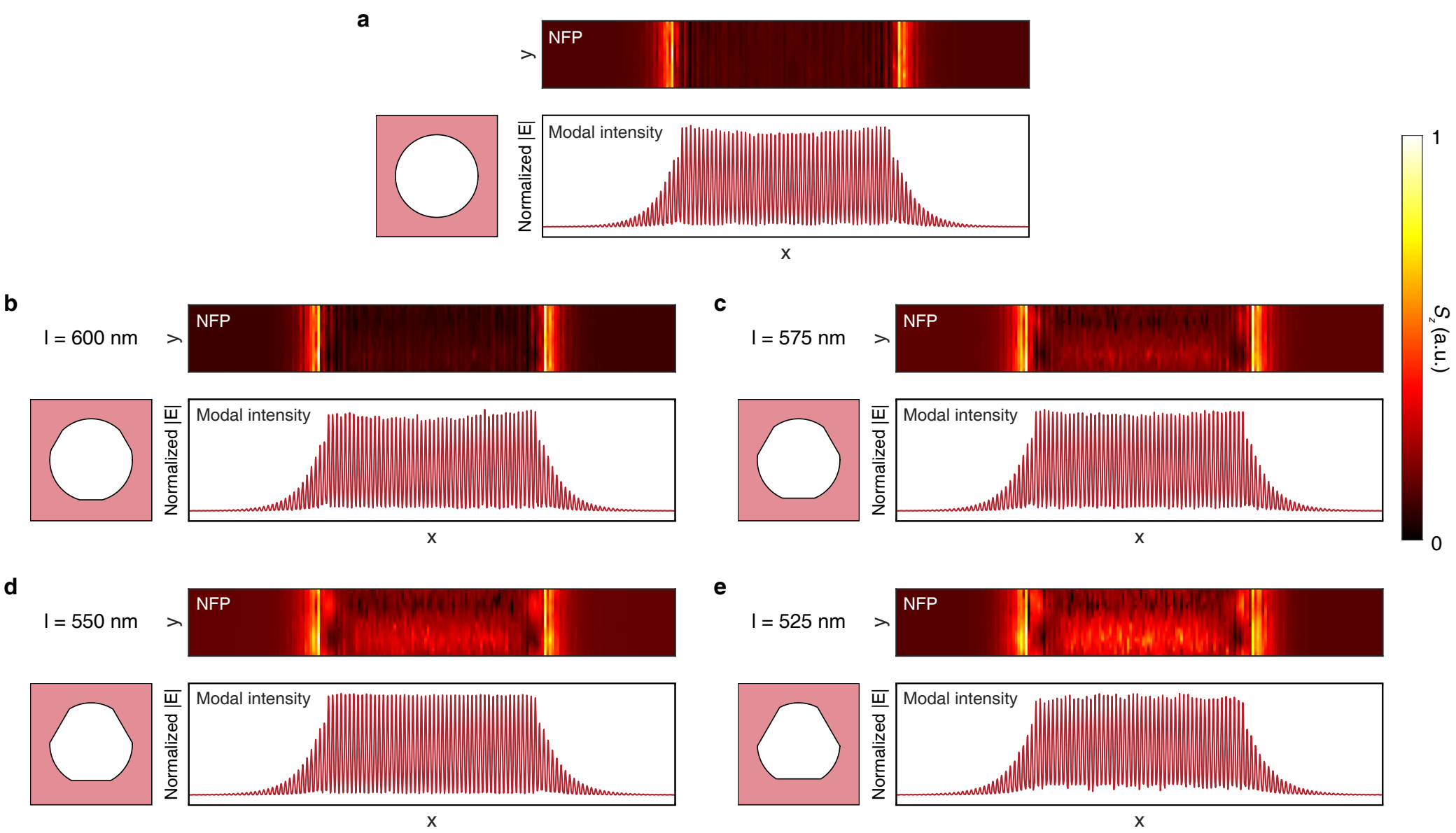

Figure R8 **Figure S12 in the Supplementary Material.** Achieving a bright flat NFP by increasing the degree of C_2-symmetry breaking.

is the profile of each Bloch component in z direction; $F(x, y)$ is in-plane slowly varying envelope. Obviously, the electric field $E(x, y, z = z_{\text{surface}})$ includes both radiative component ($|G_{m,n}| < \omega/c$) and non-radiative components ($|G_{m,n}| > \omega/c$). By removing the fast-oscillating part, we can extract the slowly varying envelope $F(x, y)$, as shown in Fig. 2d in the main text.

Note that those non-radiative field components are evanescently decaying in the z direction and do not contribute to the lasing emission. As a result, the NFP only captures the out-of-plane radiation. Moreover, the flat-top $|00\rangle$ mode duplicates the radiation characteristics of the band edge to the whole cavity. Accordingly, the NFP of $|00\rangle$ mode after lasing can be expressed as $\gamma_\Gamma F(x, y)$, where γ_Γ denotes the radiative rate at the Γ point. As for Fig. 4 in the main text, we consider a Bloch band with circular air-holes on a square lattice, which naturally supports a BIC right at the Γ point that eliminates the radiation ($\gamma_\Gamma = 0$). Consequently, the central cavity region is quite dark in the observed NFP. To sum up, the central region darkness on the NFP of sample $\mathcal{Z}$ does not indicate that the modal envelope is zero in the bulk, but originates from the zero radiation at the band edge.

Following the same principle, we also note that the appearance of boundary scattering on the NFP does not necessarily indicate that the cavity mode concentrates its field at the bulk-boundary interface. In fact, these scatterings arise from momentum mismatch at the interface because we introduce a gap for phase accumulation. We found that these scatterings do not affect the in-plane phase engineering, as well as the formation of the $|00\rangle$ mode. As exemplarily shown in Fig. R8a, we consider a simplified 3D PhC cavity with circular holes. By optimizing the boundary phase shift, we can realize the $|00\rangle$ mode with a flat-top-like modal envelope (bottom panel). However. a dark central region can be found in the corresponding NFP, while scatterings emerge at the bulk-boundary interfaces. These simulation results match well with our experimental results shown in Fig. 4c in the main text (sample $\mathcal{Z}$), indicating that the NFP indeed is different from the MEP.

We understand that the reviewer's concern that the observed NFPs with a dark central region and edge scatterings may be undesirable for practical applications. We hereby propose two methods for this issue:

First, we can make the central region brighter by combining the $|00\rangle$ mode with a quasi-BIC. In literature, double-holes or irregular-shaped holes are utilized to break the C_2 symmetry and turn the BICs into quasi-BICs [R15]. In our case, this means the central region gains nonzero radiative rates γ_Γ. As shown in Fig. R8b-e, we make simulations on a series of designs that gradually change the circular holes into irregular holes with different asymmetric parameter l (smaller l corresponds to stronger C_2 breaking). For each design, we finetune the boundary phase to reach the $|00\rangle$ modes with flat-top envelopes (bottom panels). We found that, with the C_2 breaking, the central region in NFPs becomes brighter, even comparable to the interface scatterings. In this way, we can obtain a near-flat NFP.

We fabricated new samples to demonstrate this bright near-flat NFPs, shown in Fig. 5 in the main text (Fig. R9 here). Specifically, the samples have different l values, and the NFPs and their cut-lines along x direction are measured, as shown in Fig. R9c. The NFPs (top panels) are normalized to the intensity of interface scatterings, and the cut-lines (bottom panels) are obtained by integrating the NFP strength in the y direction. As l decreases (stronger C_2 breaking), the bulk region in NFPs gradually becomes brighter, showing a prominent increase in the emission intensity within the bulk region. These experimental observations are in excellent agreement with our 3D simulations (Fig. R8), confirming that controlled asymmetry lowers the radiative Qs and makes vertical radiation from the bulk comparable to or even stronger than the edge scatterings, eventually giving rise to a flat NFP.

Second, we can suppress the boundary scatterings by adopting a metallic mirror rather than a photonic band gap, since such a hard boundary can eliminate momentum mismatch. The reflection phase can also be tuned by adjusting the gap between the PhC region and the metal mirror. However, this design may be more challenging to fabricate, as it requires a high-quality metallic side mirror with precise alignment and minimal optical loss. For this reason, we do not pursue this approach experimentally, although it remains a promising direction for platforms where metallic boundaries can be more readily integrated.

To avoid any misunderstanding, we add some discussions on page 12-14 in the main text to address the distinction between the NFP and MEP. Further, we update the Fig. 5 in the main text with new experiment results shown in Fig. R9, to directly demonstrate the quasi-BIC lasing in $|00\rangle$ mode and the formation of near-flat NFP.

We also add Section 10, “Interpreting near-field patterns and realizing uniform out-of-plane emission”, in the Supplementary Material to discuss practical routes toward device-ready flat-top emission.

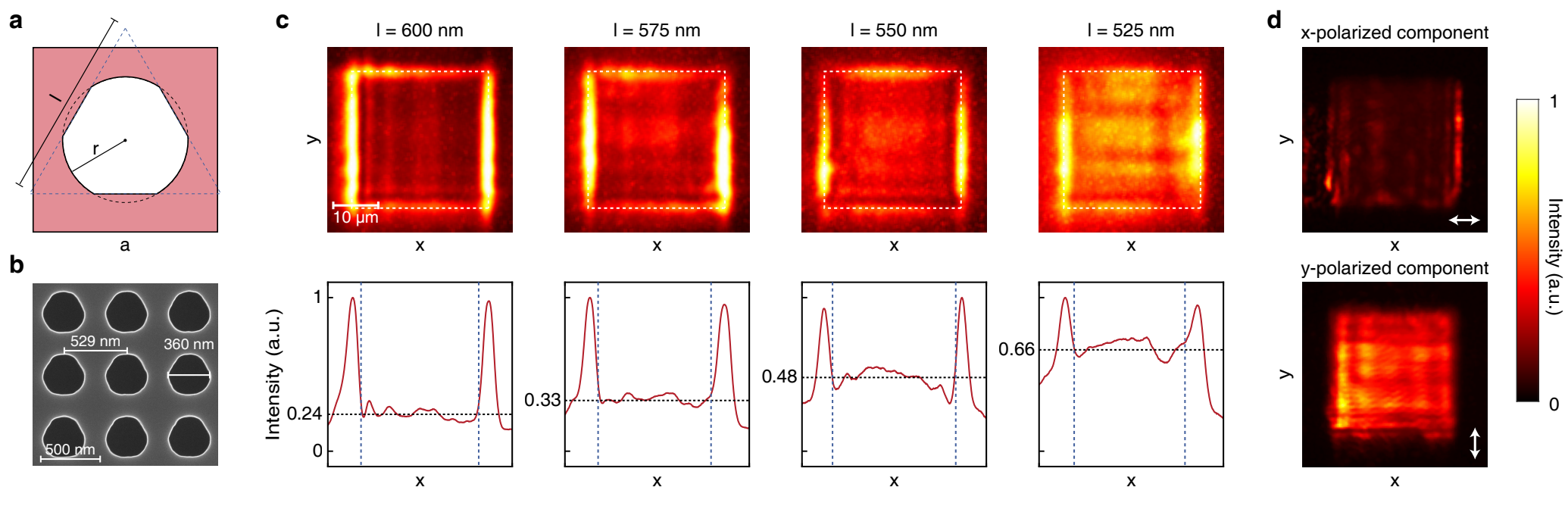

Figure R9 **Figure 5 in the main text.** Near-flat-top fundamental state in C_2-symmetry-broken system.

COMMENT 1.F

> The authors attribute the absence of intensity in the central region to the formation of a bound state in the continuum (BIC) that suppresses radiation losses. However, the physical mechanism leading to the formation of this BIC is not clearly explained. Moreover, to substantiate the claim of a BIC, additional experimental evidence should be provided. For instance, measurements of the quality factor or a systematic study of the laser threshold, as the system approaches the supposed BIC condition, would significantly strengthen the manuscript.

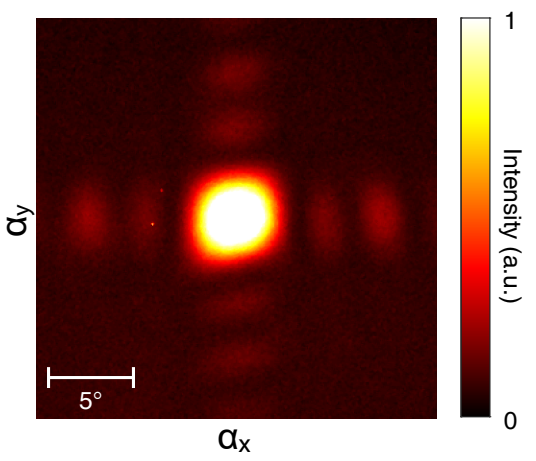

Figure R10 Far-field distribution of $|00\rangle$ state with irregular-shaped holes.

REPLY 1.F

We thank the reviewer for this constructive comment. As mentioned in our reply to the last **comment** 1.E, a BIC resides at the Γ point of the Bloch band we're focused, which prevents the vertical radiation ($Q_\Gamma = \infty$) and thus contributes to the dark region in NFP. The physics of this BIC is very straightforward: it is protected by the C_4 symmetry in circular-hole design. As shown in Fig. 3d in the main text and Fig. S9a in the Supplementary Material, the Q factor of this Bloch band diverges right at the Γ point, indicating the existence of a symmetry-protected BIC.

The existence of the BIC can be indirectly proven by various experimental phenomena. First, in Fig. 4 in the main text, the FFP of the lasing beam is donut-shaped as a typical feature of the BIC lasing [R16]. Also, when we break the C_2 symmetry by employing the irregular-shaped holes, the donut-shaped FFP is replaced by a single lobe, shown in Fig. R10, evidencing the transition from the BIC to a quasi-BIC.

We agree that measuring the Q or lasing threshold could, in principle, serve as evidence for a BIC. However, in our hetero-bonded cavity, the total Q is contributed by both radiative and non-radiative losses, while only the radiative loss can be suppressed by the BICs. Due to the existence of considerable material absorption and scattering losses, the total Q may be a less accurate indicator of the BICs.

Instead, we provide a direct and definitive experimental signature for the BIC: the polarization vortex in the lasing beam's FFP, which is a hallmark of a BIC at the Γ point. We have performed polarization-resolved far-field measurements for sample $\mathcal{Z}$ (the near-$|00\rangle$ state). As shown in Fig. R11, by inserting a linear polarizer and rotating it, the original donut-shaped main lobe in FFP (highlighted in dashed box) becomes two lobes, and rotates accordingly with the polarizer, evidencing a nonzero topological charge that unambiguously indicates a polarization vortex. Since our samples are on a simple square lattice with C_4 symmetry, such a polarization vortex can only be attributed to far-field characteristics of the BIC, directly confirming its existence at Γ point without relying on low-fidelity Q measurements.

We hope these additional evidences satisfactorily address the reviewer's concern.

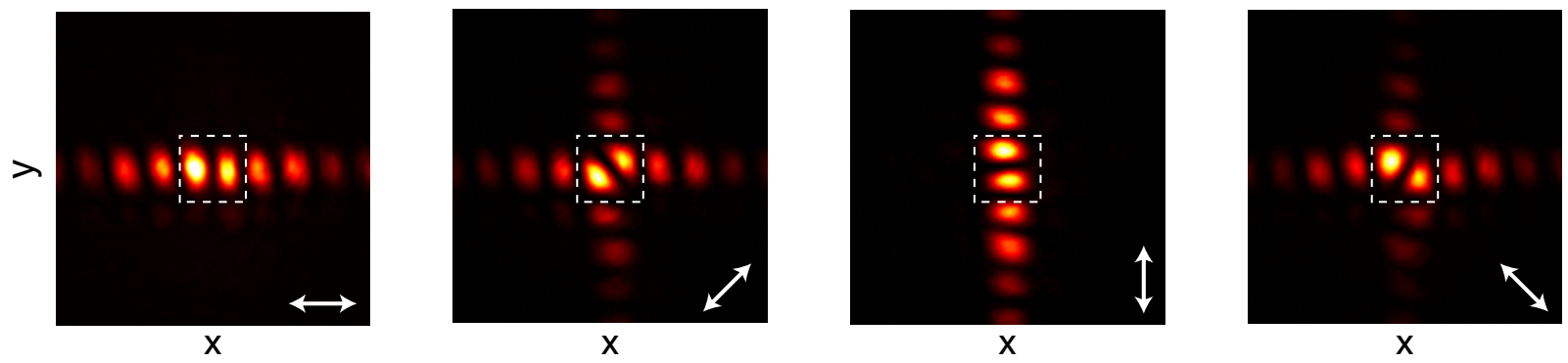

Figure R11 Polarization-resolved far-field distribution of $|00\rangle$ mode.

COMMENT 1.G

In conclusion, while the manuscript presents an interesting and original theoretical idea supported by convincing numerical simulations, the experimental results are not enough to validate the proposed concept. The central claim of realising a BIC flat-top spatial mode is not convincingly demonstrated, and the experimental observations raise doubts about the physical interpretation, particularly regarding mode selection and the claim of a bound state in the continuum. Key aspects of the experimental design, parameter space, and supporting evidence remain insufficiently explained. Given the gap between the theoretical proposal and the experimental validation, I do not believe that the present work meets the level of completeness and impact required for publication in Nature Communications. I therefore do not recommend the manuscript for publication.

REPLY 1.G

We would like to gratefully thank the reviewer for his/her careful assessment and insightful comments regarding the completeness of the experimental validation and interpretation of our theoretical claims. We fully appreciate the reviewer's concerns and thank you for the opportunity for clarification. In supplement to the detailed point-by-point responses above, we also briefly summarize the key points as follows:

(i) The goal of this work is to present the physics on controlled morphing of spatial envelopes in finite wave systems through phase engineering. We showed that the gauge picture provides a general principle, suggesting that the root origin of flat-top-like envelopes is an appropriate phase accumulation, driven by the effective gauge potential.

(ii) We clarified the differences between the 2D model for simulation and 3D structure for experiments. We fabricated new samples and provided additional data, confirming the observed envelope evolution on parameter g is consistent with our theory.

(iii) We elaborated on the details of the mode selection in the lasing scheme, and confirm that all the samples we measured operate in single-mode lasing.

(iv) We clarified the difference between NFP and modal envelope, and explained the reason for the central darkness and boundary scatterings on the NFP. We also proposed several methods to overcome the scattering and realize the near-flat NFP.

(v) We have clarified the formation of the symmetry-protected BIC in our design and measured the far-field polarization vortex of the lasing beam as evidence.

We further revised the manuscript to improve the clarity of the experimental description, strengthened the connection between theory and experiment, and clarified potential ambiguities in the presentation.

Taken together, we believe that the combination of theoretical analysis, numerical validation and experimental observations provides consistent and sufficient support for the central concept of phase-engineered envelope morphing in this work. We hope that these clarifications and revisions resolve the reviewer's concerns and demonstrate that the work meets the level of completeness and significance required for publication.

Reply to Referee 2

COMMENT 2.A

> This manuscript reports an interesting and compelling experimental and theoretical study on wave morphing toward a flat-top ground state in laterally confined photonic crystal slabs, enabled by an artificial gauge field implemented via boundary reflective phases. By invoking the Byers–Yang theorem and mapping the system to an effective ring model, the authors demonstrate continuous energy-level shifting and experimentally probe the eigenstate evolution through lasing emission. The work is technically strong, clearly presented, and experimentally impressive. Thus, I recommend acceptance of this manuscript after the authors clarify some conceptual and interpretational points to further strengthen the manuscript.

REPLY 2.A

> We sincerely thank the reviewer for the positive assessment and for the constructive suggestions to further strengthen the manuscript. We hereby provide a point-by-point responses for all the comments list below.

COMMENT 2.B

> A central result is the observation of lasing modes approaching a near-flat-top ground state (notably sample $\mathcal{Z}$). While this is an intriguing manifestation of gauge-field-induced wave morphing, its practical relevance for lasing applications remains somewhat ambiguous. In particular, a flat-top ground state corresponds to vanishing in-plane wavevector and isotropically suppressed group velocity, closely resembling a band-edge or frozen mode. Such states typically suffer from weak mode selectivity and may be unfavorable for robust, high-quality single-mode lasing once issues such as gain competition and saturation are considered. Thus, the authors may wish to clarify: whether the flat-top ground state is envisioned primarily as a linear-wave or passive eigenstate, rather than a practical lasing mode; or whether specific gain, loss, or symmetry-breaking mechanisms (beyond those briefly discussed in Fig. 5) could stabilize such a mode for lasing.
>
> A short discussion acknowledging these limitations would improve the physical interpretation and avoid overextending the technological claims.

REPLY 2.B

> We thank the reviewer for this insightful comment. As the reviewer pointed out, the primary goal of this work is to provide a proof-of-principle demonstration of a new physical mechanism, namely, envelope morphing toward a near-flat-top envelope enabled by an artificial gauge field, rather than to develop an optimized laser architecture. At the same time, we fully agree that, in principle, the practical lasing application based on flat-top modes remains somewhat ambiguous. Hereby we would like to give a short discussion upon the potentials and limitations of flat-top modes in lasing scheme.
>
> First, we would emphasize that in our current experiments, all fabricated devices, including those operating close to the flat-top mode (e.g., sample $\mathcal{Z}$), exhibit stable single-mode lasing, as evidenced by the spectra presented in the main text and Supplementary Material (Section 9, "Design of PhC for lasing"). This indicates that, within the scope of our proof-of-principle demonstration, mode selectivity is not compromised. The observed single-mode operation can be attributed to the relatively high quality factor (Q) of the lowest-order transverse mode, together with its favorable spatial overlap with the pumped gain region.
>
> Specifically, the flat-top mode $|00\rangle$ possesses the highest Q among all cavity modes under the same phase-engineering condition, and therefore dominates the lasing

process. On one hand, the effective in-plane wavevector of the flat-top state approaches zero, placing it closest to the band edge, where a BIC or quasi-BIC can suppress vertical radiation. As a result, it exhibits the highest radiative Q. On the other hand, the presence of the photonic bandgap suppresses lateral leakage, further enhancing its lateral confinement. In addition, we note that when the engineered boundary phase shift approaches $\theta \sim \pi$, interface scattering at the bulk-boundary interface becomes pronounced for all modes. The higher-order cavity modes are generally more susceptible to such scattering due to their larger spatial extent and more complex field distributions. Taken together, these factors ensure that the flat-top mode maintains the highest effective Q among all the competing modes, thereby supporting stable single-mode operation in our system.

However, in practical electrically-pumped devices, additional considerations arise. In our experiments, optical pumping with a large spot size ensures an approximately uniform gain distribution across the entire cavity, which is favorable for the excitation of flat-top modes. In contrast, in electrically injected lasers, carrier diffusion and non-uniform current injection typically lead to a spatially non-uniform gain profile. Such non-uniformity may partially compromise the formation and stability of flat-top modes. Specifically, a spatially varying gain can distort the uniform intensity distribution of the flat-top mode, leading to deviations from the ideal plateau-like envelope. Moreover, a non-uniform gain distribution may favor other modes with better local gain overlap. As a result, the flat-top mode may no longer dominate the lasing process.

To address this problem, the current injection profile should be engineered to recover a more uniform gain distribution such that the flat-top mode can preserve its advantages under electrical pumping. In literature, injection gain profile engineering has been discussed and investigated in-depth [R17]. We believe that exploring such corporation between cavity phase engineering and gain engineering would be an important direction for realizing practical devices based on flat-top modes.

COMMENT 2.C

The manuscript briefly connects the observed flat-top states to non-Hermitian physics and mentions skin-effect-induced localization. However, I assume the flat-top ground states demonstrated here are qualitatively different from conventional non-Hermitian skin modes. Although the authors correctly note that non-Hermiticity becomes relevant when boundary leakage is present, the relationship between these two phenomena could be articulated more clearly. It would be helpful if the authors can clarify this explicitly: the flat-top distributions are not skin modes in the usual sense, and they arise from gauge-field-induced spectral shifting and effective Neumann boundary conditions rather than non-reciprocal pumping?

Such clarification would help place the work more precisely within the broader non-Hermitian photonics literature.

REPLY 2.C

We thank the reviewer for this insightful comment and helpful suggestions. We fully agree that the flat-top modes demonstrated in our work is fundamentally distinct from the non-Hermitian skin modes, even with a leaky boundary condition. We apologize for the inappropriate phrases on the flat-top-like modes under non-Hermitian boundary conditions. In the revised manuscript, we have clarified this point and now provide a careful discussion to distinguish these two phenomena.

For flat-top modes, the boundary phase shift or effective Neumann boundary conditions we employed serve as an artificial gauge potential, giving rise to a vanishing slowly varying wave vectors and thus a uniform, nonzero field distribution. When the reflection at the bulk-boundary interface becomes imperfect, the slowly varying wave vectors

become purely imaginary under a proper gauge transformation, giving rise to a boundary-enhanced envelope that makes the modal intensity concentrate around the interface. In these cases, only the envelope is engineered, while the fast oscillating Bloch waves are not affected. Most importantly, the system is always reciprocal: the coupling from R component to S component is always conjugated with the coupling vice versa.

In contrast, the non-Hermitian skin effect (NHSE) typically originates from non-reciprocal hopping (e.g., asymmetric intracell hopping in a non-Hermitian SSH chain), which forces all bulk states to localize at one boundary. In NHSE scheme, the Bloch model becomes invalid and a non-Bloch theory should be employed. The non-Hermiticity in our design arises from energy leakage at the boundaries, while in NHSE, it typically refers to the non-reciprocity. Therefore, the proposed boundary-enhanced envelope in this work fundamentally differs from the NHSE.

We have clarified this distinction in the main text and provided a more detailed discussion in Section 8, "Non-Hermitian ring model", of the Supplementary Material.

COMMENT 2.D

The discussion of bound states in the continuum (BICs) is appropriate and well supported, particularly in connection with band-edge lasing and radiation suppression near the $|00\rangle$ state. However, recent studies have shown that higher-order topological phases can also host BICs (for instance, Cerjan et al., Phys. Rev. Lett. 125, 213901, 2020; Hu, et al. Light Sci Appl , 10, 164, 2021), including corner- or hinge-localized BICs protected by crystalline symmetries. Given that the present system already relies on symmetry, boundary engineering, and band topology, the authors may consider briefly commenting on: whether higher-order topological BICs could be realized in similar heterogeneous photonic crystal geometries; or whether such modes could coexist or compete with the flat-top ground state demonstrated here.

Even a short qualitative remark and one or two representative citations would broaden the conceptual scope of the work.

REPLY 2.D

We thank the reviewer for this insightful comment. In fact, in our PhC cavity, the BIC originates from symmetry protection, namely, it is a bound state residing inside the light cone but decouples from the radiation continuum. Therefore, this kind of BICs can coexist with the flat-top modes, since they actually deal with different physics: BICs in our system refer to Bloch modes which decouple from the radiation continuum and thus have infinite radiative Q; while the flat-top modes refer to transverse cavity modes with flat, uniform spatial envelop.

In our work, the BIC is utilized to shape the radiation characteristics of flat-top modes. Specifically, by creating transverse modes in a Bloch band with a BIC right at the band edge and employing proper boundary phase shift, a flat-top mode with radiation suppressed by the BIC is achieved, giving rise to a dark NFP and a polarization vortex in FFP. Further, by introducing C_2 asymmetry, we transit the BIC into a quasi-BIC with nonzero radiation, and thus a quasi-BIC lasing based on such a flat-top mode is realized, possessing a near-flat NFP (Fig. 5 in the main text).

However, the high-order topological bound states in the continuum, the reviewer mentioned above, are quite different. It refers to topological corner modes in high-order topological insulators (HOTIs) which does not hybridize with the bulk bands, even though their frequencies are degenerate with each other. These corner modes are formed by nontrivial higher-order topological phases, and become bound states when spatially trapped by a proper bulk band-gap provided by crystalline symmetries. Therefore, high-order topological BICs cannot be directly realized in the heterogeneous

photonic-crystal cavity used in this work, as our design does not support a topologically non-trivial phase. However, we believe that flat-top modes can coexist with high-order topological BICs under appropriate conditions.

The fundamental mechanism behind flat-top modes is the gauge-induced phase engineering, where spatial permittivity modulation acts as an effective gauge potential. This approach is not limited to topologically trivial phases and can be extended to topologically non-trivial wave systems. Although in this work, phase engineering is primarily used to manipulate the envelope of truncated bulk modes, it can also be applied to engineer the profile of edge states, including topological defect modes or corner modes, as these states typically have tails that extend into the bulk. Specifically, by applying an adiabatic modulation to the bulk in topological lattice without inducing a topological phase transition, a phase gradient can be introduced to simply manipulate the localization profiles of topologically-protected BICs, reshaping them into flat-top-like envelopes. The integration of spatial modulation with the non-trivial topological phase can combine the advantages of both flexible envelope control and the robustness of topological protection.

Thus, while our current design does not directly support topologically non-trivial phases, we believe that the principle of gauge-induced envelope manipulation can be extended to topological systems. This would enable the coexistence of flat-top modes and high-order topological BICs in more advanced photonic-crystal designs. We have added comments in the conclusion section of the manuscript to discuss the potential application of the proposed gauge-induced envelope morphing strategy in general wave systems, including those with topologically non-trivial phases.

COMMENT 2.E

Some fonts in figures seem to be too small without zoom-in for clear visualization.

REPLY 2.E

Thank you for pointing this out. We have carefully adjusted the font sizes in all figures. Specifically, we have revised Figures 3-5 and other relevant panels in the main text to improve the balance between text and graphics, ensuring that all labels and annotations are clearly legible without zooming.

COMMENT 2.F

Overall, this is a strong and elegant contribution, combining clear theoretical insight with convincing experimental validation. Addressing the points above would mainly improve conceptual clarity and positioning, rather than requiring new experiments. I recommend publication after minor revision.

REPLY 2.F

We sincerely thank the reviewer for the positive evaluation of our work and for the constructive suggestions that helped improve the clarity and presentation of our manuscript. We are very encouraged by the reviewer's assessment that the work combines clear theoretical insight with convincing experimental validation.

Following the reviewer's comments, we have carefully revised the manuscript. In particular, we have further clarified the central physical claims, strengthened the conceptual discussion of the phase-engineering picture, and improved the overall positioning of the work to avoid potential ambiguities. We have also discussed the mode selectivity of proposed flat-top mode, and revised several expressions to better reflect the physical interpretation and to improve readability. We believe that the revised manuscript has been significantly improved accordingly.

Reply to Referee 3

COMMENT 3.A

> The manuscript presents a general mechanism for tuning energy levels and wave morphology via geometric phase, and demonstrates, both theoretically and experimentally, the controlled morphing of optical waves into a flat-top ground state via an artificial gauge field. By engineering boundary reflections, a spatially uniform, non-zero ground state can be realized in a finite, confined system. That is counterintuitive and opens new avenues for light manipulation. I find this present work integrates the core concept of gauge field theory from condensed matter physics with the authors' prior researches (including BIC modes, heterogeneous integration techniques, and optical field manipulation), thereby establishing a novel research paradigm.
>
> All in all, this work is conceptually novel, technically sound, and represents a significant contribution to the fields of photonics, wave physics, and quantum simulation. The manuscript is, also, well and clearly written, even for non-specialist readers, and minor suggestions and comments include:

REPLY 3.A

> We sincerely thank the reviewer for the thoughtful and positive assessment of our work, and for recognizing the integration of gauge field concepts with our previous research to establish a new paradigm.

COMMENT 3.B

> Figure 2c and the experimental section only present the numerical values of g and the corresponding phenomena. The values of θ (e.g., $\theta = \pi$) appear to be derived from simulation or theoretical inference. Readers cannot ascertain, for instance, how θ increases linearly or nonlinearly from 0 to π as g changes from 565 nm to 474 nm. Other researchers may be confused about accurately predicting the gauge field state of their system when given a specific value of g.

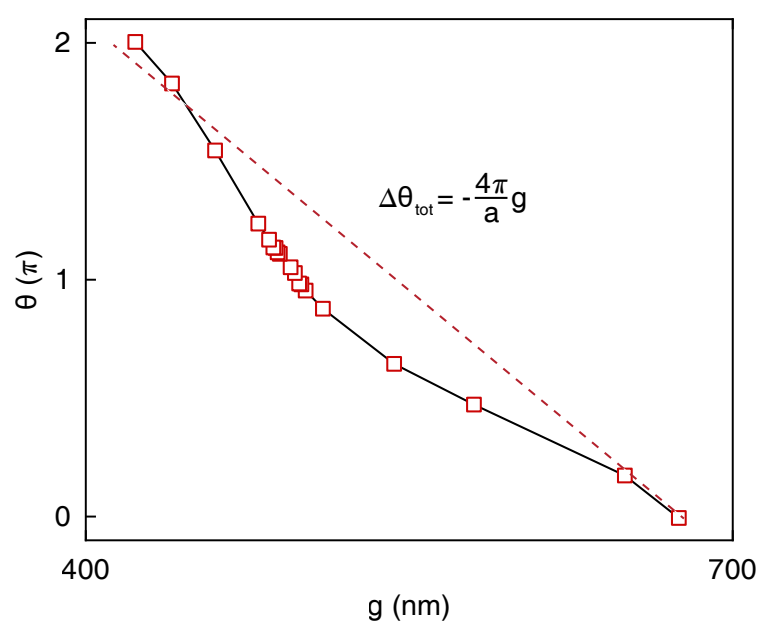

Figure R12 **Figure S7 in the Supplementary Material.** Reflection phase θ as a function of the spacing g. The solid dotted curve corresponds to simulation results, and the dashed line represents the theoretical prediction assuming θ_R remains unchanged as g varies.

REPLY 3.B

> We thank the reviewer for this constructive suggestion. We agree that a direct theoretical relationship between the gap g and reflection phase θ would be helpful for readers to better understand our work.
>
> As we presented in Supplementary Material Section 4, "Coupled-wave theory for homogeneous 2D PhC slab", in theory, we calculate the relative phase θ through the coupled-wave theory (CWT) [R18–R20]. In CWT, the transverse modes can be considered as a superposition of varieties of Bloch components with engineered

relative phases. With proper boundary conditions with a gap of g, we can calculate the couplings between Bloch components and the boundary phase shift θ is naturally derived. By using this method, we have performed systematic 2D simulations using the heterogeneous PhC model (the same as the one used in Fig. 2c) to compute θ as a function of g (Fig. R12), showing a monotonic increase of θ from 0 to 2π as g decreases from 675 nm to 423 nm.

Nevertheless, the CWT is a complicated semi-analytical theory and it's hard to derive a closed form of $\theta(g)$ directly from it. Instead, we present a simple theoretical model here. Specifically, the flat-top-like transverse modes around the 2nd-Γ point can be considered as a superposition of only two counter-propagating Bloch components Re^{ikx} and Se^{-ikx}, where $k \approx |G| = 2\pi/a$ holds since the slowly varying wave vectors of envelope vanish for flat-top-like modes. G is the reciprocal vector and a is the crystal constant. These two components dominate the energy of the transverse modes so the high-order components are omitted for simplicity. The relative phase between R and S components can be simply concluded as follows:

$$\theta_{\text{tot}} = \theta_R + \theta_D = \theta_R - 2GL = \theta_R - \frac{4\pi}{a}L \tag{R9}$$

where θ_R is the phase shift induced directly by the reflection of photonic-band gap. It is determined from varieties of factors, including the width of photonic-band gap, the material permittivity, the group velocity of the relevant Bloch band, and so on. For a given design with only bulk-boundary gap g varied, we first assume θ_R to be constant and simply serves as a bias.

The second term, $\theta_D = -2GL$, is the dynamical phase accumulated during propagation. Specifically, we assume $L = Na + 2g$, where N is the cell number of the bulk region. In this way the dynamic phase can be divided into two parts: $\theta_D = \theta_f + 2\theta_g$. The first part $\theta_f = -4N\pi$ attributes to the fast oscillation, and has no contribution to the slowly varying envelope shaping. The second part is two times of extra phase accumulated in an interface gap g:

$$\theta_g = -\frac{4\pi}{a}g \tag{R10}$$

This part plays a crucial role for the boundary phase engineering. By altering the gap g, extra phase accumulation would be obtained and contributes to the reflection phase θ: $\Delta\theta = \Delta\theta_g$. For variance of half a crystal constant $\Delta g \approx a/2$, an additional phase shift of $\Delta\theta = -2\pi$ could be achieved. In our design, the crystal constant is set to be $a = 529$ nm. And according to Fig. R12, by turning g from 675 nm to 423 nm, corresponding to $\Delta g = 252$ nm $\approx a/2$, a boundary phase shift $\Delta\theta = 2\pi$ is obtained, matching well with the theoretical prediction.

By assuming θ_R is unchanged as g varies, we plot the theoretical prediction of θ with respect to g according to Eq.R10, shown as a dashed line in Fig. R12. From the simplified model, θ shows a linear dependence on g. However, in simulations dealing with a realistic cavity, $\theta(g)$ curve is not linear, but twists when θ approaches π. The distinction mainly comes from the fact that θ_R actually varies with respect to g. For example, when approaching to the flat-top condition ($\theta \approx \pi$), the slowly varying wave vector of the fundamental mode vanishes, corresponding to the band edge, and thus the group velocity also vanishes. As a result, the direct reflection θ_R changes accordingly.

The rigorous CWT, together with the simplified prediction in Eq. R10, gives the theoretical model of effective boundary phase shift θ with respect to interface gap g. The relevant theory has been elaborated in the Supplementary Material Section 7, "Theoretical model of $\theta - g$ relationship". We hope these discussions could help a better understanding of this work.

COMMENT 3.C

> The identification of the "near-flat-top state" in Fig. 4 relies heavily on the minimal divergence angle (1.2°), but no quantitative metric for absolute field uniformity(e.g., intensity variance) is provided. The statement that "the bulk region appears almost dark" raises the question: does this imply energy is primarily confined at the boundary? If so, how does this align precisely with the definition of a "flat-top" state distributed throughout the bulk?

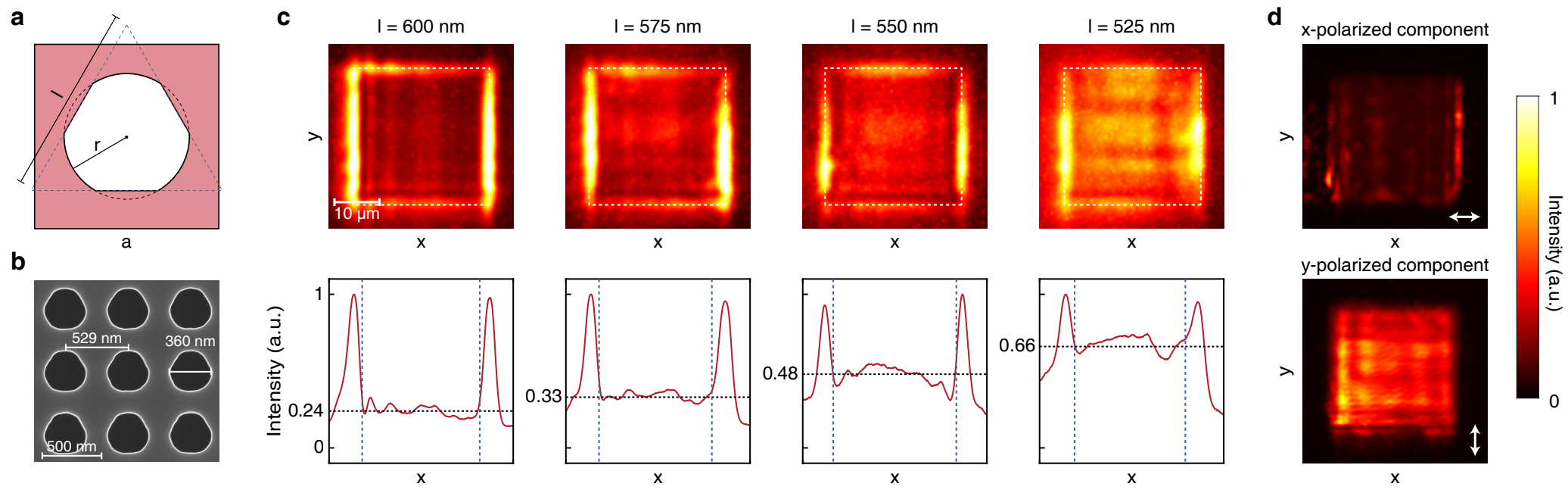

Figure R13 **Figure 5 in the main text.** Near-flat-top state in C_2-symmetry-broken system.

REPLY 3.C

> We thank the reviewer for these important comments. We fully agree that a direct quantitative metric for absolute field uniformity would help to validate the near-flat-top mode. As discussed in our reply to **comment 1.E**, the modal envelope profile (MEP) is not observable through the microscopic set-up, and only the near-field patterns (NFPs) of the lasing beam could be captured in experiments, which show a combined effect of intrinsic MEP and radiative rate. As a simple summary, the NFP alone is not a direct measurement of the modal envelope, unless the radiative rate γ_Γ is a nonzero constant.
>
> For sample $\mathcal{Z}$ shown in Fig. 4c in the main text, a symmetry-protected BIC resides at the band edge, suppressing the vertical radiation ($\gamma_\Gamma = 0$). As a result, the lasing emission from the central bulk region is nearly prohibited, giving rise to the darkness in its NFP. On the other hand, the interface scattering due to momentum mismatch stands out, giving rise to a boundary-enhanced brightness shown in Fig. 4. Note that the bulk region in NFP appears almost dark, which does not imply that energy is primarily confined at the boundary; rather, it indicates that out-of-plane radiation from the bulk is strongly suppressed due to the presence of a BIC at the band edge. This point has been elaborated in the Supplementary Material Section 10, "Interpreting near-field patterns and realizing uniform out-of-plane emission".
>
> To make the central region bright, we applied controlled C_2 symmetry breaking using irregularly shaped air holes (Fig. 5 in the main text, and Fig. R13 here). The C_2 breaking turns the BIC into a quasi-BIC with nonzero radiation, and thus gives rise to a nonzero constant γ_Γ. In this way, the NFP can truly represent the modal envelope profile (MEP).
>
> We fabricated new samples with different l values, where smaller l corresponds to stronger C_2 breaking. For every sample, boundary phase shift is fine-tuned to achieve the flat-top modes. The measured NFPs (top panels) are normalized to the interface scattering. For all four samples, the NFPs are almost flat, and the bulk region becomes progressively brighter as l decreases. The cut-line of the NFP along x direction in bottom panels quantify this trend, demonstrating a direct measurement of the uniform modal envelope along x direction.
>
> Moreover, we calculate the intensity variance of the normalized NFP across the whole central bulk region for these four samples, compared to that of sample $\mathcal{X}$ which

represents the $|11\rangle$ state. The intensity variance is 0.0018, 0.0033, 0.0037, and 0.0061, respectively, significantly lower than that of sample $\mathcal{X}$ (variance of 0.0409) by one order of magnitude, providing a direct quantitative metric of envelope uniformity. This experimental quantification, which is in excellent agreement with our 3D simulations (Supplementary Fig. S12, and Fig. R14 here), and supports our identification of the near-flat-top modes.

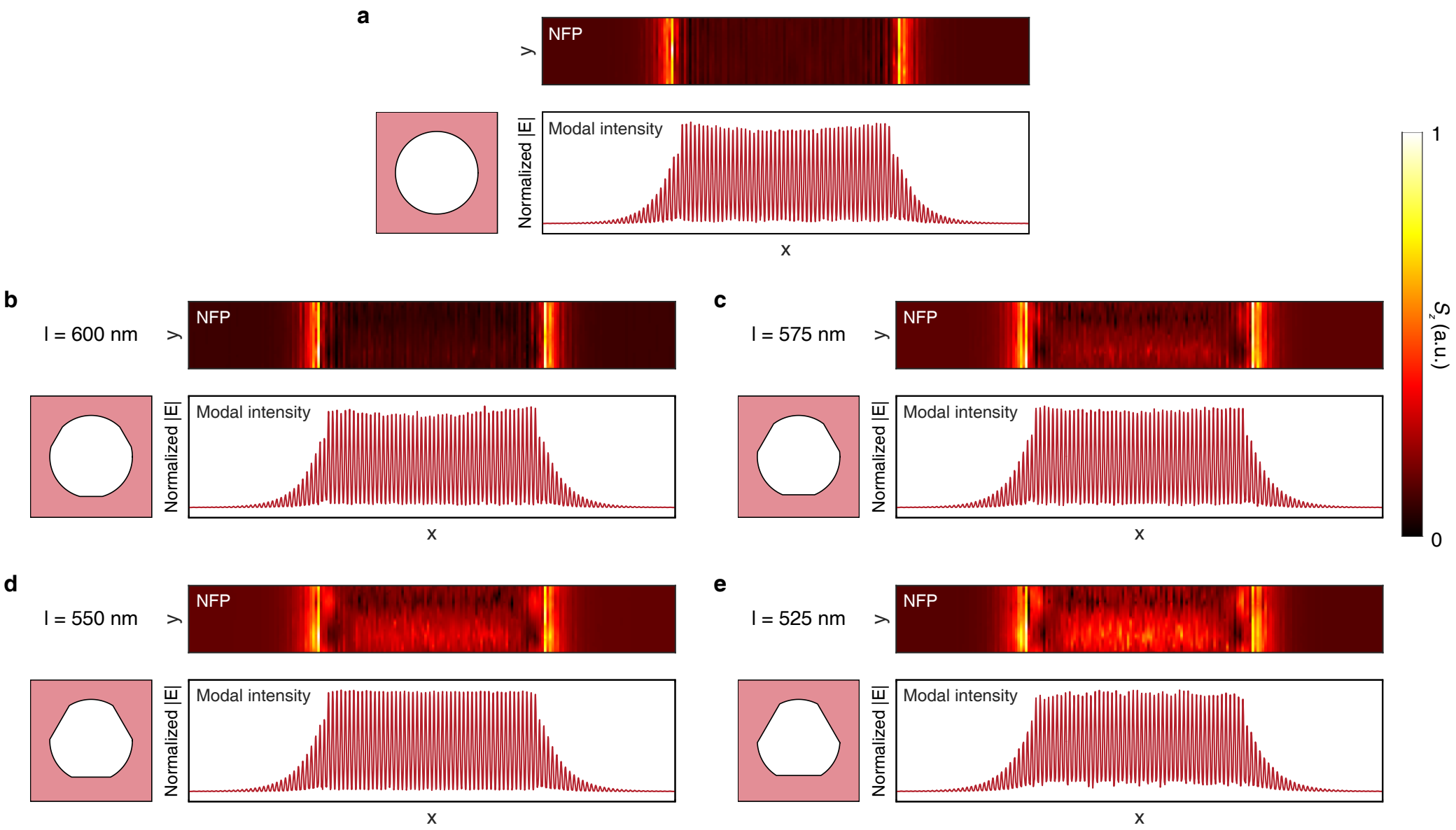

Figure R14 **Figure S12 in the Supplementary Material.** Achieving a bright flat NFP by increasing the degree of C_2-symmetry breaking.

COMMENT 3.D

Furthermore, in the mapping from the ring model to the two-dimensional photonic crystal, the functional relationship between the reflective phase (θ) and the gap spacing (g) is not explicitly provided in the main text and is only illustrated through simulation.

REPLY 3.D

Thanks for this insightful comment. In the ring model, there are only two counter-propagating plane waves, and thus the relative phase shift between them can be easily defined and derived. However, for a realistic 2D photonic crystal cavity with PBGs as boundaries, there are not the plane waves, but plenty of Bloch components mix and collectively oscillate to form a set of transverse cavity modes. And a variety of factors, including the group velocity of Bloch bands and the properties of the photonic band gap, would affect the boundary phase shift θ. As we reply to **comment 3.B**, we employed a semi-analytical CWT to investigate the coupling between Bloch components and thus calculate the effective θ for every given g. The details of CWT theory can be found in Supplementary Material Section 4, "Coupled-wave theory for homogeneous 2D PhC slab".

Moreover, for a bulk band near the Γ point, we derive a closed form of $\theta(g)$ (Eq. R9 and Eq. R10), by omitting all the high-order Bloch components with larger reciprocal wavevectors. This simplified model could give an approximate prediction upon the θ with respect to g. The detailed discussion could be found in our reply to **comment 3.B**, and we have updated the Supplementary Material (Section 7, "Theoretical model of $\theta - g$ relationship") to include this explicit deviation.

List of changes

Changes are highlighted in blue in the revised manuscript and supplementary information.

Changes in revised manuscript

- Changed the title to "Wave morphing towards the flat-top envelope in photonics systems driven by artificial gauge fields" and update the Abstract and "Introduction" to improve the clarity. ↦ Comment 1.A
- Added a brief discussion acknowledging the approach to introduce a continuous distributed permittivity modulation on page 6-7. ↦ Comment 1.A
- Clarified the distinctions between the simulation range and the experimental range of g on page 15. ↦ Comment 1.C
- Added a brief discussion of the single-mode lasing action on page 11. ↦ Comment 1.D
- Added discussions to address the distinction between the NFP and MEP on page 12-14. ↦ Comments 3.C and 1.E
- Updated Fig. 5 with new experimental results to directly demonstrate that controlled symmetry breaking releases bulk radiation and yields a flat, nonzero NFP. ↦ Comments 3.C and 1.E
- Clarified the distinction between the boundary-enhanced state and conventional non-Hermitian skin modes, placed at the end of "Principle and design" on page 9-10. ↦ Comment 2.C
- Added a concise qualitative remark with representative citations on higher-order topological BICs in "Conclusions" on page 16-17. ↦ Comment 2.D
- Adjusted font sizes in all figures to comply with the journal guidelines (5–7 pt), particularly in Figs. 3–5 and related panels. ↦ Comment 2.E

Changes in revised supplementary information

- Added Section 3, "Continuous gauge transformation and effective potential", discussing the effective gauge potential arising from continuous permittivity modulation and how it unifies boundary phase shifts with distributed permittivity engineering to enable flat-top mode formation. ↦ Comment 1.A
- Added Section 9, "Design of PhC for lasing" with lasing spectra (Figs. S10, S11) and discussion of mode-selection mechanism. ↦ Comment 1.D
- Added Section 10, "Interpreting near-field patterns and realizing uniform out-of-plane emission", discussing the distinction between the NFP and the actual modal envelope and practical routes toward device-ready flat-top emission. ↦ Comments 3.C and 1.E
- Added Section 8, "Non-Hermitian ring model" with detailed discussion, clarifying the relationship to non-Hermitian skin effect. ↦ Comment 2.C
- Added Section 7, "Theoretical model of $\theta - g$ relationship", providing a direct mapping between the reflection phase and the gap spacing through simulation and theory. ↦ Comments 3.B and 3.D

References

[R1] Peierls, R. E. *Quantum theory of solids* (Oxford university press, 1955).

[R2] Jimenez-Garcia, K. *et al.* Peierls substitution in an engineered lattice potential. *Phys. Rev. Lett.* **108**, 225303 (2012).

[R3] Aidelsburger, M., Nascimbene, S. & Goldman, N. Artificial gauge fields in materials and engineered systems. *C. R. Phys.* **19**, 394–432 (2018).

[R4] Mendez, E. E. & Bastard, G. Wannier-stark ladders and bloch oscillations in superlattices. *Phys. Today* **46**, 34–42 (1993).

[R5] Feldmann, J. *et al.* Optical investigation of bloch oscillations in a semiconductor superlattice. *Phys. Rev. B* **46**, 7252 (1992).

[R6] Garanovich, I. L., Longhi, S., Sukhorukov, A. A. & Kivshar, Y. S. Light propagation and localization in modulated photonic lattices and waveguides. *Phys. Rep.* **518**, 1–79 (2012).

[R7] Glück, M., Kolovsky, A. R. & Korsch, H. J. Wannier–stark resonances in optical and semiconductor superlattices. *Phys. Rep.* **366**, 103–182 (2002).

[R8] Mukherjee, S. *et al.* Modulation-assisted tunneling in laser-fabricated photonic wannier–stark ladders. *New J. Phys.* **17**, 115002 (2015).

[R9] Martijn de Sterke, C., Bright, J. N., Krug, P. A. & Hammon, T. E. Observation of an optical wannier-stark ladder. *Phys. Rev. E* **57**, 2365–2370 (1998).

[R10] Mukherjee, S. & Thomson, R. R. Observation of localized flat-band modes in a quasi-one-dimensional photonic rhombic lattice. *Opt. Lett.* **40**, 5443–5446 (2015).

[R11] Lumer, Y. *et al.* Light guiding by artificial gauge fields. *Nat. Photon.* **13**, 339–345 (2019).

[R12] Pankov, A. V., Vatnik, I. D., Churkin, D. V. & Sukhorukov, A. A. Observation of localized modes at effective gauge field interface in synthetic mesh lattice. *Sci. Rep.* **9**, 3464 (2019).

[R13] Chen, Z. *et al.* Observation of miniaturized bound states in the continuum with ultra-high quality factors. *Sci. Bull.* **67**, 359–366 (2022).

[R14] Chen, Y. *et al.* Observation of chiral emission enabled by collective guided resonances. *Nat. Nanotechnol.* (2025).

[R15] Yoshida, M. *et al.* Double-lattice photonic-crystal resonators enabling high-brightness semiconductor lasers with symmetric narrow-divergence beams. *Nat. Mater.* **18**, 121–128 (2019).

[R16] Ohnishi, D., Okano, T., Imada, M. & Noda, S. Room temperature continuous wave operation of a surface-emitting two-dimensional photonic crystal diode laser. *Opt. Express* **12**, 1562–1568 (2004).

[R17] Morita, R., Inoue, T., De Zoysa, M., Ishizaki, K. & Noda, S. Photonic-crystal lasers with two-dimensionally arranged gain and loss sections for high-peak-power short-pulse operation. *Nat. Photon.* **15**, 311–318 (2021).

[R18] Liang, Y., Peng, C., Sakai, K., Iwahashi, S. & Noda, S. Three-dimensional coupled-wave model for square-lattice photonic crystal lasers with transverse electric polarization: A general approach. *Phys. Rev. B* **84**, 195119 (2011).

[R19] Liang, Y., Peng, C., Sakai, K., Iwahashi, S. & Noda, S. Three-dimensional coupled-wave analysis for square-lattice photonic crystal surface emitting lasers with transverse-electric polarization: finite-size effects. *Opt. Express* **20**, 15945–15961 (2012).

[R20] Peng, C., Liang, Y., Sakai, K., Iwahashi, S. & Noda, S. Three-dimensional coupled-wave theory analysis of a centered-rectangular lattice photonic crystal laser with a transverse-electric-like mode. *Phys. Rev. B* **86**, 035108 (2012).

Reply to Referee 1

Reply to Referee 2

COMMENT 2.A

> I have read the authors' response and the revised manuscript carefully, and I am satisfied that the authors have adequately addressed the concerns raised in my previous review. In particular, the revised manuscript now provides a much clearer and more balanced discussion regarding the physical interpretation and practical implications of the flat-top ground states, especially in the context of lasing applications and gain–loss engineering. The distinction between the gauge-induced flat-top modes demonstrated here and conventional non-Hermitian skin modes has also been clarified substantially, which improves the conceptual positioning of the work within the broader non-Hermitian photonics literature.
>
> I also appreciate very much the authors' expanded discussion on the relation between the present platform and bound states in the continuum (BICs), including the possible extension toward topologically nontrivial systems.
>
> Overall, the revisions have significantly improved the clarity and presentation of the manuscript without altering its core contributions. In my opinion, the manuscript now represents a strong and elegant contribution combining clear theoretical insight with convincing experimental validation. I therefore support publication of this revised version in Nature Communications.

REPLY 2.A

> We thank the reviewer for supporting the publication of our work. We are especially grateful for the constructive suggestions on the physical interpretation and BIC connections, which have greatly helped us sharpen the presentation and contextualize our findings.

Reply to Referee 3

COMMENT 3.A

> The authors have fully considered and have provided well-organized responses to all comments. They adequately address concerns about the "θ–g relationship", the "quantitative validation of flat-top profiles", and the "interpretation of near-field patterns". And the additional discussion is clear and logic also for non-experts on the topic. The response demonstrates strong physical understanding, appropriately supplements theoretical modeling and experimental data, and aligns fully with the standards expected in a high-quality revision.

REPLY 3.A

> We thank the reviewer for supporting the publication of our work. We truly appreciate the careful assessment of our responses and the positive feedback on the clarity of the added discussions, which encourages us that the revised manuscript is now accessible to a broader audience.
